# Convergent Synthesis of Tetradentate Aminopyridine C–H Oxidation Catalysts

**DOI:** 10.1021/acsorginorgau.6c00006

**Published:** 2026-03-10

**Authors:** Yiheng Lu, Kanstantsin Anisovich, Konrad Tiefenbacher

**Affiliations:** † Department of Chemistry, University of Basel, Mattenstrasse 22, 4058 Basel, Switzerland; ‡ Department of Biosystems Science and Engineering, ETH Zürich, Klingelbergstrasse 48, 4056 Basel, Switzerland

**Keywords:** convergent synthesis, modular design, nonheme manganese catalysts, C−H oxidation, solvophobic effect, supramolecular catalysis

## Abstract

Controlling the oxidation of unactivated C­(sp^3^)–H bonds through supramolecular catalyst–substrate interactions has recently enabled site-selective transformations previously considered inaccessible. However, progress has been hampered by lengthy linear syntheses of supramolecular catalysts. Here we present a convergent strategy that directly cross-couples preformed tetradentate aminopyridine ligands with recognition motifs, streamlining access to functional catalysts. Using this approach, we prepared four catalysts, two featuring resorcin[4]­arene (RS) and two bearing calix[4]­arene (CX) units, for substrate recognition using the solvophobic effect in 2,2,2-trifluoroethanol. This convergent synthesis reduces the longest linear sequence from 5 steps to 2 steps (starting from the recognition motif) by shifting from a linear, catalyst-specific synthesis to a modular platform that allows rapid assembly of catalyst libraries using premade, interchangeable building blocks.

## Graphical abstract

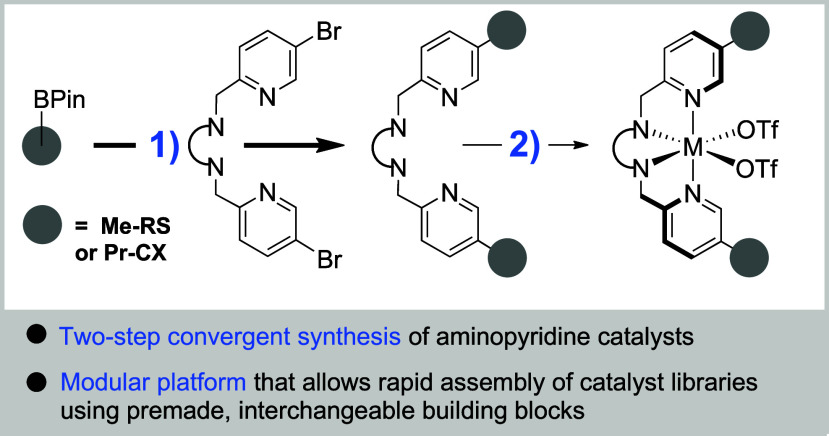

Significant progress has recently been achieved in controlling the oxidation of unactivated C­(sp^3^)–H bonds through supramolecular catalyst–substrate interactions. Covalent attachment of recognition motifs to state-of-the-art tetradentate iron and manganese aminopyridine complexes^1–4^ has enabled site-selective oxidation of unactivated and even deactivated C–H bonds in alkyl- and steroidal-ammonium substrates using hydrogen peroxide as a benign terminal oxidant.^5–8^ More recently, the solvophobic effect in highly polar fluorinated alcohols, a solvent of choice for C–H oxidations,^9,10^ has been harnessed as a driving force for substrate binding,^11^ a modality hitherto overlooked in catalyst-directed transformations. This strategy enabled the selective oxidation of alkanes at medial positions,^12^ a breakthrough given that, even within the broader field of C–H functionalization,^13–15^ site-selective derivatization of alkanes at medial sites has remained an unsolved challenge.^16–20^ Supramolecular catalyst-directed oxidation therefore represents a powerful tool to expand the site-selectivity landscape of C–H oxidation, overcoming the inherent constraints of traditional catalysts, which typically address only a narrow subset of reactive sites.^1,21–24^

The concept of recognition-driven site-selectivity in C–H oxidation was pioneered by Breslow and co-workers, who employed porphyrin catalysts together with iodosobenzene, a less practical oxidant than hydrogen peroxide.^25,26^ Its broader applicability, however, remained limited, as the substrates required at least two functional groups, which furthermore had to be covalently modified before the oxidation. Later studies by Crabtree and Brudvig^27,28^ and by Bach,^29–31^ succeeded in utilizing hydrogen bonds between substrate and catalyst for the regioselective and even enantioselective C–H oxidation but were restricted to activated benzylic C–H bonds.

Advances in the field have been impeded by the lengthy linear synthetic routes required to access nonheme C–H oxidation catalysts with defined supramolecular recognition motifs, which substantially limit systematic studies and broader applicability. The synthesis of supramolecular catalysts such as **1** (Figure 1a), featuring the tetradentate iron and manganese aminopyridine complexes that have emerged as superior catalysts for the oxidation of nonactivated C–H bonds,^1–4^ relied exclusively on a catalyst-specific linear strategy. The tetradentate aminopyridine catalyst is constructed over four to five linear steps starting from the recognition motif **2**, making the overall process labor-intensive and time-consuming.^5,7,12,32^ In an ideal scenario, compound **2** would be directly cross-coupled to the preformed tetradentate ligand **3** (Figure 1b), enabling a more convergent synthetic strategy that allows rapid assembly of catalyst libraries from premade, interchangeable building blocks. Surprisingly, this approach has not been previously reported. Interestingly, during the preparation of this manuscript, Wang, Nam, and colleagues disclosed that anthracene appendages can be attached via such a strategy.^33^ However, the reported conditions were inadequate for the installation of supramolecular recognition units such as resorcin[4]­arene (RS) and calix[4]­arene (CX) for the recognition of alkyl substrates. We surmise that this strategy was previously disregarded because tetradentate ligand **3** was expected to impede palladium-catalyzed coupling reactions through undesired coordination. Nevertheless, the approach proved viable, and we report its successful implementation.

**Figure 1:**
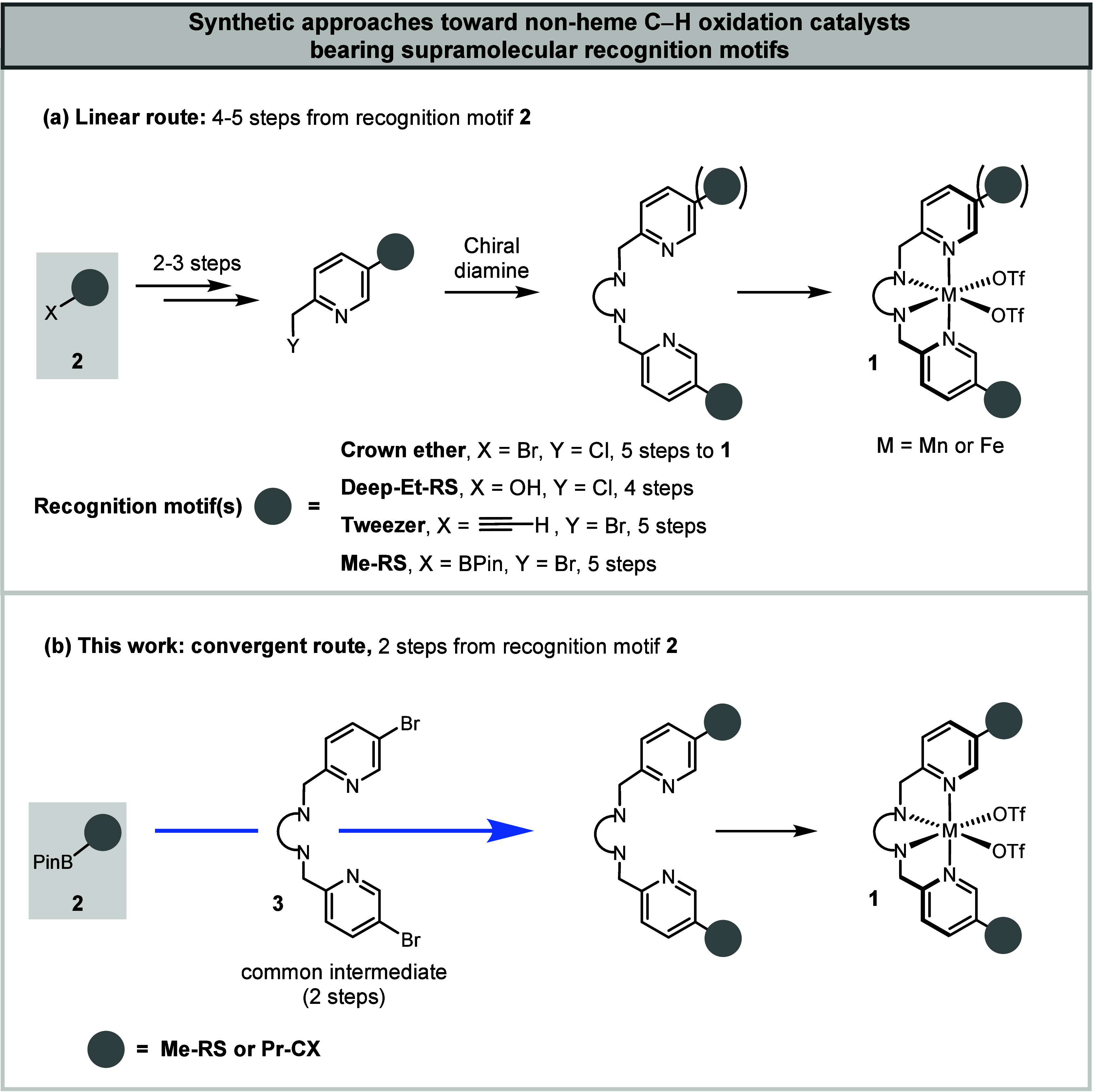
(a) Traditional linear route to nonheme C–H oxidation catalysts bearing supramolecular recognition motifs. (b) This work: rapid, convergent synthesis of supramolecular C–H oxidation catalysts. Resorcin[4]­arene (RS), calix[4]­arene (CX).

In this study, the convergent syntheses of four tetradentate aminopyridine-based supramolecular C–H oxidation catalysts, the novel Mn­(*S,S*-pdp)-RS_2_ (**4**, *S,S*-pdp = 2-({(*S*)-2-[(*S*)-1-(pyridin-2-ylmethyl)­pyrrolidin-2-yl]­pyrrolidin-1-yl}­methyl)­pyridine), Mn­(*S,S*-pdp)-CX_2_ (**5**), Mn­(*S,S*-mcp)-CX_2_ (**6**, *S,S*-mcp = *N*,*N′*-dimethyl-*N*,*N′*-bis­(2-pyridylmethyl)-cyclohexane-1*S*,2*S*-diamine), and the previously disclosed Mn­(*S,S*-mcp)-RS_2_ (**7**),^12^ were developed (Scheme 1). The two-step synthesis of the common dibromo-backbone **8** and **9** commenced from commercially available (5-bromo-2-pyridinyl)­methanol (**10**), which was first converted to the benzylic bromide **11** followed by alkylation of the commercially available diamine **12** or **13**, respectively. The subsequent Suzuki cross-coupling had to be carefully optimized (see details below). Under optimized conditions, coupling with the recognition motifs BPin-RS **14** or BPin-CX **15** afforded the corresponding diRS- and diCX-functionalized aminopyridine ligands (step 1a: (*S,S*-pdp)-RS_2_ (**16**) and (*S,S*-pdp)-CX_2_ (**17**), step 1b: (*S,S*-mcp)-CX_2_ (**18**) and (*S,S*-mcp)-RS_2_ (**19**)). The supramolecular C–H oxidation catalysts **4**–**7** were obtained after coordination of the Mn­(II)-metal center (steps 2a–d).

**Scheme 1:**
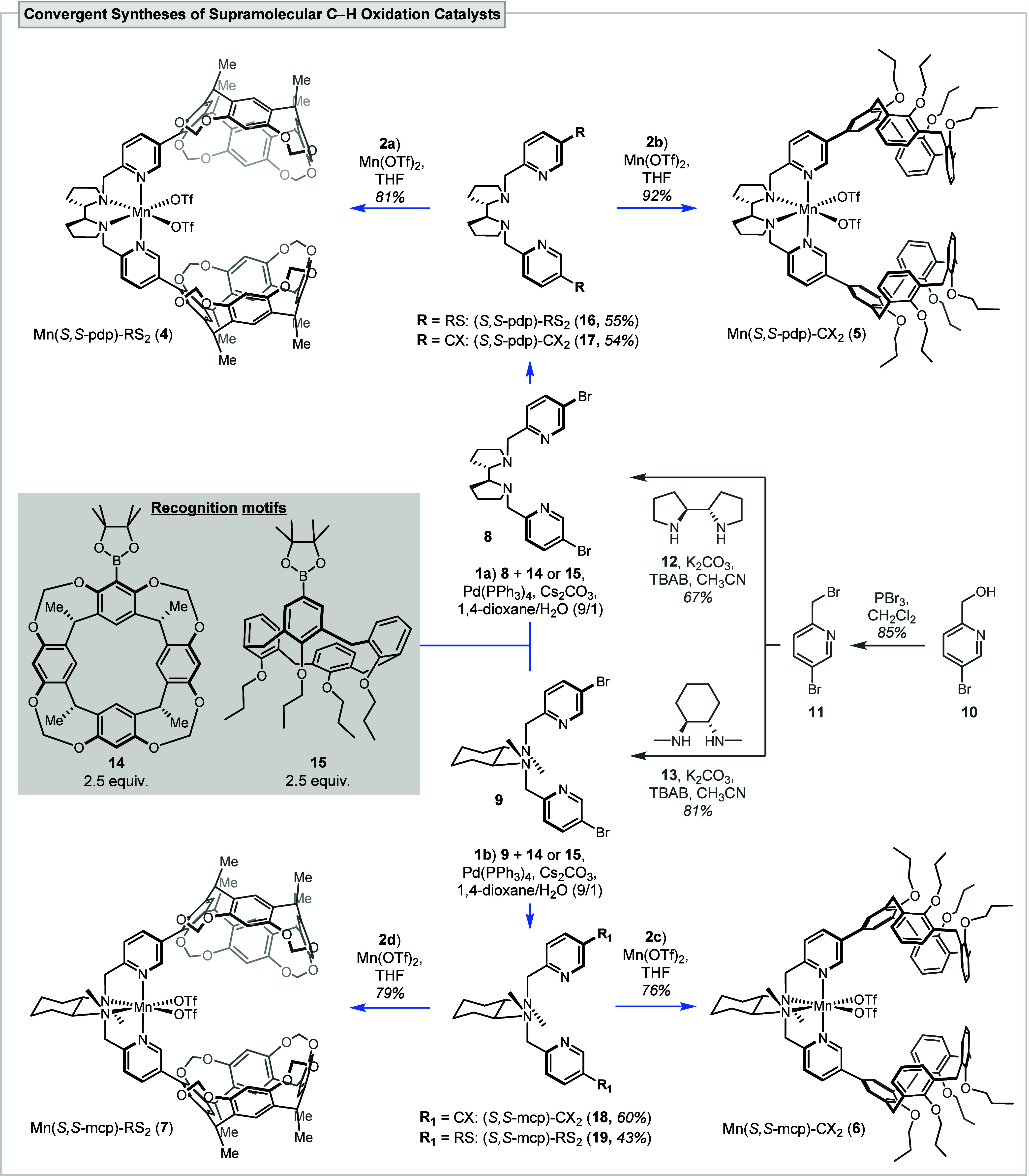
Convergent Synthesis Route to Construct Supramolecular Catalysts **4**–**7**

The reaction conditions of the crucial cross-coupling step were first screened with the backbone (*S,S*-mcp)-diBr (**9**), and the findings are summarized in Table 1. Conditions analogous to those employed in our previous work for the linear installation of the pyridine moiety onto the RS recognition motif served as the starting point of this investigation (entry 1, NMR yield (Y) = 32% of diarylated product, determined by NMR with tetraethylsilane as an internal standard).^12^ In the solvent mixture of dimethylformamide/water (9/1 v/v%) neither higher catalyst loading (entry 2) nor variation of the Pd catalyst (entry 3) led to an increase in yield of the desired diarylated product **19**. The first notable hit was observed upon switching the solvent to a 9:1 mixture of 1,4-dioxane/water and replacing potassium carbonate with cesium carbonate as the base (entry 4, NMR Y = 59 ± 9.8% of **19**, results from three experiments). Raising the temperature proved unfavorable, as it increased the formation of the defunctionalized side product **SI-1** (see SI, Bpin of **14** → H, entries 5 and 6). Attempts to reduce the catalyst loading likewise resulted in lower yields of desired ligand **19** (entries 7 and 8). Applying the conditions of entry 4 to a 3-fold scale-up, the diRS-functionalized ligand **19** was isolated in 43% yield (NMR Y = 54%). The successful but challenging separation of **19** from triphenylphosphine oxide (TPPO) is described in the Supporting Information.

**Table 1:** Condition Screening for Suzuki Cross-Coupling of diBr-Ligand **9** to Bpin-RS **14** ^a^

Entry	Pd cat. (mol %)	Scale of **9** (mg)	Base (4.5 equiv)	Solvents (v/v %)	*T* (°C)	Diaryl. (NMR Y, %)	Monoaryl.^b^ (NMR Y, %)	Isolated Y (%)
1	Pd(PPh_3_)_4_ (17)	5	K_2_CO_3_	DMF	100	32	0	**-**
2	Pd(PPh_3_)_4_ (20)	5	K_2_CO_3_	DMF/H_2_O (9/1)	100	34	0	**-**
3	Pd(dppf)Cl_2_ (20)	5	K_2_CO_3_	DMF/H_2_O (9/1)	100	35	13	**-**
4	Pd(PPh_3_)_4_ (20)	5	Cs_2_CO_3_	dioxane/H_2_O (9/1)	100	59 ± 9.8^c^	0	**-**
5	Pd(PPh_3_)_4_ (20)	5	Cs_2_CO_3_	dioxane/H_2_O (9/1)	105	31	13	**-**
6	Pd(dppf)Cl_2_ (20)	5	Cs_2_CO_3_	dioxane/H_2_O (9/1)	105	40	0	**-**
7	Pd(PPh_3_)_4_ (10)	5	Cs_2_CO_3_	dioxane/H_2_O (9/1)	100	5	18	**-**
8	Pd(PPh_3_)_4_ (15)	5	Cs_2_CO_3_	dioxane/H_2_O (9/1)	100	23	24	**-**
9	Pd(PPh_3_)_4_ (20)	15	Cs_2_CO_3_	dioxane/H_2_O (9/1)	100	54	0	43

a 3 day reaction time. Arylated (aryl.).

b Monoaryl. species = (*S,S*-mcp)-RSBr (a RS unit appended on one pyridine moiety and a bromide remained on the other). Yield (Y).

c Average of three experiments.

The optimized Suzuki cross-coupling conditions used to access ligand (*S,S*-mcp)-RS_2_ (**19**, Table 1, entry 4) were directly applied to the synthesis of (*S,S*-pdp)-RS_2_ (**16**) and to the installation of the calix[4]­arene recognition motif **15**, yielding ligands **17** and **18** in 43–60% yield (Scheme 1) without further condition optimization, highlighting the broad applicability of this convergent ligand-construction strategy.

With the three novel catalysts in hand, their performance was evaluated relative to that of the unsubstituted core catalysts **20** and **21** using four benchmark substrates (**S1–4**, Scheme 2). It is important to note that only the GC yields (Y) of the hydroxylated products and the corresponding ketones are given, and only these two products were taken into account in the study on the site-selectivity of the C–H oxidation reaction. Further details on the side products (epoxides and esters) were discussed in our previous work.^12^ Using the previously reported conditions with the additive 2,2-dimethylpropanoic acid (2,2-diMe-PA) and the polar solvent 2,2,2-trifluoroethanol (TFE),^12^ both RS-based supramolecular catalysts **4** and **7** (highlighted in blue) exhibit comparable conversion and yield and, most importantly, a similar preference for oxidation at the fifth carbon (red sphere), counted from the less hindered terminus of the substrate (gray dot). **S3** represents the sole exception, where the more reactive tertiary C(3)–H (red dotted circle, 3 denotes the third position on the carbon framework) lies adjacent to the fifth-position methylene group. The results indicate that the site-selectivity (S), driven by the solvophobic RS recognition motifs, is largely independent of the two distinct cores (mcp and pdp). Both catalysts are capable of (1) differentiating between three chemically similar secondary C­(sp^3^)–H bonds in **S1**, (2) overriding the intrinsic reactivity of C–H bonds in **S2**, (3) predominantly oxidizing the electronically deactivated proximal C(3)–H bond over the electron-rich distal C(7)–H on **S3**, and (4) favoring the sterically congested, tertiary C(4)–H bond in **S4** that is hardly oxidized at all by the unsubstituted catalysts **20** and **21**.

**Scheme 2:**
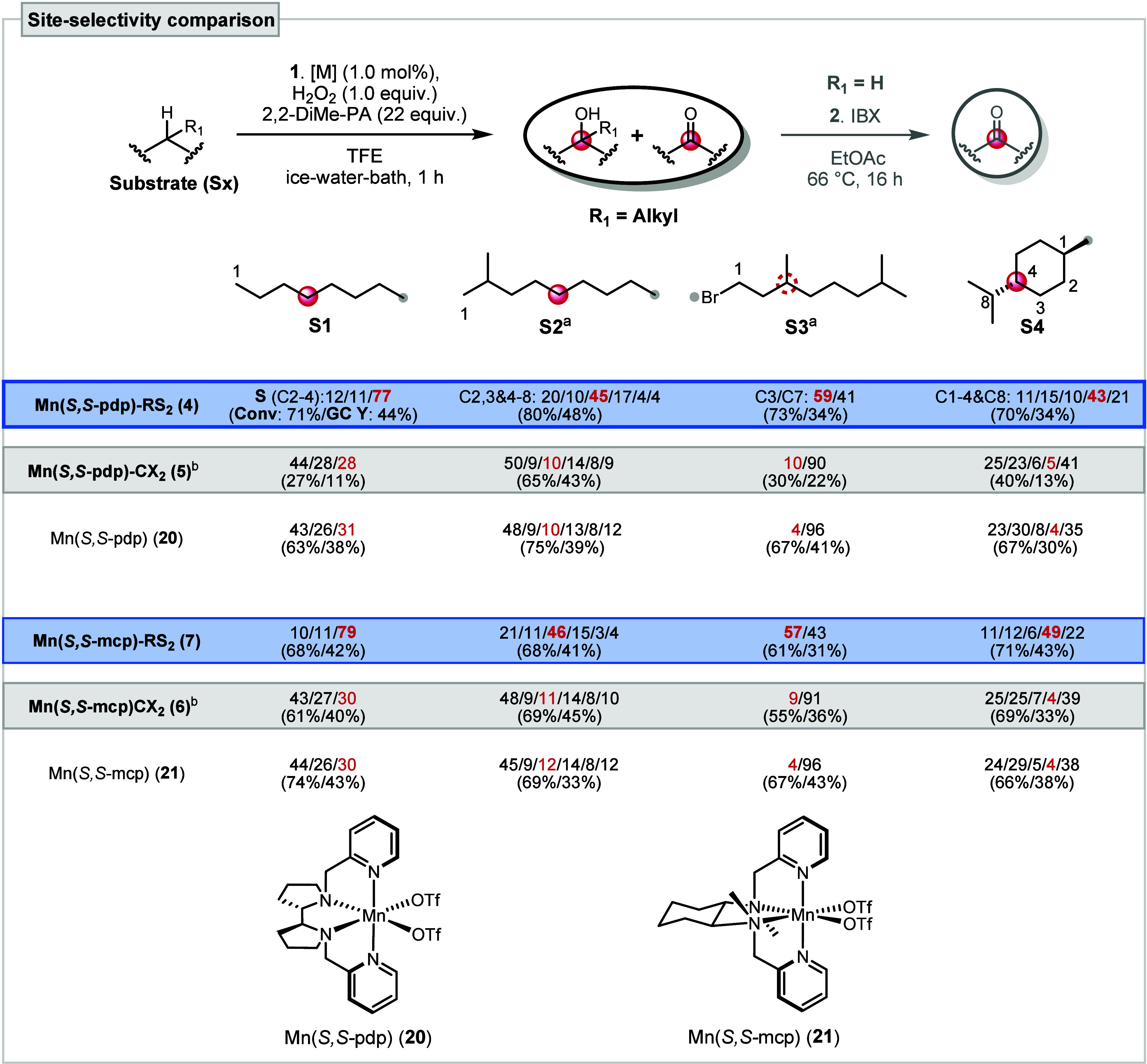
Comparison of Catalyst-Directed Site-Selectivity in C–H Oxidation of Selected Substrates^a^

a 5.0 equiv of 2,2-DiMe-PA used.
b 2.5 mol % of trifluoromethanesulfonic acid added.
c Oxidation with 2,2-dimethylpropanoic acid (2,2-diMe-PA) in 2,2,2-trifluoroethanol (TFE); 2-iodoxybenzoic acid (IBX) oxidation was performed to facilitate the identification of the oxidation products. Site-selectivity (S) and conversion (conv.) were calculated based on remaining substrate. GC yield (Y) equals the sum of the alcohols or/and ketones.

As for the CX-appended catalysts **5** and **6** (marked in gray), interestingly the observed site-selectivity did not differ significantly from the one shown by the unsubstituted parent catalysts **20** and **21**. Screening of additional carboxylic acid additives and more polar solvents likewise resulted in no improvement (SI Section 3.2). Notably, while both CX and RS macrocycles can freely rotate around the pyridine–aryl C–C bond, only the CX catalysts show no change in selectivity. This suggests that the observed behavior arises from inherent structural features of the cavitands rather than conformational flexibility. Specifically, the smaller and less preorganized cavity of the CX motif is expected to bind substrates more weakly than RS. In terms of catalytic activity, both CX catalysts require a second acid additive, trifluoromethanesulfonic acid (TfOH), to be active. This can be attributed to the reported ability of Brønsted acids to assist in the heterolytic cleavage of the O–O bond to form the strongly oxidizing high-valent metal oxo species.^34,35^ Nevertheless, in the case of **5**, the activity was still substantially lower compared to that of the RS counterpart **4** and to that of the parent **20**. By reduction of the equivalents of the carboxylic acid additive from 22 to 5, catalyst **5** achieved comparable activity with substrate **S2**. However, the same amount of carboxylic acid did not lead to a higher activity in the instance of **S3**. On the other hand, the addition of TfOH aided catalyst **6** in attaining consistent good catalytic activity in comparison to **7** and **21**.

In summary, we have developed a concise convergent strategy that allows rapid assembly of catalyst libraries using premade, interchangeable building blocks. This approach overcomes the limitations of previous linear, catalyst specific routes, streamlining the synthesis of catalysts such as Mn­(*S,S*-pdp)-RS_2_ (**4**), Mn­(*S,S*-pdp)-CX_2_ (**5**), Mn­(*S,S*-mcp)-CX_2_ (**6**), and Mn­(*S,S*-mcp)-RS_2_ (**7**). The screening results of the obtained novel catalysts indicated that the backbone (mcp or pdp) of the RS-based catalysts **4** and **7** does not influence the recognition-driven oxidation selectivity for the fifth position on alkyl substrates that greatly differs from the selectivity observed with unfunctionalized catalysts **20** and **21**. Furthermore, we demonstrated that closely related catalysts featuring CX recognition motifs do not enable selective C–H oxidation. The convergent route developed in this study provides a broadly applicable platform for building diverse catalyst libraries. We expect this route to facilitate a systematic exploration of site-selective C–H oxidation across a wide range of substrates and catalysts.

## Supplementary Material



## Data Availability

The data underlying this study are available in the published article, in its Supporting Information, and openly available in Zenodo:18300467 at https://zenodo.org/records/18300467.

## Notes

Experimental procedures, analytical data of the compounds, GC- and GC-MS chromatograms, and NMR spectra (PDF)
