# Supplementary material for: Convergent Synthesis of Tetradentate Aminopyridine C–H Oxidation Catalysts
Source: ACS Org Inorg Au. 2026 Mar 10;6(2):202–8. doi: 10.1021/acsorginorgau.6c00006 (PMC13047453; doi:10.1021/acsorginorgau.6c00006)

Supplemental Information

Convergent Synthesis of Tetradentate Aminopyridine C–H Oxidation Catalysts

Yiheng Lu,[1] Kanstantsin Anisovich,[1] Konrad Tiefenbacher[1,2,3]

[1]Department of Chemistry, University of Basel, Mattenstrasse 22, 4058 Basel, Switzerland
[2]Department of Biosystems Science and Engineering, ETH Zürich, Klingelbergstrasse 48, 4056 Basel, Switzerland
[3]Lead Contact, Correspondence: konrad.tiefenbacher@unibas.ch (K.T.)

Table of Contents

Supplemental Methods S1

1. General Information S1

2. Syntheses of Catalysts S3

2.1 Synthesis of Mn(S,S-mcp)-RS$_2$ (7) S4

2.2 Synthesis of Mn(S,S-pdp)-RS$_2$ (4) S9

2.3 Synthesis of Mn(S,S-pdp)-CX$_2$ (5) S12

2.4 Synthesis of Mn(S,S-mcp)-CX$_2$ (6) S16

3. C−H Oxidation Studies S18

3.1 General Oxidation Procedure S18

3.2 Solvent and Carboxylic Acid Screening with CX-catalysts 5 and 6 .... S19

3.3 Detailed Oxidation Results S20

4. Supplemental References S35

Supplemental Data S37

Data S1: NMR Spectra S37

Supplemental Methods

1. General Information

Experimental: Reactions were carried out under an atmosphere of argon in dried glassware (in oven or with a heat-gun) unless otherwise indicated. Analytical thin-layer chromatography (TLC) was performed on Merck silica gel 60 F254 glass-backed plates, which were analyzed after exposure to standard staining solution (CAM: cerium ammonium molybdate). Medium Pressure Liquid Chromatography (MPLC, hereafter referred to as flash column chromatography) was carried out with RediSep® Silica Gel Disposable Flash Columns (SiO_2 particle size 40-60 μm) and Al_2O_3 basic columns (particle size 40-60 μm) on a CombiFlash NextGen 300+ version 5.0.55 by Teledyne ISCO with a fraction collector version 00.92.00, detector version 11, and a pump version: 1.47. All NMR experiments were performed on a Bruker Ascend 500 spectrometer operating at 500 MHz proton frequency, equipped with a direct observe 5-mm BBFO smart probe with actively shielded z-gradients (10 A). The experiments were performed at 298 K. Chemical shifts of ^{1}H-NMR and ^{13}C-NMR are given in ppm. The following solvent residual signals of the deuterated solvents were used as reference: $CDCl_3$: 7.26 ppm (δ^{1}H), 77.16 ppm (δ^{13}C). Coupling constants (*J*) are reported in Hertz (Hz). Standard abbreviations indicating multiplicity were used as follows: s (singlet), d (doublet), t (triplet), dd (doublet of doublets), m (multiplet). Infrared spectra were measured on a Brucker Alpha IR spectrometer (attenuated total reflection, ATR). Abbreviations indicating intensity were used as follows: s (strong), m (medium), w (weak). Melting points were recorded on a Büchi Melting Point M-565 apparatus using open capillary tubes. Gas chromatography (GC) analyses were carried out on a Shimadzu GC-2010 Plus instrument equipped with a flame ionization detector (FID) and an achiral Rtx-5 capillary column (length = 30 m). Hydrogen was used as the carrier gas and the linear velocity mode was used (pressure = 106.9 kPa, total flow = 64.1 mL/min, linear velocity = 69.8 cm/s) with a split ratio of 1:20. Unless else specified, the following temperature program was used: 60 °C for 3 min, 15 °C/min to 250 °C, and held at 250 °C for 5 min. For difficult to separate compounds, the chiral column MEGA-DEX DAC β capillary column (length = 60 m) was used (linear velocity mode: pressure = 177.6 kPa, total flow = 68.7 mL/min, linear velocity = 57.4 cm/s). Unless else specified, the following temperature program was used: 5 °C/min from 50 to 120 °C, held for 6 min, then 20 °C/min to 220 °C, held for 3 min). The response factors of oxidation products were calculated according to the literature.[1,2] GC-MS analyses were performed on Agilent 7890B gas chromatograph equipped with electron capture detector (G3442B), combined with mass spectrometer 5977B (ionization: electron impact (EI), detector G7081B). The achiral GC capillary column Agilent J&W HP-5MS UI (length = 30 m) was equipped and helium was used as the carrier gas (split ratio = 50:1, constant flow = 3.25 mL/min, pressure = 26.6 psi, average velocity = 66 cm/s). Same temperature program as on the Shimadzu GC-2010 Plus with achiral column was employed to achieve similar retention times on both columns, unless else specified. High-resolution mass spectra (HRMS) were obtained on a Bruker maxis 4G (ESI-Q-TOF) (ESI source parameters for positive polarity mode were: Set capillary: 4.5 kV, Set Ion Energy 4.0 eV).

Transfer of liquids with a volume ranging from 1 to 10 μL or from 10 to 100 μL was performed with a microman M1 pipette (Gilson, systematic error: 1.40% - 1.60%) equipped with 10 μL or 100 μL pipette tips, respectively. 4- and 12-mL screw neck vials were purchased from Fisher Scientific.

Sources of Solvents: Deuterated chloroform ($CDCl_3$, 99.8%, stabilized over silver foil) was purchased from Eurisotop. High pressure liquid chromatography (HPLC) grade dichloromethane (CH_2Cl_2), cyclohexane and ethyl acetate were bought from Sigma-Aldrich. HPLC-grade Acetonitrile (CH_3CN), methanol (MeOH), were bought from VWR. Anhydrous CH_3CN, CH_2Cl_2, diethyl ether (Et_2O), *N,N*-dimethylformamide (DMF), 1,4-dioxane, DMSO, MeOH, tetrahydrofuran (THF), hexane and toluene were purchased from Thermo Scientific. 2,2,2-trifluoroethanol (TFE), 1,1,1,3,3,3-hexafluoroisopropanol (HFIP) and nonafluoro-*tert*-butyl alcohol (NFTBA) were bought from Fluorochem.

Sources of Chemicals: tetrabutylammonium bromide, bis(triphenylphosphine)palladium(II)chloride, potassium acetate, tetrakis(triphenylphosphine)-palladium, triethyl amine, hydrogen peroxide, biphenyl, octane, (2*S*,2'*S*)-2,2'-bipyrrolidine, sodium hydride 60 % dispersion in mineral oil, 4,4′-di-

tert-butyl-2,2′-dipyridyl, $[Ir(OMe)(1,5\text{-}COD)]_2$ and 1-bromo-3,7-dimethyloctane were purchased from Sigma-Aldrich. Potassium carbonate, 4-*tert*-butylcalix[4]arene, aluminum chloride, phenol, 1-bromopropane and 2,2-dimethylpropanoic acid were bought from ThermoFisher Scientic/Acros Organics. Manganese bis(trifluoromethanesulphonate) and bis(pinacolato)diboron were purchased from Apollo Scientific. (1S,2S)-*N*,*N'*-Dimethyl-1,2-cyclohexane-diamine, cesium carbonate and (5-bromopyridin-2-yl)methanol were bought from Fluorochem. Phosphorus tribromide was bought from TCI. Sodium hydroxide, sodium chloride, hydrochloric acid (37 wt%), anhydrous sodium sulphate, sodium hydrogencarbonate and ammonium chloride were purchased from VWR. Celite® 545 (particle size 0.02-0.1 mm) was purchased from Merck KGaA. All chemicals were used as received.

2. Syntheses of Catalysts

C(sp^3)−H oxidation catalysts investigated in this work:

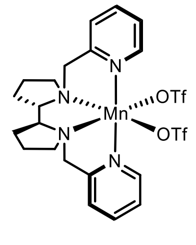

Mn(*S,S*-pdp)

20

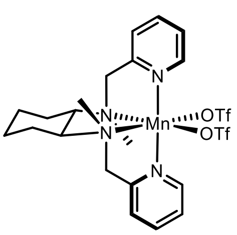

Mn(*S,S*-mcp)

21

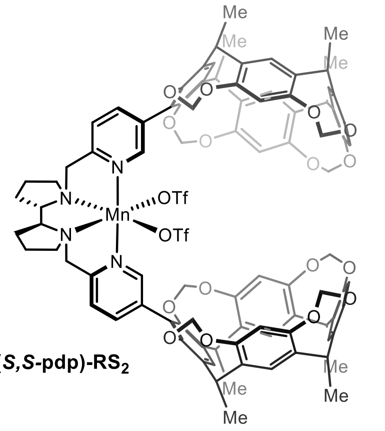

Mn(*S,S*-pdp)-RS$_2$

4

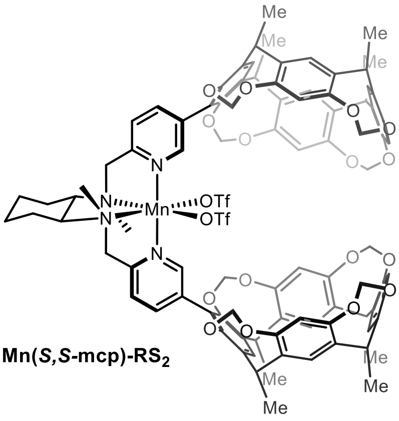

Mn(*S,S*-mcp)-RS$_2$

7

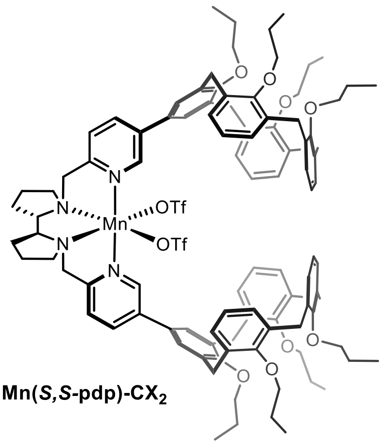

Mn(*S,S*-pdp)-CX$_2$

5

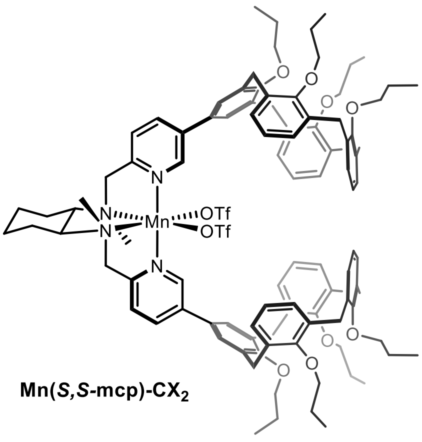

Mn(*S,S*-mcp)-CX$_2$

6

The unsubstituted parent catalysts Mn(*S,S*-pdp) (**20**)[3] and Mn(*S,S*-mcp) (**21**)[4] were synthesized according to literature procedures. Resorcin[4]arene is hereafter abbreviated as RS and calix[4]arene as CX. *S,S*-mcp stands for *N*,*N′*-dimethyl-*N*,*N′*-bis(2-pyridylmethyl)-cyclohexane-1*S*,2*S*-diamine and *S,S*-pdp for 2-({(*S*)-2-[(*S*)-1-(pyridin-2-ylmethyl)pyrrolidin-2-yl]pyrrolidin-1-yl}methyl)pyridine.

2.1 Synthesis of Mn(*S,S*-mcp)-RS$_2$ (7)

5-(4,4,5,5,-Tetramethyl-1,3,2-dioxaborolan-2-yl)-4(24),6(10),12(16),18(22)-tetramethylenedioxy-2,8,14,20-tetramethyl-resorcin[4]arene (14)

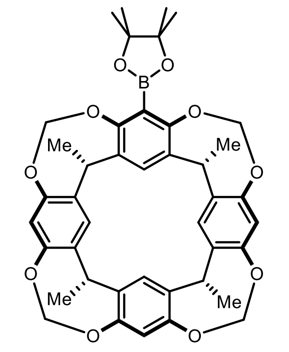

Chemical Formula: $C_{42}H_{43}BO_{10}$
Molecular Weight: 718.61

14

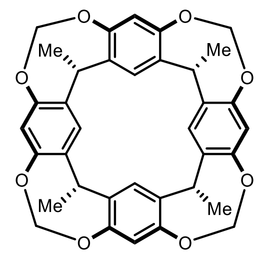

Chemical Formula: $C_{36}H_{32}O_8$
Molecular Weight: 592.64

SI-1

The synthesis of 5-(4,4,5,5,-tetramethyl-1,3,2-dioxaborolan-2-yl)-4(24),6(10),12(16),18(22)-tetramethylenedioxy-2,8,14,20-tetramethyl-resorcin[4]arene (**14**) as an inseparable mixture with the side product, methylene-bridged RS **SI-1**, was previously reported.[4] *Suzuki* cross-coupling reactions carried out with said mixtures with purity <80% often led to unrepeatable outcomes. Therefore, the separation of these two RS derivatives was reinvestigated. As the previously used RediSep® silver silica gel disposable flash columns (particle size 40-60 µm) were unable to perform this challenging separation, the gold version with smaller SiO_2 particle size of 20-40 µm was used and desired product **14** was isolated successfully.

Note: It was observed that the formation of **SI-1** can be minimized by additionally drying the solvent 1,4-dioxane with molecular sieves during degassing and using oven-dried KOAc (120 °C, 16 h) for the reaction.

Mp: Decomposition started at 261 °C.

IR of 14 (ATR, $\tilde{v}$/cm^{-1}): 2975 (w), 2937 (w), 1590 (w), 1491 (m), 1354 (m), 1305 (m), 1285 (s), 1250 (m), 1181 (s), 1135 (m), 1096 (s), 1023 (m), 980 (s), 947 (s), 909 (s), 849 (m), 727 (s).

^{1}H-NMR of 14 (500 MHz, 298 K, $CDCl_3$, δ/ppm): 7.29 (s, 1H), 7.22 (s, 1H), 7.21 (s, 2H), 6.54 (s, 1H), 6.44 (s, 2H), 5.74 (d, *J* = 7.1 Hz, 2H), 5.69 (d, *J* = 7.2 Hz, 2H), 4.98-4.92 (m, 4H), 4.52 (d, *J* = 7.1 Hz, 2H), 4.41 (d, *J* = 7.2 Hz, 2H), 1.76-1.75 (m, 12H), 1.32 (s, 12H).

^{13}C{^{1}H}-NMR of 14 (126 MHz, 298 K, $CDCl_3$, δ/ppm): 156.9, 154.7, 154.5, 154.4, 139.4, 139.3, 139.3, 139.1, 121.9, 120.2, 120.0, 116.8, 116.6, 99.5, 99.5, 84.5, 30.8, 30.7, 24.9, 16.0, 15.9.

5-bromo-2-(bromomethyl)pyridine (11)

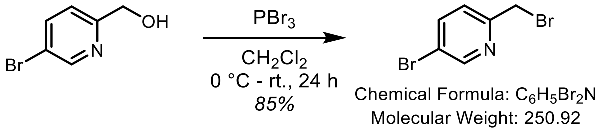

10 **11**

The synthesis of 5-bromo-2-(bromomethyl)pyridine (**11)** was inspired by a literature procedure.[5] A heat-gun dried round bottom flask, under an argon atmosphere equipped with stirring bar, was charged with (5-bromopyridin-2-yl)methanol (**10**, 500 mg, 2.66 mmol, 1.0 equiv.) and anhydrous dichloromethane (20 mL). The resulting solution was cooled down in an ice-water bath and PBr_3 (0.70 mL, 7.45 mmol, 2.8 equiv.) and the reaction mixture was allowed to warm up to room temperature and stirred for 24 h. Upon completion, saturated aqueous $NaHCO_3$ solution (20 mL) was added to quench the remaining PBr_3 and the mixture was stirred for 1 h under vigorous stirring. Then, the mixture was extracted with dichloromethane (3 × 20 mL). The combined organic layers were washed with water (2 × 20 mL) and brine (20 mL), dried over Na_2SO_4 and filtered. The solvent was removed under reduced pressure to yield **11** (570 mg, 2.27 mmol, 85%) as a light pink oil that turns into dark brown solid over time. The crude material was subjected to the subsequent step without further purification.

^{1}H-NMR (500 MHz, 298 K, $CDCl_3$, δ/ppm): 8.63 (dd, J = 2.4, 0.72 Hz, 1H), 7.82 (dd, J = 8.3, 2.4 Hz, 1H), 7.35 (dd, J = 8.3, 0.72 Hz, 1H), 4.50 (s, 2H).

^{13}C{^{1}H}-NMR (126 MHz, 298 K, $CDCl_3$, δ/ppm): 155.5, 150.9, 139.9, 124.9, 120.3, 32.9.

The ^{1}H-NMR data match with the literature values.[6]

(1*S*,2*S*)-*N*^{1},*N*^{2}-bis((5-bromopyridin-2-yl)methyl)-*N*^{1},*N*^{2}-dimethylcyclohexane-1,2-diamine = (*S*,*S*-mcp)-diBr (9)

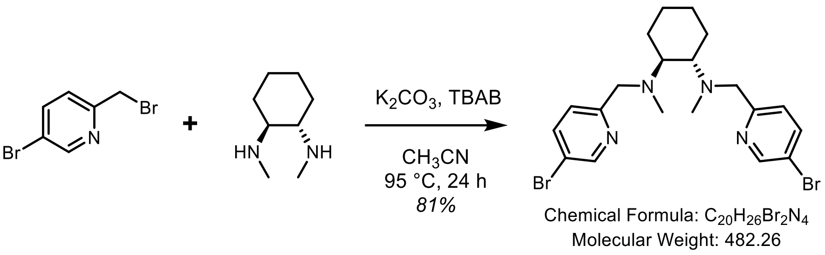

11 **13** **9**

The synthesis of ligand (*S,S*-mcp)-diBr (**9**) was carried out following an adapted literature procedure for the synthesis of *S,S*-mcp.[4] A heat-gun dried round bottom flask, equipped with reflux condenser and stirring bar under an argon atmosphere, was charged with **11** (286 mg, 1.14 mmol, 2.5 equiv.)*, potassium carbonate (284 mg, 2.05 mmol, 4.5 equiv.), tetrabutylammonium bromide (TBAB, 30.9 mg, 95.7 µmol, 0.21 equiv.), (1*S,*2*S*)-*N,N'*-dimethyl-1,2-cyclohexanediamine (**13**, 72 µL, 64.9 mg, 456 µmol, 1.0 equiv.) and anhydrous acetonitrile (25 mL). The reaction mixture was heated to reflux at 95 °C (oil bath temp.) and stirred for 24 h. After allowing to cool down to room temperature, the reaction mixture was filtered and rinsed with dichloromethane (10 mL). The filtrate was concentrated *in vacuo* and NaOH solution (1.0 M, 10 mL) and water (10 mL) were added to the residue and the mixture was extracted with dichloromethane (3 × 20 mL). The combined organic layers were washed with brine (20 mL), dried over Na_2SO_4 and filtered. The solvent was removed under reduced pressure and the resulting crude material was purified by flash column chromatography (A: cyclohexane, B: ethyl acetate, gradient: 0-100% of B, 8 g RediSep® basic Al_2O_3 column, run length = 25 column volumes (CV), eluted at 0% B, 3-5 CV) to yield ligand **9** (178 mg, 369 µmol, 81%) as a light brown oil.

*Note: It is more convenient to dissolve the dark brown solids of **11** in anhydrous acetonitrile for the transfer to the reaction vessel.

TLC: R_f = 0.23 (dichloromethane (95%)/methanol (3.5%)/triethyl amine (1.5%)).

IR (ATR, ṽ/cm^{-1}): 2927 (s), 2853 (s), 1573 (m), 1465 (s), 1367 (s), 1118 (m), 1088 (s), 1053 (m), 1006 (s), 945 (m), 832 (m), 629 (m).

^{1}H-NMR (500 MHz, 298 K, $CDCl_3$, δ/ppm): 8.55 (d, *J* = 2.4 Hz, 2H), 7.70 (dd, *J* = 8.4, 2.4 Hz, 2H), 7.44 (d, *J* = 8.4 Hz, 2H), 3.84 (d, *J* = 14.8 Hz, 2H), 3.71 (d, *J* = 14.8 Hz, 2H), 2.65-2.60 (m, 2H), 2.26 (s, 6H), 1.98-1.93 (m, 2H), 1.80-1.73 (m, 2H), 1.31-1.23 (m, 2H), 1.20-1.10 (m, 2H).

^{13}C{^{1}H}-NMR (126 MHz, 298 K, $CDCl_3$, δ/ppm): 160.2, 149.8, 139.0, 124.3, 118.6, 64.7, 59.9, 36.8, 25.9.

A ^{13}C signal was not observed as reported in the case of the *R*,*R*-enantiomer as well.[7]

HRMS (ESI) m/z: $[M + H]^+$ Calcd for $C_{20}H_{27}Br_2N_4^+$ 481.0597; Found 481.0601.

Ligand (*S,S*-mcp)-RS$_2$ (19)

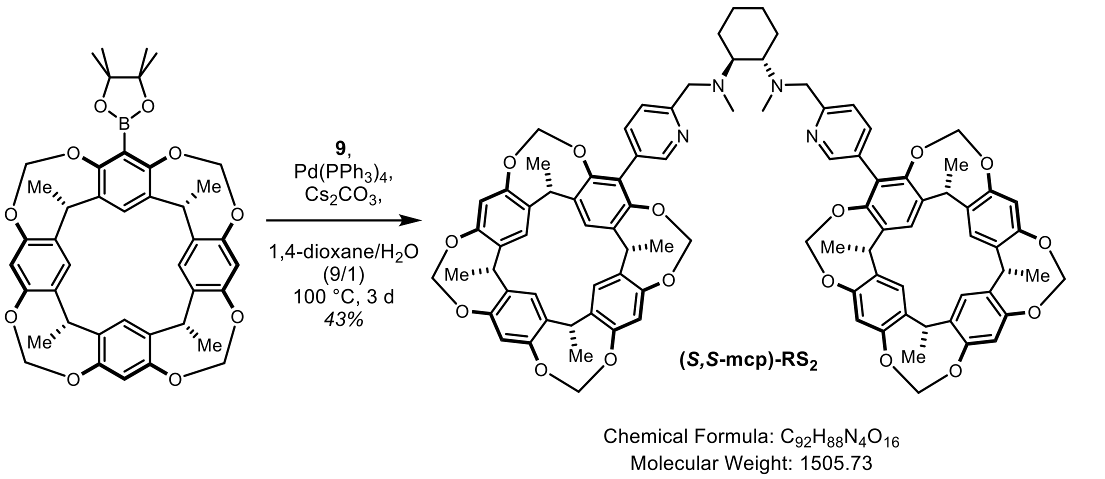

14 **19**

An oven-dried 4 mL vial, equipped with stirring bar and cap with septum, was charged with ground cesium carbonate (45.6 mg, 140 µmol, 4.5 equiv.), ligand (*S,S*-mcp)-diBr (**9**, 15.0 mg, 31.1 µmol, 1.0 equiv.**)**, RS derivative **14** (55.9 mg, 77.8 µmol, 2.5 equiv.) and tetrakis(triphenylphosphine)palladium ($Pd(PPh_3)_4$, orange-red*, 7.19 mg, 6.22 µmol, 0.20 equiv.). After carefully establishing an inert argon atmosphere, a degassed mixture of anhydrous 1,4-dioxane and water (9/1 v/v%, 0.45 mL) was added and argon was bubbled through the reaction mixture for one minute. The punctured cap with septum was replaced with a new one and the vial was sealed with Teflon tape. The reaction mixture was heated to 100 °C (oil bath temp.) and stirred for three days. After allowing to cool down to room temperature, the reaction mixture was filtered through celite and rinsed with dichloromethane (3.0 mL). The filtrate was concentrated *in vacuo* and the resulting crude was purified by flash column chromatography (A: cyclohexane, B: ethyl acetate, gradient: 0-100% of B, 8.0 g RediSep® basic Al_2O_3 column, run length = 158 column volumes (CV), eluted at 25-40% B, 80-110 CV) and in combination with additional purification steps for the triphenylphosphine oxide (TPPO) contaminated fractions** to yield the desired (*S,S*-mcp)-RS$_2$ (**13**, 20.0 mg, 13.3 µmol, 43%) as an off-white solid.

* This particular *Suzuki* cross-coupling reaction to afford **19** worked the best with an orange-red batch of the $Pd(PPh_3)_4$ catalyst whereas the yellow one led to inferior results. The reason for this is yet to be determined. The color of the Pd catalyst will be stated in the following cross-coupling reactions.

** The separation of the side-product TPPO from the desired product **19** on basic aluminum oxide (AlOx) proved to be challenging. Pure fractions of **19** had to be individually determined by NMR to sort out the TPPO containing mixtures. The latter ones were combined and the solvent was removed under reduced pressure. Then in a 12 mL vial, the resulting solids were dissolved in a minimum amount of CH_2Cl_2 (ca. 50 µL), and *n*-hexane (ca. 1.0 mL) was added rapidly and vigorously via a pipette to crash out the desired product. The mixture was centrifuged and the supernatant was removed as much as possible via a pipette and the mixture was dried *in vacuo*. The purity of the mixture was examined by NMR and the process was repeated (in total 4-5 times) until no TPPO remained to yield **19** (5.50 mg of the total 20.0 mg).
It is important to note that several other purification methods to remove TPPO, including separation by gel permeation chromatography (GPC) and $CaCl_2$ precipitation[8] have failed.

^{1}H-NMR (500 MHz, 298 K, $CDCl_3$, δ/ppm): 8.13 (d, J = 2.0 Hz, 2H), 7.64 (d, J = 8.0 Hz, 2H), 7.39 (dd, J = 8.0, 2.0 Hz, 2H), 7.31, (s, 2H), 7.30 (s, 2H), 7.28 (s, 4H), 6.49 (s, 2H), 6.45 (s, 2H), 6.43 (s, 2H), 5.77 (d, J = 7.2 Hz, 4H), 5.45 (d, J = 7.0 Hz, 4H), 5.01-4.95 (m, 8H), 4.44 (d, J = 7.2 Hz, 4H), 4.38 (dd, J = 7.0, 2.0 Hz, 4H), 3.94 (d, J = 15 Hz, 2H), 3.84 (d, J = 15 Hz, 2H), 2.71-2.65 (m, 2H), 2.34 (s, 6H), 2.01-1.96 (m, 2H), 1.81-1.78 (m, 26H), 1.33-1.30 (m, 2H), 1.20-1.16 (m, 2H).

^{13}C{^{1}H}-NMR (126 MHz, 298 K, $CDCl_3$, δ/ppm): 160.4, 154.6, 154.6, 154.5, 152.5, 148.6, 139.7, 139.6, 139.5, 139.5, 139.2, 139.2, 138.3, 122.2, 120.4, 120.2, 119.8, 116.6, 116.2, 99.8, 99.7, 64.8, 60.4, 37.1, 31.1, 30.8, 26.2, 26.0, 16.2, 16.0.

The ^{1}H-NMR data are in agreement with the reported values.[4]

Catalyst Mn(*S,S*-mcp)-RS₂ (7)

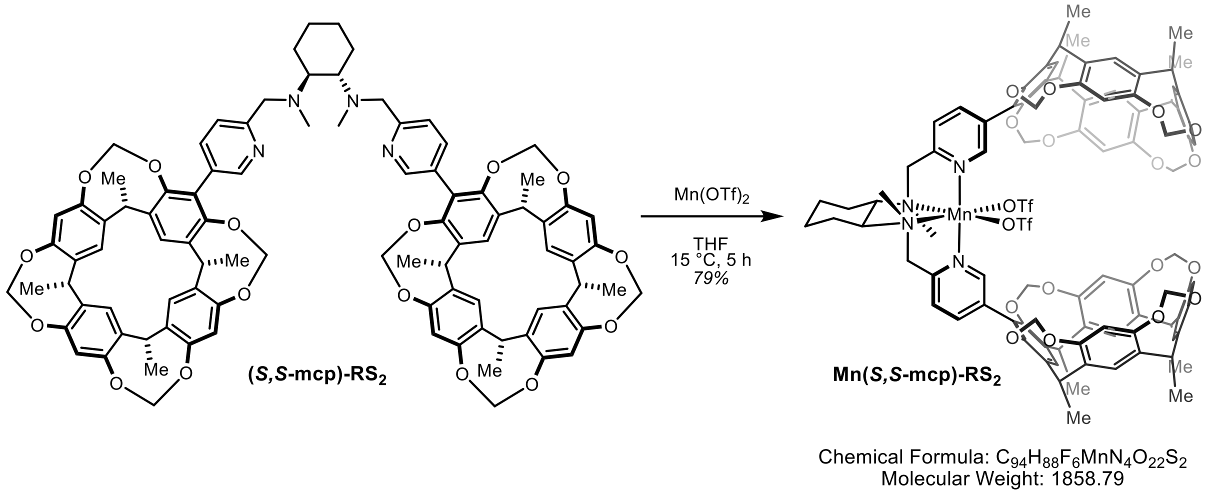

19 **7**

The final step, complexation of the Mn(II)-ion, was carried out according to the reported procedure.[4] Ligand **19** (21.5 mg, 14.3 µmol, 1.0 equiv.) and manganese bis(trifluoromethanesulfonate) (95%, 5.84 mg, 15.7 µmol, 1.1 equiv.) were used to construct the desired supramolecular catalyst Mn(*S,S*-mcp)-RS_2 (**7**, 21.0 mg, 11.3 µmol, 79%, off-white solid). Mass spectrometry analysis was carried out to evaluate the formation of the catalyst **7** prior to its application in C−H oxidation reactions. Analytical data including the crystal structure can be found in our previous work.[4]

2.2 Synthesis of Mn(*S,S*-pdp)-RS$_2$ (4)

Ligand (2*S*,2'*S*)-1,1'-bis((5-bromopyridin-2-yl)methyl)-2,2'-bipyrrolidine = (*S,S*-pdp)-diBr (8)

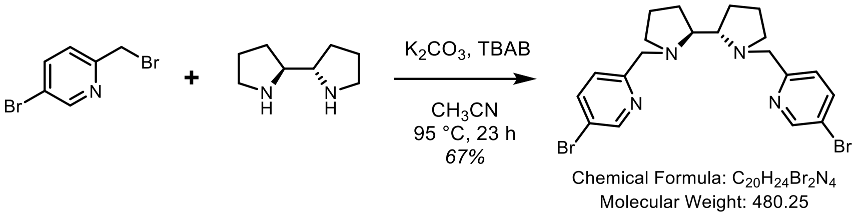

11 **12** **8**

The synthesis of ligand (*S,S*-pdp)-diBr (**8**) was carried out following the procedure for the synthesis of (*S,S*-mcp)-diBr (**9**, *vide supra*). Compound **11** (285 mg, 1.14 mmol, 2.5 equiv.) and (2*S*,2'*S*)-2,2'-bipyrrolidine (**12**, 63.7 mg, 455 µmol, 1.0 equiv.) and the flash chromatography conditions (A: cyclohexane, B: ethyl acetate, gradient: 0-100% of B, 24 g RediSep® basic Al_2O_3 column, run length = 20 column volumes (CV), eluted at 5.0% B, 11-14 CV) were used to synthesize **8** (147 mg, 306 µmol, 67%) as a light brown sticky paste.

TLC: R_f = 0.23 (dichloromethane (95%)/methanol (3.5%)/triethyl amine (1.5%)).

IR (ATR, ṽ/cm^{-1}): 2962 (s), 2872 (m), 2802 (s), 1574 (m), 1466 (s), 1367 (s), 1209 (m), 1116 (s), 1089 (s), 1006 (s), 927 (w), 827 (m), 630 (w).

^{1}H-NMR (500 MHz, 298 K, $CDCl_3$, δ/ppm): 8.54 (d, *J* = 2.4 Hz, 2H), 7.70 (dd, *J* = 8.3, 2.4 Hz, 2H), 7.29 (d, *J* = 8.3 Hz, 2H), 4.15 (d, *J* = 14.8 Hz, 2H), 3.48 (d, *J* = 14.8 Hz, 2H), 3.00-2.96 (m, 2H), 2.79-2.75 (m, 2H), 2.25-2.20 (m, 2H), 1.86-1.79 (m, 2H), 1.76-1.68 (m, 6H).

^{13}C{^{1}H}-NMR (126 MHz, 298 K, $CDCl_3$, δ/ppm): 159.3, 149.9, 139.0, 124.0, 118.6, 66.0, 60.8, 55.5, 26.5, 23.8.

HRMS (ESI) m/z: [M + H]$^+$ Calcd for $C_{20}H_{25}Br_2N_4^+$ 479.0440; Found 479.0438.

Ligand (*S,S*-pdp)-RS$_2$ (16)

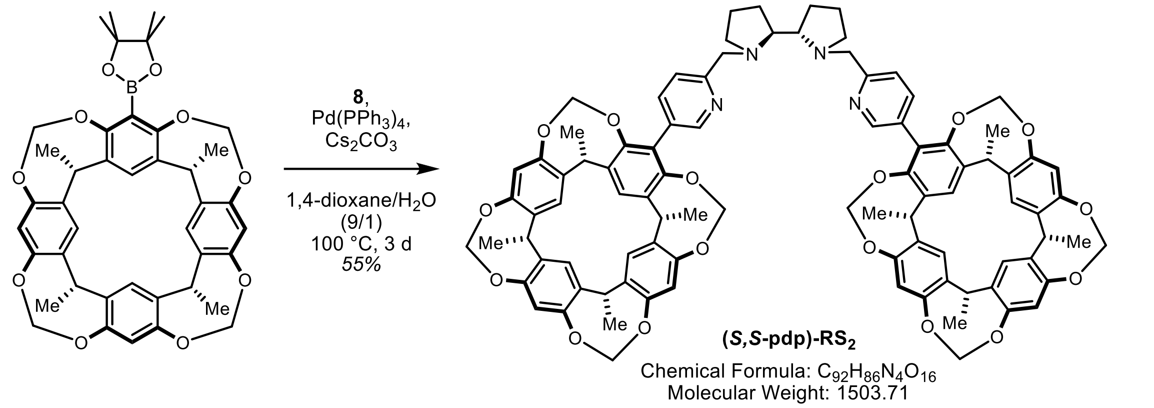

14 **16**

Ligand (*S,S*-pdp)-RS$_2$ (**16**) was synthesized following the procedure for the synthesis of ligand (*S,S*-mcp)-RS$_2$ (**19**) including the additional *n*-hexane purification steps described in the notes (*vide supra*). For this, cesium carbonate (45.8 mg, 141 µmol, 4.5 equiv.), ligand (*S,S*-pdp)-diBr (**8**, 15.0 mg, 31.2 µmol, 1.0 equiv.), RS derivative **14** (56.1 mg, 78.1 µmol, 2.5 equiv.), Pd(PPh$_3$)$_4$ (yellow, 7.22 mg, 6.25 µmol, 0.20 equiv.) and degassed mixture of anhydrous 1,4-dioxane and water (9/1 v/v%, 0.45 mL) were used to give **16** (26.0 mg, 17.3 µmol, 55%) as an off-white solid. The conditions for the purification by flash column chromatography are as follows: solvent A: cyclohexane, B: ethyl acetate, gradient: 0-100% of B, 8.0 g RediSep® basic Al$_2$O$_3$ column, run length = 142 column volumes (CV), eluted at 25-100% B, 100-140 CV.

TLC: R_f = 0.14 (dichloromethane (95%)/methanol (3.5%)/triethyl amine (1.5%)) [CAM].

Mp: 255-262 °C.

IR (ATR, ṽ/cm^{-1}): 2970 (w), 2934 (w), 1580 (w), 1490 (s), 1459 (m), 1283 (m), 1181 (m), 1166 (m), 1097 (s), 1023 (s), 981 (s), 947 (s), 736 (s).

^{1}H-NMR (500 MHz, 298 K, CDCl$_3$, δ/ppm): 8.16 (d, *J* = 2.1 Hz, 2H), 7.45 (d, *J* = 8.0 Hz, 2H), 7.39 (dd, *J* = 8.0, 2.1 Hz, 2H), 7.31 (s, 2H), 7.30 (s, 2H), 7.29 (s, 4H), 6.50 (s, 2H), 6.45 (s, 2H), 6.44 (s, 2H), 5.77 (d, *J* = 7.2 Hz, 4H), 5.45 (d, *J* = 7.2 Hz, 4H), 5.02-4.96 (m, 8H), 4.45 (dd, *J* = 7.2, 1.5 Hz, 4H), 4.38 (t, *J* = 7.2 Hz, 4H), 4.18 (d, *J* = 14 Hz, 2H), 3.54 (d, *J* = 14 Hz, 2H), 3.09-3.05 (m, 2H), 2.82-2.78 (m, 2H), 2.29-2.24 (m, 2H), 1.83-1.77 (m, 28H), 1.76-1.72 (m, 4H).

^{13}C{^{1}H}-NMR (126 MHz, 298 K, CDCl$_3$, δ/ppm): 159.4, 154.6, 154.6, 154.5, 152.5, 148.8, 139.7, 139.5, 139.20, 138.3, 128.5, 126.5, 122.3, 120.4, 120.2, 119.8, 116.6, 116.2, 99.8, 99.8, 65.6, 61.1, 55.7, 31.1, 30.8, 26.0, 23.8, 16.2, 16.2, 16.0.

HRMS (ESI) m/z: [M + H]$^+$ Calcd for $C_{92}H_{87}N_4O_{16}^+$ 1503.6112; Found 1503.6126.

Catalyst Mn(*S,S*-pdp)-RS$_2$ (4)

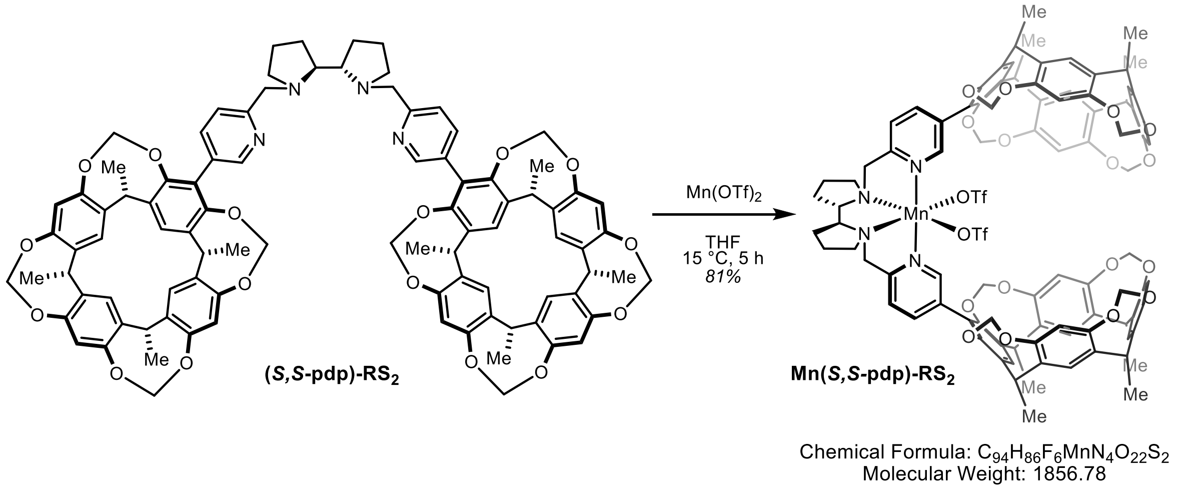

16 **4**

The supramolecular C(sp^3)–H oxidation catalyst Mn(*S,S*-pdp)-RS$_2$ (**4**) was synthesized according to the literature procedure for Mn(*S,S*-mcp)-RS$_2$ (**7**).[4] Ligand **16** (13.3 mg, 8.84 µmol, 1.0 equiv.) and manganese bis(trifluoromethanesulfonate) (95%, 3.62 mg, 9.73 µmol, 1.1 equiv.) were used to construct the desired Mn(*S,S*-pdp)-RS$_2$ (**4**, 13.2 mg, 7.13 µmol, 81%) as an off-white solid.

Mp: Decomposition started at 300 °C.

IR (ATR, ṽ/cm^{-1}): 3470 (w, br), 2968 (w), 2927 (w), 1580 (w), 1492 (m), 1460 (m), 1305 (m), 1283 (s), 1263 (s), 1235 (s), 1213 (m), 1166 (s), 1097 (s), 1023 (s), 981 (s), 946 (s), 734 (s), 637 (s).

HRMS (ESI) m/z: [M - 2OTf$^-$]$^{2+}$ Calcd for $C_{92}H_{86}MnN_4O_{16}^{2+}$ 778.7704; Found 778.7718.

2.3 Synthesis of Mn(*S,S*-pdp)-CX_2 (5)

Calix[4]arene-25,26,27,28-tetrol (SI-2)

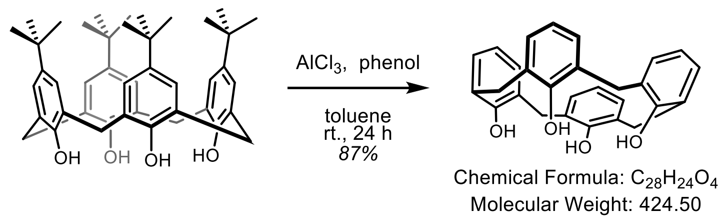

SI-2

The synthesis of calix[4]arene-25,26,27,28-tetrol (**SI-2**)was carried out following a slightly modified literature procedure.[9] A heat-gun dried round bottom flask, under an argon atmosphere equipped with stirring bar, was charged with aluminum chloride (16.4 g, 123 mmol, 8.0 equiv.) and anhydrous toluene (80 mL). Then, commercially available 4-*tert*-butylcalix[4]arene (10.0 g, 15.4 mmol, 1.0 equiv.) and phenol (14.5 g, 154 mmol, 10 equiv.) were added. The resulting reaction mixture was stirred at room temperature for 24 h. Upon full conversion of the CX starting material, the mixture was cooled in an ice-water bath, and an aqueous solution of hydrochloric acid (0.2 M, 126 mL) was added under vigorous stirring, and the mixture was left to stir for 30 min. Stirring was discontinued and the mixture was left to stand overnight. The organic layer was separated, and the aqueous layer was extracted with dichloromethane (2 × 120 mL), and the combined organic layers were washed water, dried over Na_2SO_4 and filtered. Solvent was removed under reduced pressure and methanol (126 mL) was added to the resulting oily crude. The crude mixture was left to stand for 3 days and the precipitates formed were collected by filtration, washed with methanol (2 × 20 mL) and dried *in vacuo* to give **SI-2** (5.66 g, 13.3 mmol, 87%) as an off-white solid. **SI-2** was used for the subsequent reaction without further purification.

^{1}H-NMR (500 MHz, 298 K, $CDCl_3$, δ/ppm): 10.2 (s, 4H), 7.06 (d, *J* = 7.6 Hz, 8H), 6.74 (t, *J* = 7.6 Hz, 4H), 4.28 (broad s, 4H), 3.55 (broad s, 4H).

^{13}C{^{1}H}-NMR (126 MHz, 298 K, $CDCl_3$, δ/ppm): 148.9, 129.1, 128.4, 122.4, 31.9.

The spectroscopic data match the reported values.[10]

25,26,27,28-Tetrapropoxy-calix[4]arene (SI-3)

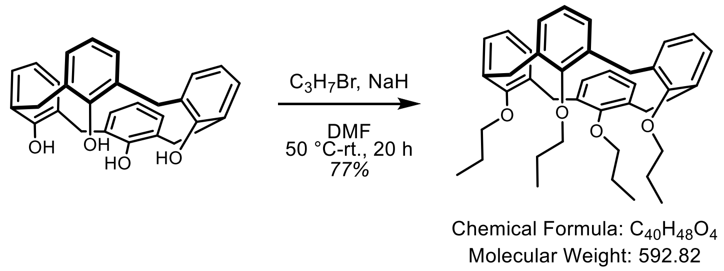

SI-2 **SI-3**

25,26,27,28-Tetrapropoxy-calix[4]arene (**SI-3**) was synthesized following a slightly adapted literature procedure.[11] A heat-gun dried round bottom flask, under an argon atmosphere equipped with stirring bar, was charged with sodium hydride (60% dispersion in mineral oil, 6.03 g, 151 mmol, 16 equiv.) and anhydrous DMF (110 mL). **SI-2** (4.00 g, 9.42 mmol, 1.0 equiv.) was added in four portions and the resulting reaction mixture was heated to 50 °C (oil bath temp.) and stirred for 30 min. After allowing to cool down to room temperature, 1-bromopropane (13.7 mL, 151 mmol, 16 equiv.) was added slowly and the reaction mixture was stirred at rt. for 20 h. Upon full consumption of **SI-2**, ice-water (120 mL) was added, and the mixture was extracted with dichloromethane (3 × 100 mL). The combined organic layers were washed with water (3 × 100 mL), followed by a saturated aqueous ammonium chloride solution (100 mL) and brine (100 mL). After drying over Na_2SO_4 and filtration, solvent was removed

in vacuo to give a yellow crude liquid. Methanol (16 mL) was added, and the crude mixture was left to stand overnight, and the formed precipitates were collected by filtration, washed with additional methanol (3 × 16 mL) and dried under reduced pressure. The crude was purified by flash column chromatography (A: cyclohexane, B: dichloromethane, gradient: 0-100% of B, 80 g RediSep® Silica column, run length = 18 column volumes (CV), eluted at 5-15% B, 7-13 CV) to yield **SI-3** (4.27 g, 7.21 mmol, 77%) as an off-white solid.

^{1}H-NMR (500 MHz, 298 K, $CDCl_3$, δ/ppm): 6.62-6.55 (m, 12H), 4.46 (d, *J* = 13.4 Hz, 4H), 3.85 (t, *J* = 7.4 Hz, 8H), 3.15 (d, *J* = 13.4 Hz, 4H), 1.96-1.89 (m, 8H), 1.00 (t, *J* = 7.5 Hz, 12H).

^{13}C{^{1}H}-NMR (126 MHz, 298 K, $CDCl_3$, δ/ppm): 156.7, 135.3, 128.3, 122.0, 76.8, 31.1, 23.4, 10.5.

The spectroscopic data are in agreement with the literature values.[12]

4,4,5,5-Tetramethyl-2-(1^2,3^2,5^2,7^2-tetrapropoxy-1,3,5,7(1,3)-tetrabenzenacyclooctaphane-1^5-yl)-1,3,2-dioxaborolane (15)

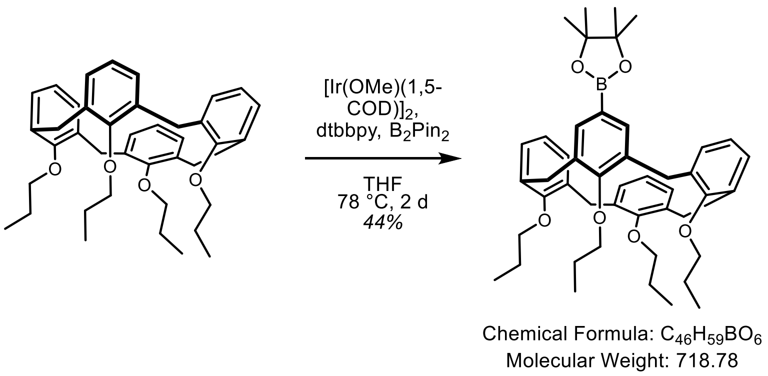

SI-3 **15**

The synthesis of CX derivative **15** was inspired by literature procedures.[13,14] An oven-dried pressure tube, under an argon atmosphere equipped with stirring bar, was charged with ligand 4,4′-di-*tert*-butyl-2,2′-dipyridyl (dtbbpy, 72.4 mg, 0.270 mmol, 0.08 equiv., 8.0 mol%), bis(pinacolato)diboron (856 mg, 3.37 mmol, 1.0 equiv.), $[Ir(OMe)(1,5\text{-}COD)]_2$ (72.4 mg, 0.135 mmol, 0.04 equiv., 4.0 mol%) and degassed THF (5.0 mL). The reaction mixture was stirred for 10 min until a dark red color appeared. Then, **SI-3** (2.00 g, 3.37 mmol, 1.0 equiv.) was added and the reaction mixture was degassed for 1 minute by bubbling argon through it. The vessel was closed and sealed with Teflon tape and the mixture was heated to 78 °C (oil bath temp.) and stirred for 2 days. After allowing to cool down to room temperature the solvent was removed under reduced pressure and the resulting crude was purified by flash column chromatography (A: cyclohexane, B: dichloromethane, gradient: 0-100% of B, 40 g RediSep® Silica column, run length = 23 column volumes (CV), eluted at 30-50% B, 10-15 CV) to yield **15** (1.06 g, 1.48 mmol, 44%) as an light tan solid.

^{1}H-NMR (500 MHz, 298 K, $CDCl_3$, δ/ppm): 7.50 (s, 2H), 7.03 (d, *J* = 7.5 Hz, 2H), 6.85 (t, *J* = 7.5 Hz, 1H,), 6.26 (t, *J* = 7.5 Hz, 2H), 6.20 (dd, *J* = 7.5, 1.6 Hz, 2H), 6.16 (dd, *J* = 7.5, 1.6 Hz, 2H), 4.46-4.41 (m, 4H), 4.04-3.98 (m, 4H), 3.73-3.65 (m, 4H), 3.19 (d, *J* = 13.5 Hz, 2H), 3.14 (d, *J* = 13.5 Hz, 2H), 2.00-1.84 (m, 8H), 1.37 (s, 12H), 1.08 (t, *J* = 7.4 Hz, 6H), 0.91-0.86 (m, 6H).

^{13}C{^{1}H}-NMR (126 MHz, 298 K, $CDCl_3$, δ/ppm): 161.1, 158.0, 155.5, 137.0, 136.4, 135.7, 133.6, 133.6, 128.9, 127.8, 127.6, 122.2, 121.9, 83.6, 77.0, 76.6, 76.6, 31.1, 31.0, 25.1, 23.6, 23.2, 23.2, 10.9, 10.1, 10.0.

The spectroscopic data is in accordance with the literature values.[15]

Ligand (*S,S*-pdp)-CX₂ (17)

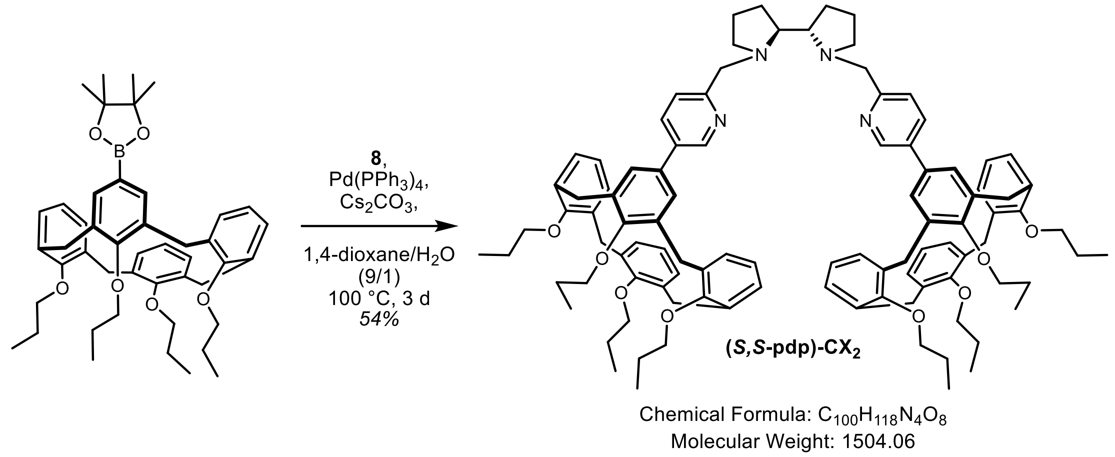

15 **17**

Ligand (*S,S*-pdp)-CX_2 (**17**) was synthesized following the procedure for the synthesis of ligand (*S,S*-mcp)-RS_2 (**19**) excluding the additional *n*-hexane purification steps. For this, cesium carbonate (30.5 mg, 93.6 µmol, 4.5 equiv.), ligand (*S,S*-pdp)-diBr (**8**, 10.0 mg, 20.8 µmol, 1.0 equiv.), CX derivative **15** (37.4 mg, 52.0 µmol, 2.5 equiv.), $Pd(PPh_3)_4$ (yellow, 4.81 mg, 4.16 µmol, 0.20 equiv.) and degassed mixture of anhydrous 1,4-dioxane and water (9/1 v/v%, 0.30 mL) were used to give **17** (17.0 mg, 11.3 µmol, 54%) as a light-yellow solid. The conditions for the purification by flash column chromatography are as follows: solvent A: cyclohexane, B: ethyl acetate, gradient: 0-100% of B, 8.0 g RediSep® basic Al_2O_3 column, run length = 72 column volumes (CV), eluted at 8-10% B, 25-45 CV.

TLC: R_f = 0.27 (dichloromethane (95%)/methanol (3.5%)/triethyl amine (1.5%)) [CAM].

Mp: 150-157 °C.

IR (ATR, ṽ/cm^{-1}): 2960 (s), 2925 (s), 2874 (s), 1587 (w), 1459 (s), 1384 (m), 1289 (m), 1245 (s), 1208 (s), 1194 (s), 1156 (m), 1088 (s), 1006 (s), 967 (s) 760 (s).

^{1}H-NMR (500 MHz, 298 K, $CDCl_3$, δ/ppm): 8.27 (d, *J* = 2.0 Hz, 2H), 7.36 (dd, *J* = 8.1, 2.0 Hz, 2H), 7.31 (d, *J* = 8.1 Hz, 2H), 6.91-6.86 (m, 8H), t (6.76, *J* = 7.5 Hz, 4H), 6.51-6.49 (m, 4H), 6.25 (t, *J* = 8.0 Hz, 4H), 6.04 (t, *J* = 7.5 Hz, 2H), 4.50 (d, *J* = 13.4 Hz, 4H), 4.45 (d, *J* = 13.4 Hz, 4H), 4.16 (d, *J* = 14.2 Hz, 2H), 4.00-3.91 (m, 8H), 3.80 (t, *J* = 7.0 Hz, 4H), 3.74 (t, *J* = 7.0 Hz, 4H), 3.46 (d, *J* = 14.2 Hz, 2H), 3.20 (d, *J* = 13.4 Hz, 4H), 3.14 (d, *J* = 13.4 Hz, 4H), 3.00-2.97 (m. 2H), 2.83-2.79 (m, 2H), 2.22 (q, *J* = 8.6 Hz, 2H), 1.99-1.87 (m, 16H), 1.84-1.76 (m, 4H), 1.73-1.66 (m, 4H), 1.09-1.04 (m, 12H), 0.94 (t, *J* = 7.5 Hz, 12H).

^{13}C{^{1}H}-NMR (126 MHz, 298 K, $CDCl_3$, δ/ppm): 158.0, 157.5, 156.2, 155.9, 147.3, 136.4, 136.0, 136.0, 135.0, 134.9, 134.5, 134.2, 131.4, 128.9, 128.7, 127.7, 126.5, 122.3, 122.2, 122.1, 122.0, 77.1, 76.7, 65.4, 60.9, 55.5, 31.2, 31.1, 25.9, 23.6, 23.6, 23.5, 23.3, 10.8, 10.2.

HRMS (ESI) m/z: $[M + H]^+$ Calcd for $C_{100}H_{119}N_4O_8^+$ 1503.9022; Found 1503.8997.

Catalyst Mn(*S,S*-pdp)-CX$_2$ (5)

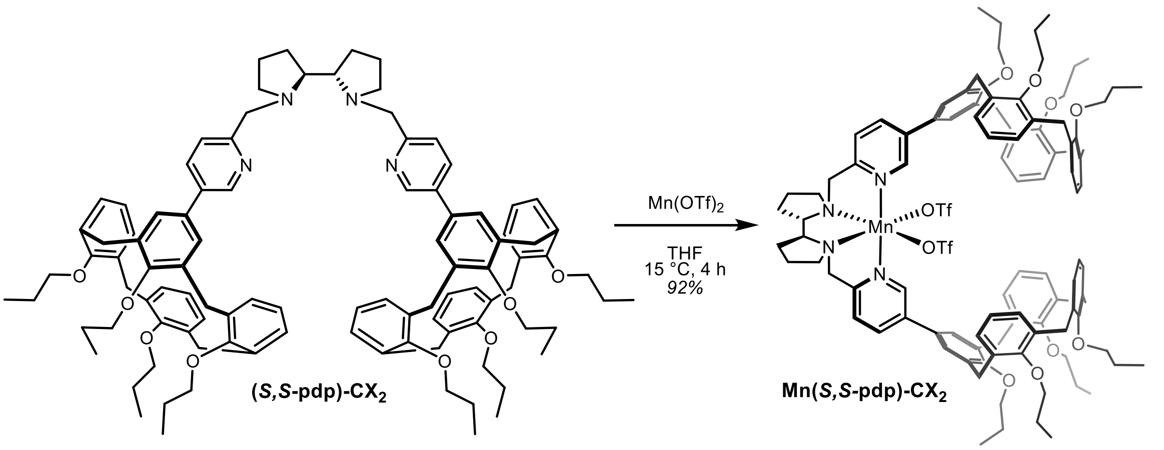

Chemical Formula: $C_{102}H_{118}F_6MnN_4O_{14}S_2$
Molecular Weight: 1857.13

17 **5**

In a glovebox, an oven-dried 4 mL vial was charged with ligand **17** (17.0 mg, 11.3 µmol, 1.0 equiv.), manganese bis(trifluoromethanesulfonate) (95%, 4.28 mg, 11.5 µmol, 1.0 equiv.) and anhydrous THF (0.38 mL) and the resulting reaction mixture was stirred at 15 °C for 4 hours. Then, anhydrous *n*-hexane (2 × 1.5 mL) was added rapidly and vigorously* by a pipette to precipitate the catalyst. The reaction mixture was transferred out of the glove box, the vial was centrifuged (a), and the supernatant was carefully removed as much as possible by a pipette (b). The solids were dried under reduced pressure (c), and the vial was put back into the glovebox. Anhydrous *n*-hexane (3.0 mL) was added, and the vial was shaken vigorously and subsequently the steps (a-c) performed outside of the glovebox were repeated to give the desired supramolecular catalyst Mn(*S,S*-pdp)-CX$_2$ (**5**, 19.3 mg, 10.4 µmol, 92%) as an off-white solid.

* When the addition speed was too slow or/and not vigorously enough, the catalyst did not precipitate.

Mp: 230-237 °C.

IR (ATR, ṽ/cm^{-1}): 2961 (m), 2933 (m), 2875 (m), 2361 (w), 1586 (w), 1460 (s), 1384 (w), 1241 (s), 1211 (s), 1161 (s), 1088 (s), 1031 (s), 1006 (s), 966 (s), 895 (w), 831 (w), 759 (s), 638 (s).

HRMS (ESI) m/z: [M - 2OTf$^-$]$^{2+}$ Calcd for $C_{100}H_{118}MnN_4O_8^{2+}$ 778.9160; Found 778.9157.

2.4 Synthesis of Mn(*S,S*-mcp)-CX$_2$ (6)

Ligand (*S,S*-mcp)-CX$_2$ (18)

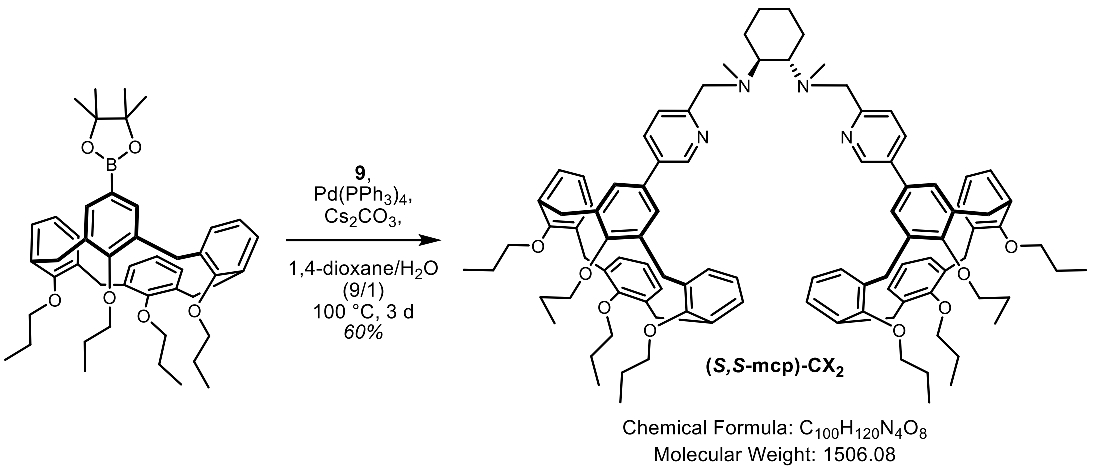

15 **18**

Ligand (*S,S*-mcp)-CX$_2$ (**18**) was synthesized following the procedure for the synthesis of ligand (*S,S*-mcp)-RS$_2$ (**19**) excluding the additional *n*-hexane purification steps. For this, cesium carbonate (30.5 mg, 93.6 µmol, 4.5 equiv.), ligand (*S,S*-mcp)-diBr (**9**, 10.0 mg, 20.8 µmol, 1.0 equiv.), CX derivative **15** (37.4 mg, 52.0 µmol, 2.5 equiv.), Pd(PPh$_3$)$_4$ (yellow, 4.81 mg, 4.16 µmol, 0.20 equiv.) and degassed solvent mixture of anhydrous 1,4-dioxane and water (9/1 v/v%, 0.30 mL) were used to give **18** (18.9 mg, 12.5 µmol, 60%) as a light-yellow solid. The conditions for the purification by flash column chromatography are as follows: solvent A: cyclohexane, B: ethyl acetate, gradient: 0-100% of B, 8.0 g RediSep® basic Al$_2$O$_3$ column, run length = 68 column volumes (CV), eluted at 8-10% B, 30-45 CV.

To showcase the practical utility of the convergent synthesis approach, a tenfold scaled-up reaction was carried out in a 12 mL vial with ligand (*S,S*-mcp)-diBr (**9**, 100 mg, 207 µmol, 1.0 equiv.), CX derivative **15** (379 mg, 527 µmol, 2.5 equiv.), Pd(PPh$_3$)$_4$ (yellow, 48.9 mg, 42.3 µmol, 0.20 equiv.) and degassed mixture of anhydrous 1,4-dioxane and water (9/1 v/v%, 3.0 mL) to afford **18** (185 mg, 123 µmol, 59% (NMR yield = 72%)) as a light-yellow solid.

TLC: R_f = 0.27 (dichloromethane (95%)/methanol (3.5%)/triethyl amine (1.5%)) [CAM].

Mp: 148-154 °C.

IR (ATR, ṽ/cm^{-1}): 2960 (m), 2927 (s), 2874 (m), 1587 (w), 1456 (s), 1384 (m), 1289 (m), 1245 (s), 1208 (s), 1193 (s), 1157 (m), 1087 (s), 1006 (s), 966 (s), 842 (m), 759 (s).

^{1}H-NMR (500 MHz, 298 K, CDCl$_3$, δ/ppm): 8.27 (d, *J* = 1.8 Hz, 2H), 7.52 (d, *J* = 8.1 Hz, 2H), 7.40 (dd, *J* = 8.1, 1.8 Hz, 2H), 6.89-6.84 (m, 8H), 6.76-6.72 (m, 4H), 6.54 (d, *J* = 6.7 Hz, 4H), 6.28 (t, *J* = 6.7 Hz, 4H), 6.08 (t, *J* = 7.5 Hz, 2H), 4.50 (d, *J* = 13.4 Hz, 4H), 4.45 (d, *J* = 13.4 Hz, 4H), 3.99-3.80 (m, 16H), 3.75 (t, *J* = 7.1 Hz, 4H), 3.20 (dd, *J* = 13.4, 3.9 Hz, 4H), 3.14 (d, *J* = 13.4 Hz, 4H), 2.65 (d, *J* = 8.5 Hz, 2H), 2.29 (s, 6H), 1.99-1.86 (m, 18H), 1.75 (d, *J* = 7.6 Hz, 2H), 1.34-1.28 (m, 2H), 1.17-1.13 (m, 2H), 1.09-1.03 (m, 12H), 0.95 (t, *J* = 7.5 Hz, 12H).

^{13}C{^{1}H}-NMR (126 MHz, 298 K, CDCl$_3$, δ/ppm): 159.1, 157.4, 156.3, 156.0, 147.1, 136.3, 135.9, 135.1, 134.9, 134.5, 134.3, 131.6, 128.9, 128.7, 127.7, 126.6, 126.5, 122.4, 122.1, 122.0, 77.0, 76.8, 64.6, 60.7, 36.8, 31.2, 31.1, 26.4, 26.0, 23.6, 23.5, 23.3, 10.7, 10.7, 10.2.

HRMS (ESI) m/z: [M + H]$^+$ Calcd for $C_{100}H_{121}N_4O_8^+$ 1505.9179; Found 1505.9162.

Catalyst Mn(*S,S*-mcp)-CX$_2$ (6)

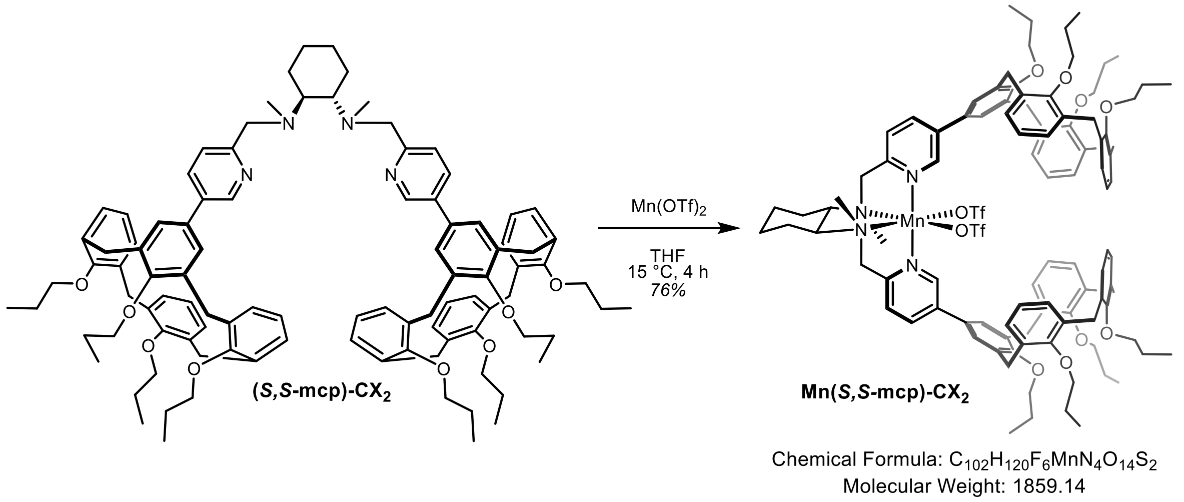

18 **6**

The synthesis of catalyst **6** was carried out as described for Mn(*S,S*-pdp)-CX$_2$ (**5**, *vide supra*). For this, ligand **18** (18.5 mg, 12.3 µmol, 1.0 equiv.), manganese bis(trifluoromethanesulfonate) (95%, 4.79 mg, 12.9 µmol, 1.1 equiv.) and anhydrous THF (0.41 mL) were used to afford desired Mn(*S,S*-mcp)-CX$_2$ (**6**, 17.3 mg, 9.31 µmol, 76%) as an off-white solid.

Mp: 250-257 °C.

IR (ATR, ṽ/cm^{-1}): 2961 (m), 2934 (m), 2875 (m), 2360 (w), 1586 (w), 1457 (s), 1384 (m), 1288 (m), 1240 (s), 1210 (s), 1192 (s), 1162 (s), 1088 (m), 1030 (s), 1006 (s), 966 (s), 760 (m), 638 (s).

HRMS (ESI) m/z: $[M - 2OTf^{-}]^{2+}$ Calcd for $C_{100}H_{120}MnN_4O_8^{2+}$ 779.9238; Found 779.9246.

3. C−H Oxidation Studies

3.1 General Oxidation Procedure

According to a modified literature procedure,[16] the oxidation reactions consist of 1-2 step(s) depending on the separation of the alcohol products on the GC-column. If overlapping product signals occurred, the secondary alcohols were converted to the corresponding ketones via oxidation with 2-iodoxybenzoic acid (IBX). The GC response factors of the oxidation products were calculated as described in the literature.[1,2] Conversion and yield were calculated based on the GC standard biphenyl. Site-selectivity of a certain product was calculated as follows: yield of a particular product (adjusted with the calculated response factor)/total yield of mono-oxidized products (mono-alcohols or/and -ketones).

H_2O_2 oxidation in 2,2,2-trifluoroethanol (TFE): A solution of 2,2-dimethylpropanoic acid (2,2-diMe-PA) in TFE (2.0 M, 200 µL, 407 µmol, 22 equiv., unless specified otherwise), substrate **Sx** (18.5 µmol, 1.0 equiv.) were added to a 2.0 mL vial charged with catalyst **[M]** (1.0 mol%) and stirring bar.* The mixture was cooled in an ice-water bath. Next, a solution of H_2O_2 (50 wt% diluted in TFE, 0.9 M, 20.6 µL, 18.5 µmol, 1.0 equiv., unless otherwise specified) was slowly added over 15 minutes via syringe pump. The reaction mixture was stirred for further 45 minutes. Subsequently, H_2O (1.5 mL) and a solution of biphenyl in ethyl acetate (92.5 mM, 40 µL, 3.70 µmol, 0.20 equiv.) were added. The mixture was extracted with ethyl acetate (2 × 1.5 mL) and the combined organic layers were washed with a saturated solution of $NaHCO_3$ (2.0 mL), followed by H_2O (2.0 mL), dried over Na_2SO_4, filtered through a silica plug and rinsed with ethyl acetate (0.50 mL). The mixture was analyzed via GC and GC-MS.

* In oxidation with CX catalysts **5** and **6**, an additional solution of trifluoromethanesulfonic acid (TfOH) in TFE (23.2 mM, 20 µL, 0.463 µmol, 0.025 equiv., 2.5 mol%) was added before the addition of the substrate.

IBX oxidation: 0.5 mL of the crude mixture (ca. 3.0 mL) was added to a 2 mL vial charged with IBX[17] (3.50 mg, 12.5 µmol) and stirring bar. The vial was capped and sealed with Teflon tape and the mixture was heated to 66 °C (temp. of the aluminum heating block) for 16 hours. After allowing to cool down to room temperature, the mixture was filtered through a silica plug and the vial was rinsed with ethyl acetate (2 × 1.0 mL). GC and GC-MS analyses were conducted as described in detail below.

3.2 Solvent and Carboxylic Acid Screening with CX-catalysts 5 and 6

As the two CX-catalysts, Mn(pdp)-CX_2 (**5**) and Mn(mcp)-CX_2 (**6**), did not exhibit noteworthy site-selectivity change employing the general oxidation conditions (see section 3.1 General Oxidation Procedure) as compared to the parent catalysts **20** and **21**, further solvent and carboxylic acid screenings with substrate octane were carried out. It can be seen in Table S1 that both CX-catalysts' performance decreased in HFIP (entries 3&4) as compared to reactions ran in TFE (entries 1&2). Slightly higher conversion and yield were achieved in NFTBA (entries 5&6) but no significant changes in selectivity were observed.

Table S1. Solvent screening results following the general oxidation procedure with substrate octane, additive 2,2-diMe-PA and TfOH (2.5 mol%).

Solvent Screening					
**Entry**	**Solvent**	**Mn(*)-CX_2**	**Conv. (%)**	**GC Yield (A&K, %)**	**Selectivity C(2/3/4) (%)**
1	TFE	pdp	49	25	41/28/31
2		mcp	54	37	43/27/30
3[a]	HFIP	pdp	18	0	-
4		mcp	23	18	43/28/29
5	NFTBA	pdp	52	35	41/28/31
6		mcp	62	51	42/27/31

1,1,1,3,3,3-hexafluoroisopropanol (HFIP), nonafluoro-*tert*-butyl alcohol (NFTBA), conversion (conv.), alcohol (A), ketone (K), [a] results from two independent experiments.

Unfortunately, no improvement in C(4) selectivity of Mn(mcp)-CX_2 (**6**) was achieved by varying the carboxylic acid additive (A-H, Table S2) either.

Table S2. Screening of carboxylic acid additives following the general oxidation procedure with Mn(mcp)-CX_2 (6) substrate octane, additive TfOH (2.5 mol%) in TFE.

Carboxylic acid Screening in TFE				
**Entry**	**Acid (equiv.)**	**Conv. (%)**	**GC Y. (A&K, %)**	**Selectivity C(2/3/4) (%)**
1	A (22)	64	36	40/28/32
2	B (22)	66	38	40/28/32
3	C (22)	58	31	41/28/31
4	D (22)	54	37	43/27/30
5	E (0.5)	53	30	42/27/31
6	F (0.5)	47	23	41/29/30
7	G (0.5)	48	21	42/28/29
8	H (0.5)	26	-	-

Conversion (conv.), alcohol (A), ketone (K).

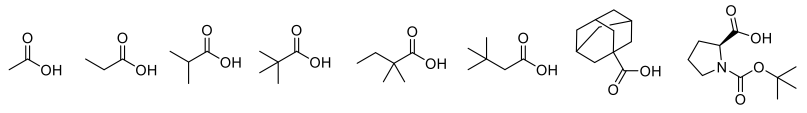

A B C D E F G Boc-L-proline (H)

3.3 Detailed Oxidation Results

<u>Important remark</u>: In our previous work, we observed in addition to the main products (alcohols and ketones) the formation of two more product classes, i.e. epoxide and ester, in the mass balance study conducted with substrate octane.[4] Due to high cost of identification for each individual substrate, these two side products were excluded from the discussion on site selectivity.

<u>Octane (S1)</u>

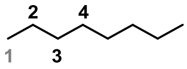

Chemical Formula: C_8H_{18}
Exact Mass: 114.14
Molecular Weight: 114.23

S1

The oxidation reactions were carried out following the general oxidation procedure in TFE with 2,2-DiMe-PA and catalysts **4**, **5**, **14** and **15**, <u>without</u> the 2nd IBX-oxidation step. The <u>achiral</u> GC-column and standard temperature program were used for GC and GC-MS analyses. Comparison of the GC-chromatograms (chroma.) of the crude reaction mixtures obtained from the three pdp-catalysts is shown in Figure S1 and results from the three mcp-catalysts are presented in Figure S3. The detailed identification of the side products epoxides (blue) and carboxylic acid esters (green) via comparison with synthesized reference compounds can be found in our previous work.[4] The RS-induced site-selectivity change can also be observed in the product distribution of the epoxides and especially of the esters in chromatograms B and E as compared to the unsubstituted catalysts **20** (chroma. A) and **21** (chroma. D).

Expansions with the focus on the main oxidation products, alcohols (A) and ketones (K), are presented in Figure S2 and Figure S4, in which the pronounced C(4)-selectivity (sum of A(4) + K(4)) of the RS supramolecular catalysts **4** and **7** (chromatograms B1 and E1, respectively) can be seen unambiguously. On the other hand, the site-selectivity of CX catalysts **5** and **6** (C1 and F1) do not differ significantly from the ones of the parent catalysts **20** and **21** (A and D).

Furthermore, identification of the alcohols and ketones via GC-MS can be found in Figure S5 and Figure S6, respectively

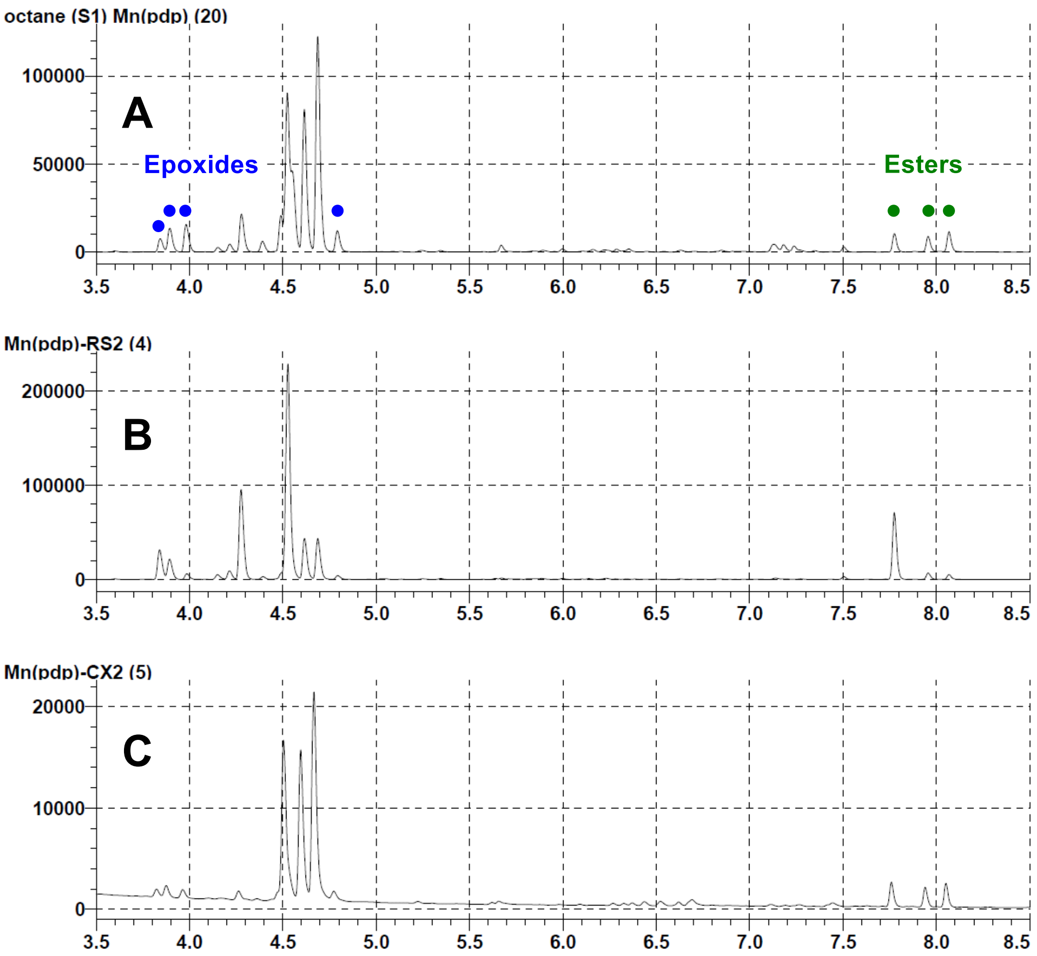

Figure S1. Comparison of pdp-catalysts. Oxidation results of octane (**S1**) carried out in TFE with carboxylic acid 2,2-diMe-PA.

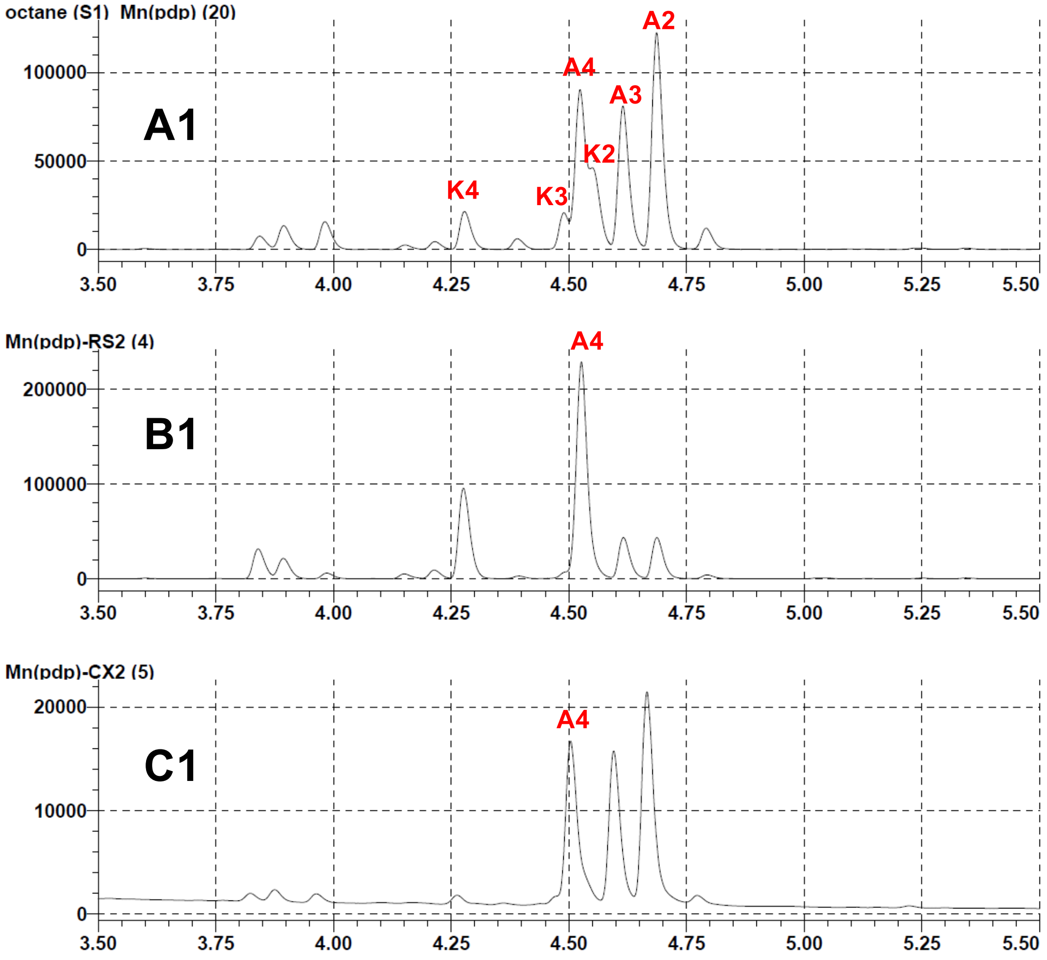

Figure S2. Comparison of pdp-catalysts. Oxidation results of octane (**S1**) carried out in TFE with carboxylic acid 2,2-DiMe-PA. Focus set on the main products alcohol/ketone on carbon x, denominated as Ax/Kx.

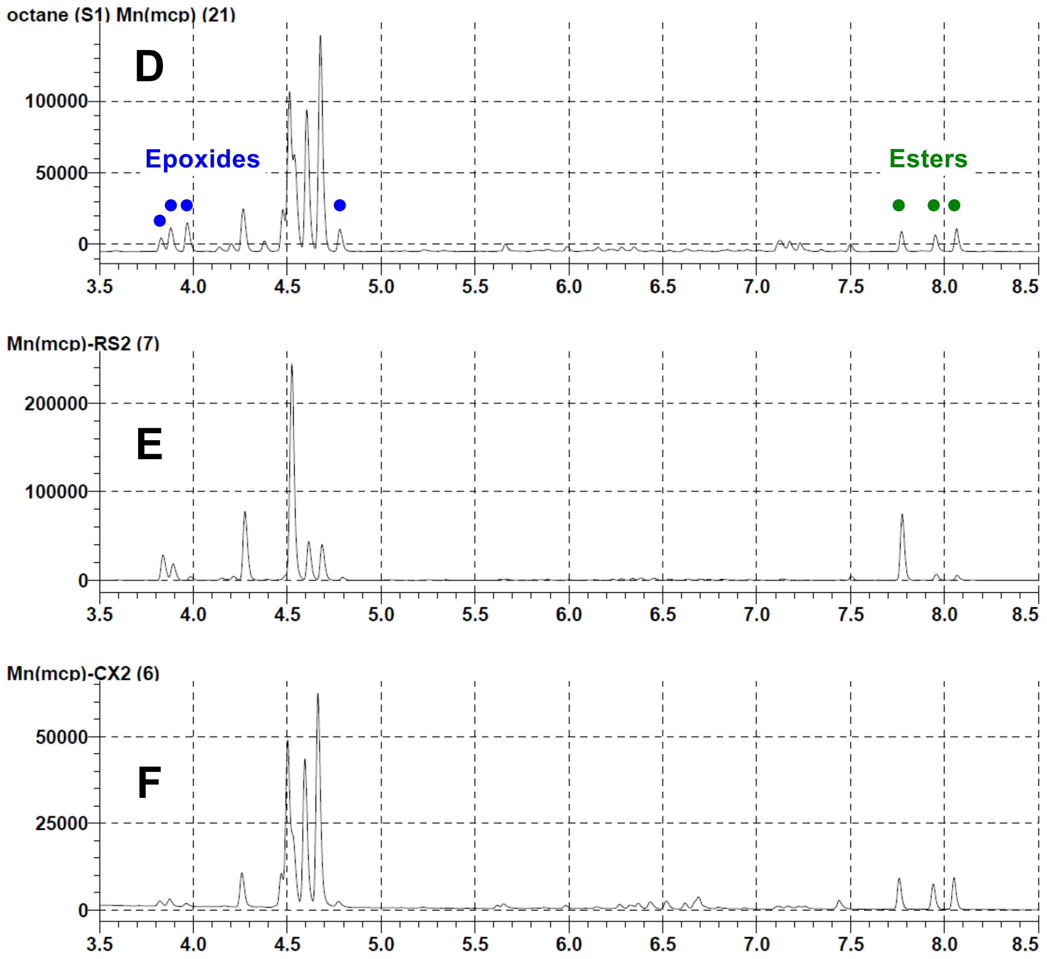

Figure S3. Comparison of mcp-catalysts. Oxidation results of octane (**S1**) carried out in TFE with carboxylic acid 2,2-diMe-PA.

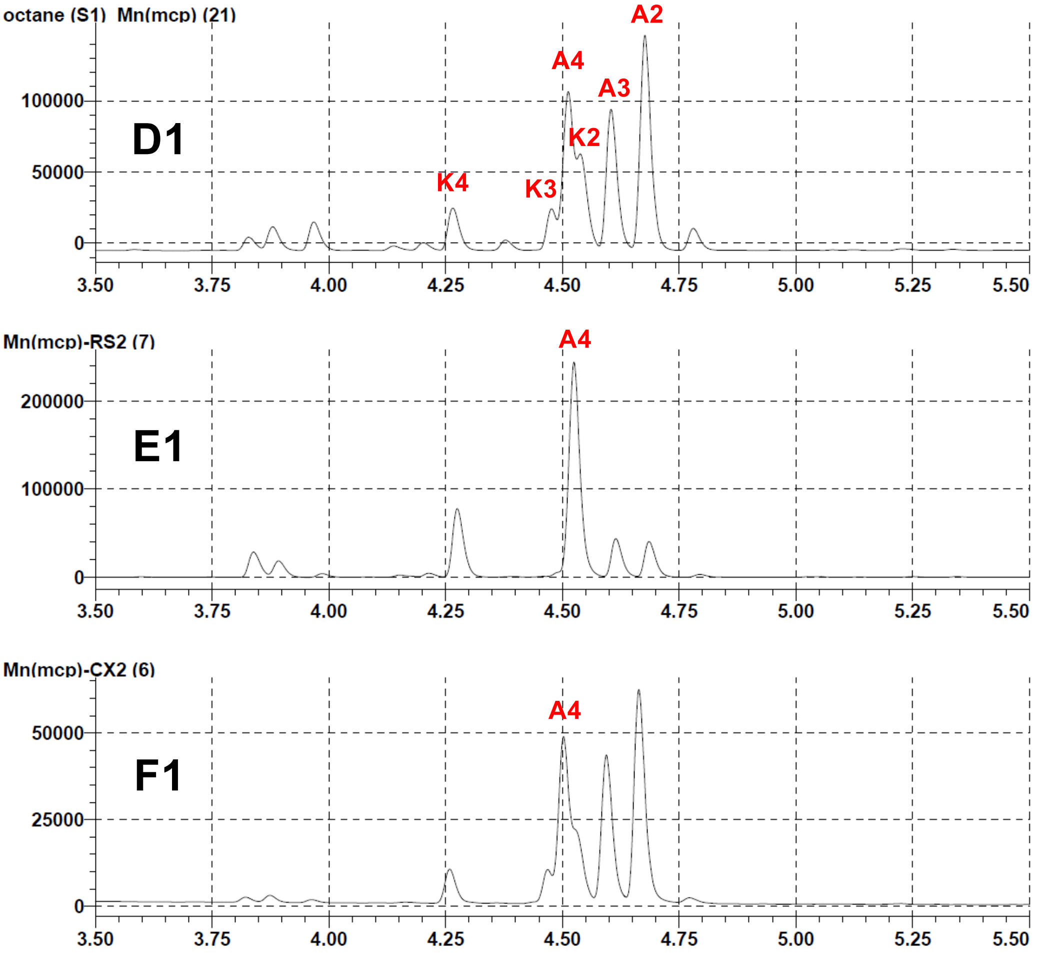

Figure S4. Comparison of mcp-catalysts. Oxidation results of octane (**S1**) carried out in TFE with carboxylic acid 2,2-DiMe-PA. Focus set on the main products alcohol/ketone on carbon x, denominated as Ax/Kx.

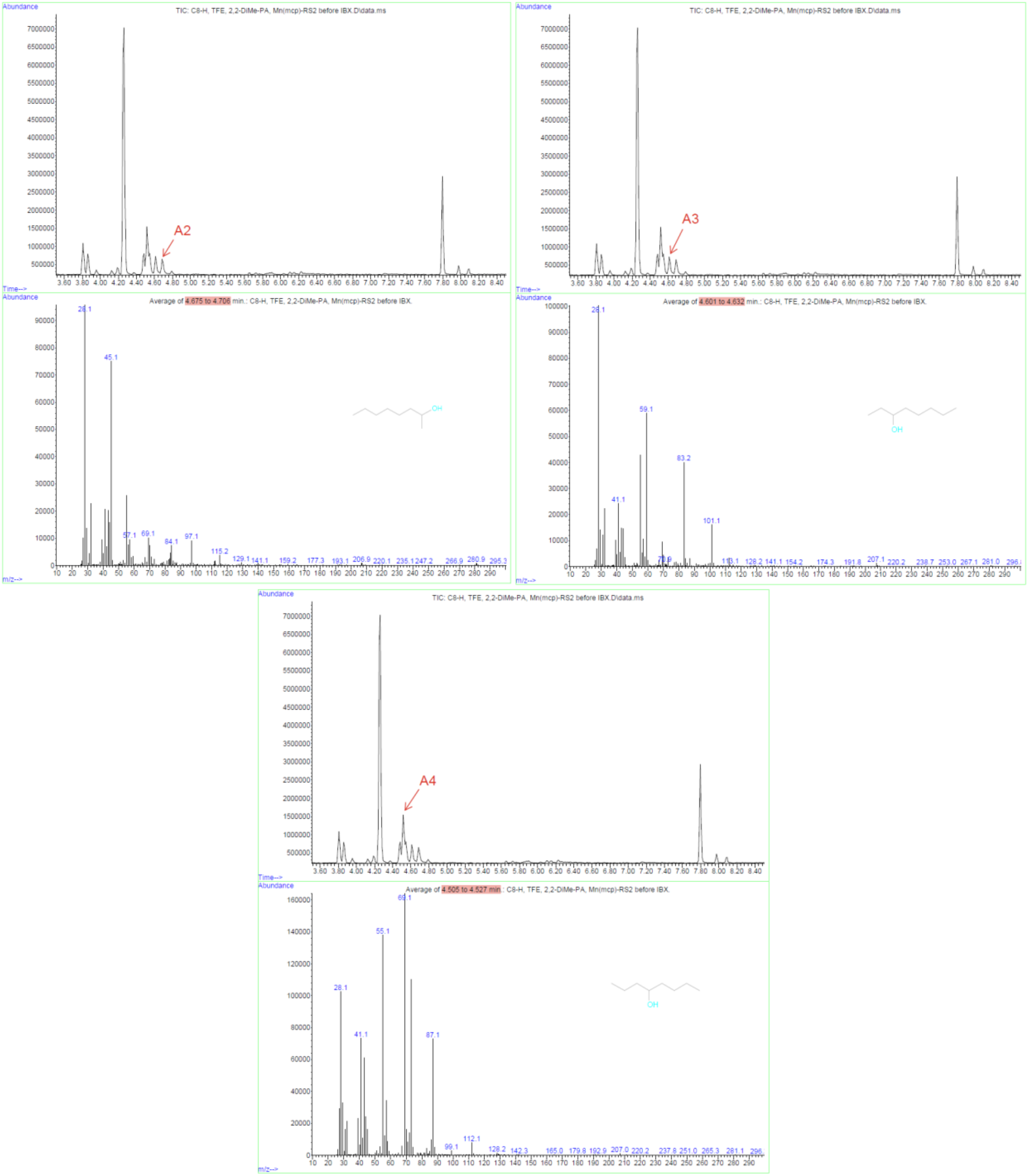

Figure S5. Results from GC-MS analyses of the alcohol products (A2-4) formed in the oxidation of octane (**S1**) in TFE with 2,2-diMe-PA, hydrogen peroxide (2.0 equiv. over 30 min) and catalyst Mn(mcp)-RS_2 (**7**).

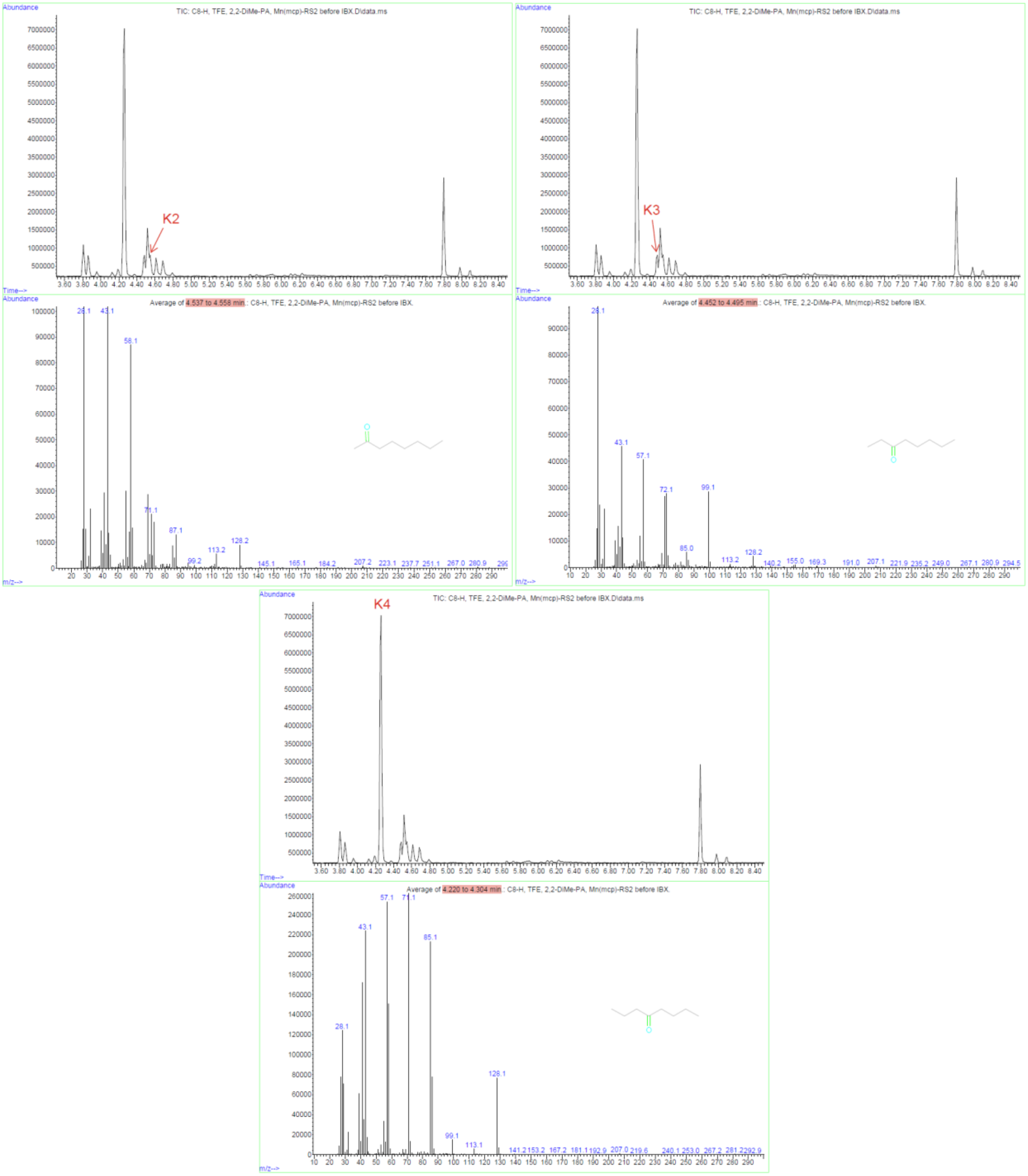

Figure S6. Results from GC-MS analyses of the alcohol products (K2-4) formed in the oxidation of octane (**S1**) in TFE with 2,2-diMe-PA, hydrogen peroxide (2.0 equiv. over 30 min) and catalyst Mn(mcp)-RS_2 (**7**).

2-Methylnonane (S2)

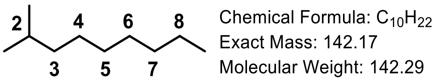

S2

The oxidation reactions were carried out following the general oxidation procedure in TFE with 5.0 equivalents of 2,2-DiMe-PA including the 2nd IBX oxidation step. All products were identified via GC-MS analysis and reported in our previous work.[4] The achiral GC-column and a modified temperature program (60 °C for 3 min, 15 °C/min to 75 °C, 1 °C/min to 85 °C, then 25 °C/min to 250 °C and held for 5 min) were used for GC and GC-MS analyses. Comparison of the GC-chromatograms of the crude reaction mixtures obtained from the three pdp-catalysts is shown in Figure S7, and results of the three mcp-catalysts in Figure S8. The pronounced C(5)-selectivity of the RS supramolecular catalysts **4** and **7** (chromatograms B and E, respectively) can be seen unambiguously. Whereas the site-selectivity of CX catalysts **5** and **6** (C and F) do not differ from the ones of the parent catalysts **20** and **21** (A and D). GC-MS analyses of the products are shown in Figure S9.

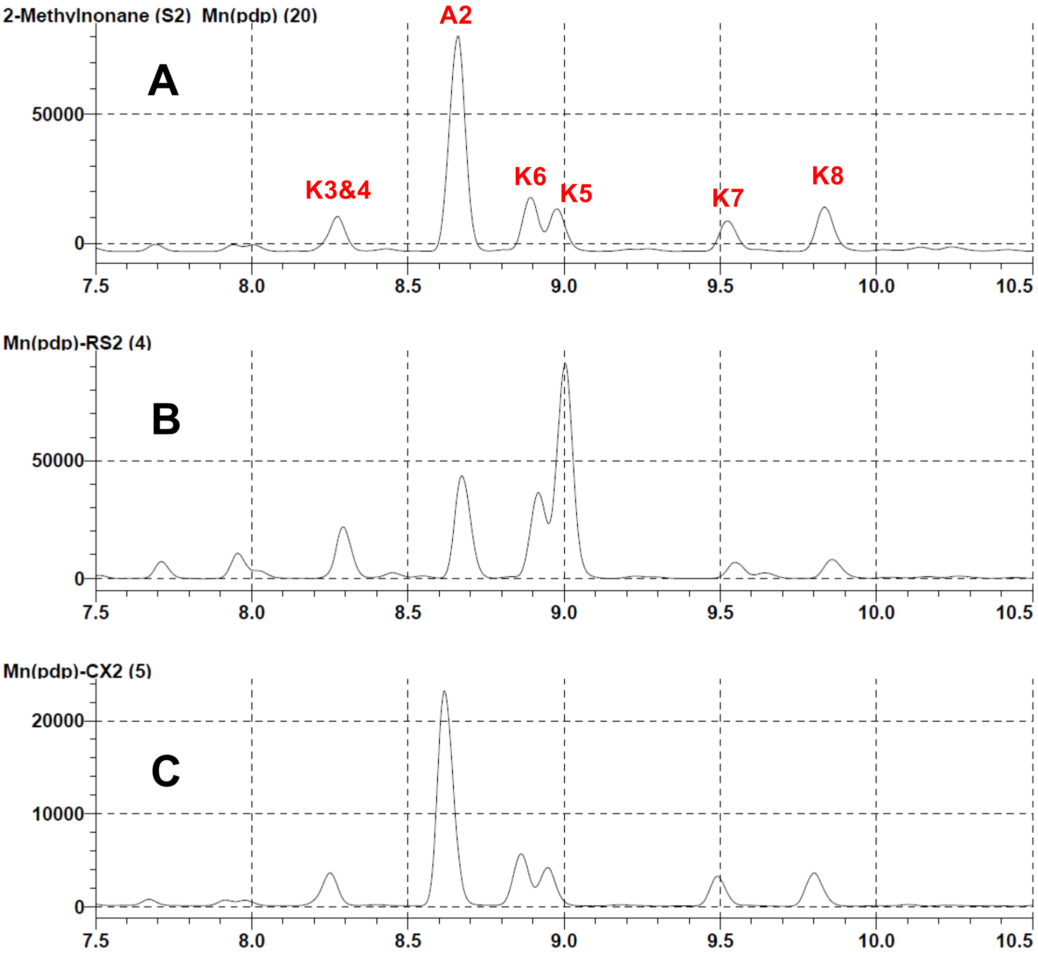

Figure S7. Comparison of pdp-catalysts. Oxidation results of 2-methylnonane (**S2**) in TFE with 2,2-DiMe-PA. Alcohol/ketone on carbon x is denominated as Ax/Kx.

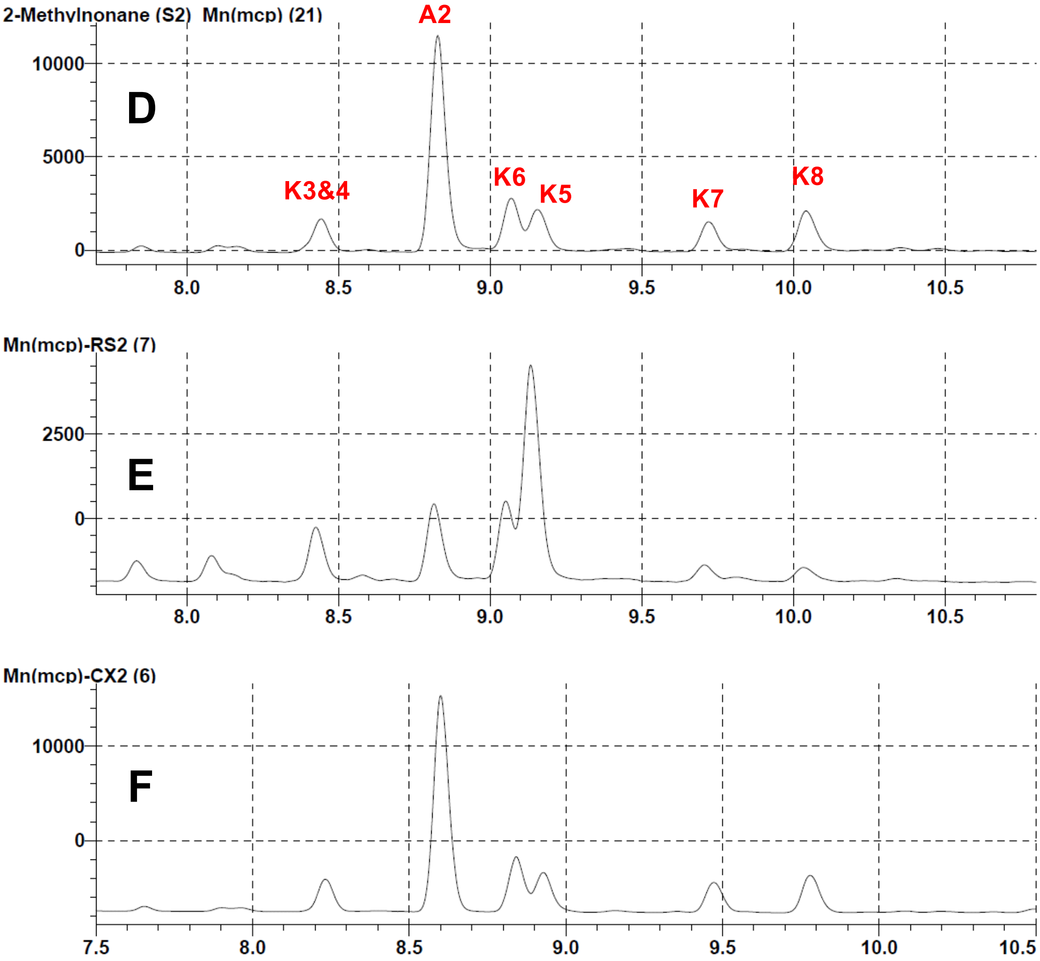

Figure S8. Comparison of mcp-catalysts. Oxidation results of 2-methylnonane (**S2**) in TFE with 2,2-DiMe-PA. Alcohol/ketone on carbon x is denominated as Ax/Kx. The difference in retention time in chromatogram F might arise from the slightly shortened GC-column.

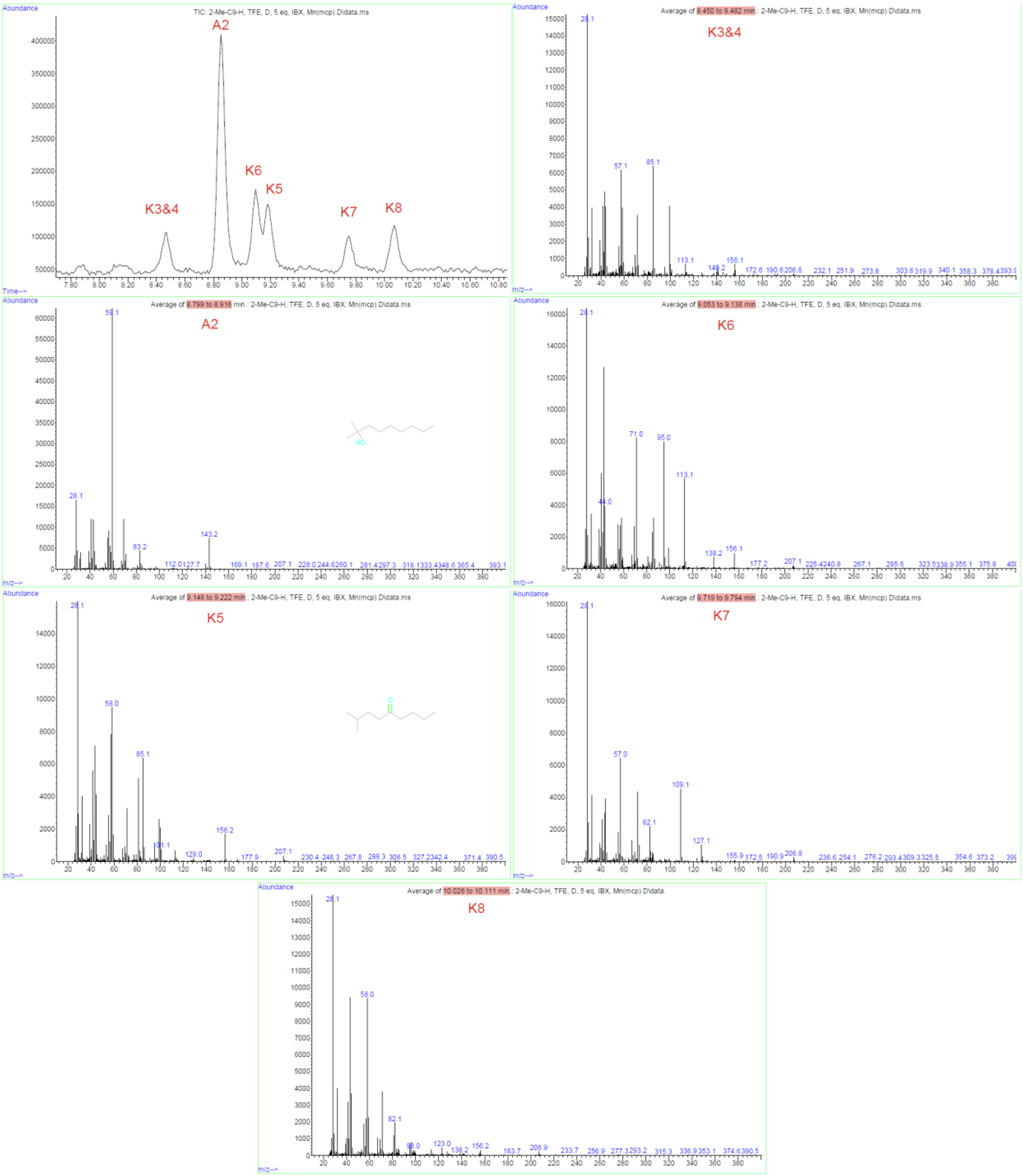

Figure S9. GC-MS analyses of the products formed in the oxidation of **S2** in TFE with 2,2-diMe-PA and catalyst Mn(mcp) (**21**). Thorough assignment of the mass fragmentation signals can be found in our previous work.[4]

1-Bromo-3,7-dimethyloctane (S3)

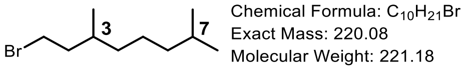

S3

The oxidation reactions were carried out following the general oxidation procedure in TFE with 5.0 equivalents of 2,2-DiMe-PA without IBX oxidation). All products were identified via GC-MS analysis and by NMR spectroscopy.[4] The achiral GC-column and a modified temperature program (60 °C for 3 min, 15 °C/min to 120 °C, 1 °C/min to 130 °C, then 20 °C/min to 250 °C and held for 5 min) were used for GC and GC-MS analyses. Comparison of the GC-chromatograms of the crude reaction mixtures obtained from the three pdp-catalysts is shown in Figure S10, and results of the three mcp-catalysts in Figure S11. The RS supramolecular catalysts **4** and **7** favor the electronically deactivated C(3) position (chromatograms B and E, respectively). In contrast to this, the site-selectivity of CX catalysts **5** and **6** (C and F) do not differ significantly from the ones of the parent catalysts **20** and **21** (A and D), leading mainly to the product alcohol A7. Identification of the products by GC-MS is presented in Figure S12.

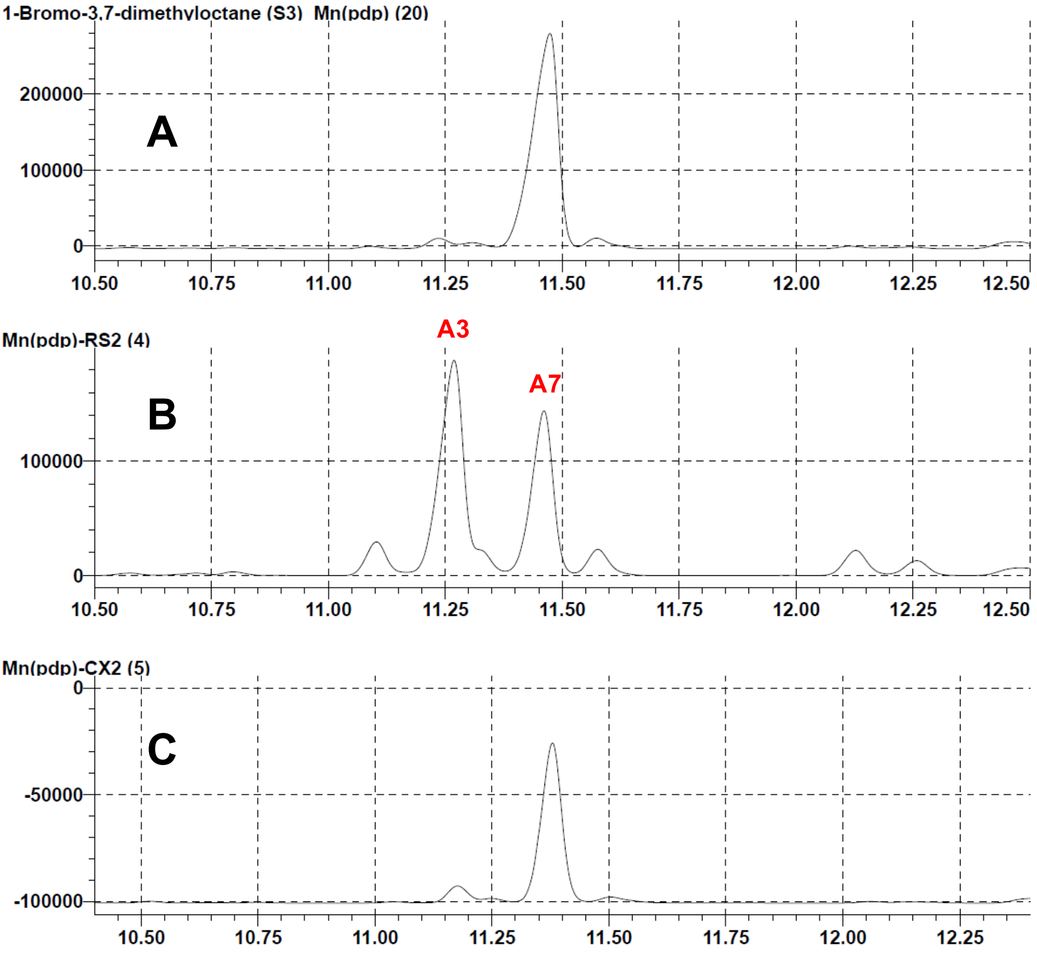

Figure S10. Comparison of pdp-catalysts. Oxidation results of 1-bromo-3,7-dimethyloctane (**S3**) in TFE with 2,2-DiMe-PA (5.0 equiv.), alcohol on carbon x is denominated as Ax. The difference in retention time in chromatogram C might arise from the slightly shortened GC-column.

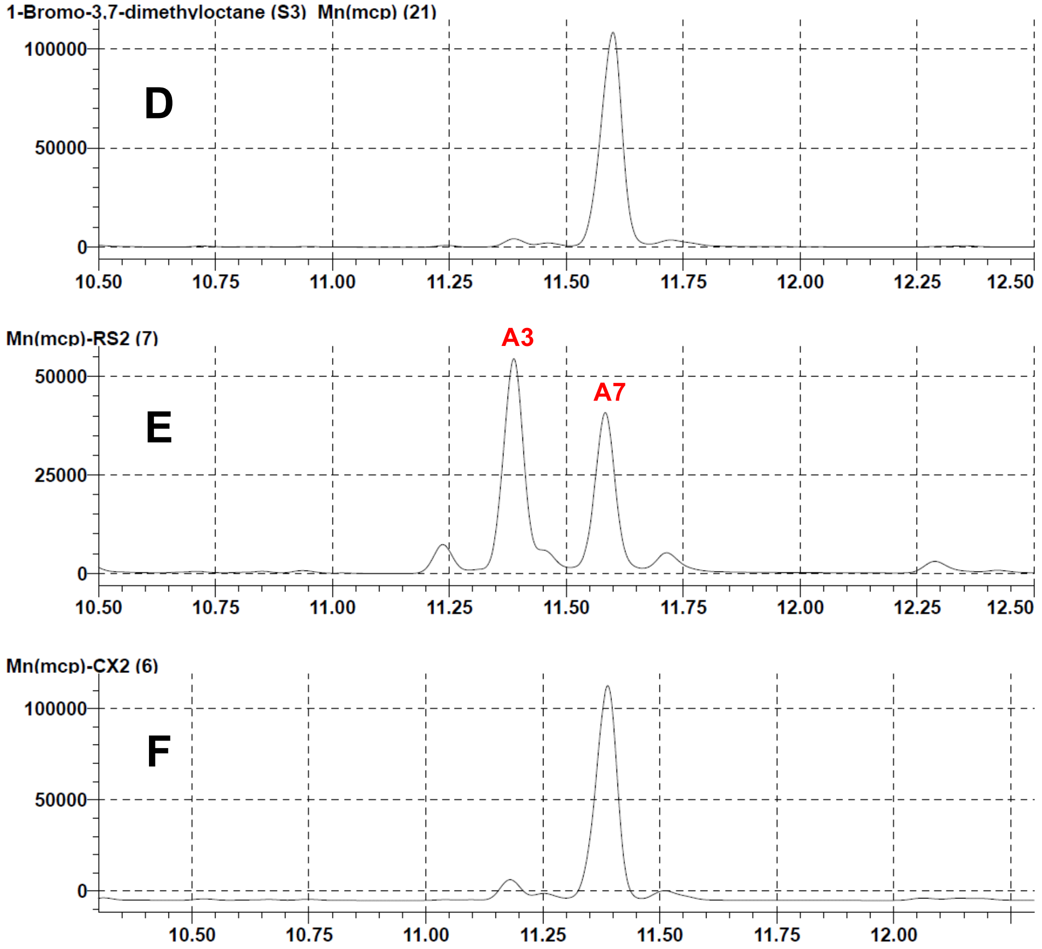

Figure S11. Comparison of mcp-catalysts. Oxidation results of 1-bromo-3,7-dimethyloctane (**S3**) in TFE with 2,2-DiMe-PA (5.0 equiv.), alcohol on carbon x is denominated as Ax. The difference in retention time in chromatogram F might arise from the slightly shortened GC-column.

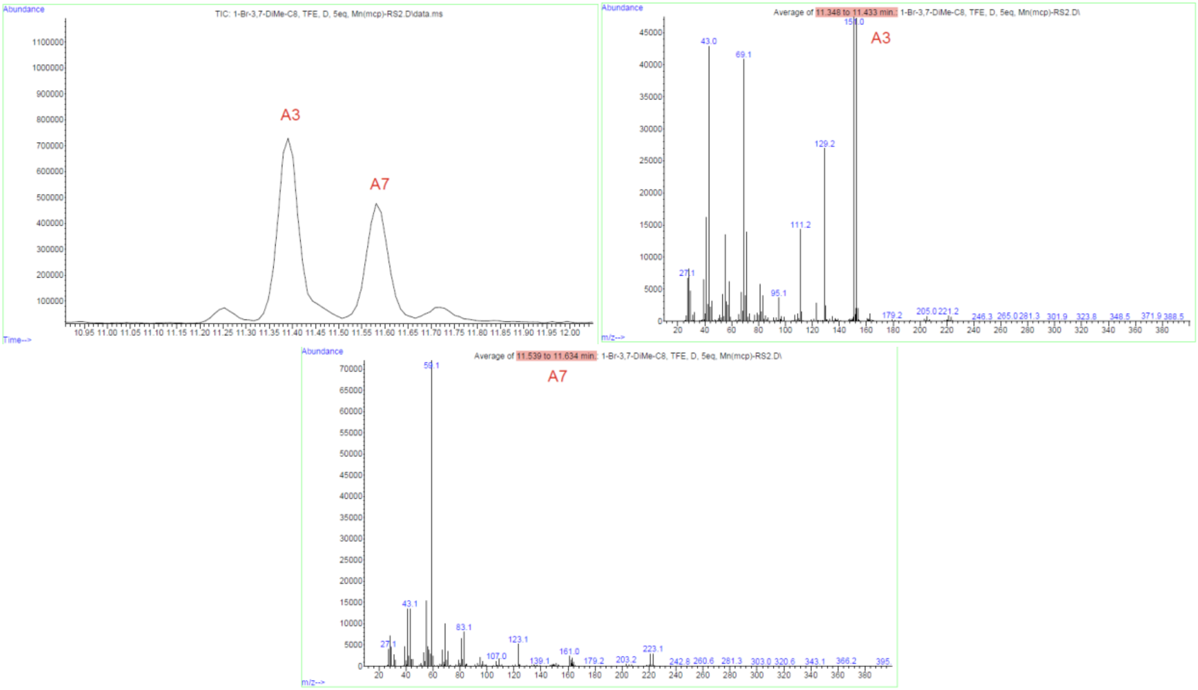

Figure S12. GC-MS analyses of the products formed in the oxidation of **S3** in TFE with 2,2-diMe-PA and catalyst Mn(mcp)-RS_2 (**7**). Thorough assignment of the mass fragmentation signals can be found in our previous work.[4]

***trans-p*-Menthane (S4)**

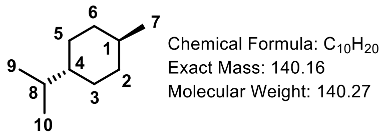

S4

The oxidation reactions were carried out following the general oxidation procedure in TFE with 2,2-DiMe-PA including the 2nd IBX oxidation step. Identification of the products by NMR, GC, GC-MS were described in our previous work in detail.[4] The chiral GC-column and a modified temperature program (60 °C for 3 min, 15 °C/min to 120 °C, 1 °C/min to 130 °C, then 20 °C/min to 250 °C and held for 5 min) were used for GC analysis. The standard temperature program was used for GC-MS measurements. Comparison of the GC-chromatograms of the crude reaction mixtures obtained from the three pdp-catalysts is shown in Figure S13, and results of the three mcp-catalysts in Figure S14. The RS supramolecular catalysts **4** and **7** prefer the sterically demanding C(4) position (chromatograms B and E, respectively). In contrast to this, the site-selectivity of CX catalysts **5** and **6** (C and F) do not differ significantly from the ones of the parent catalysts **20** and **21** (A and D), leading to the main product alcohol A8.

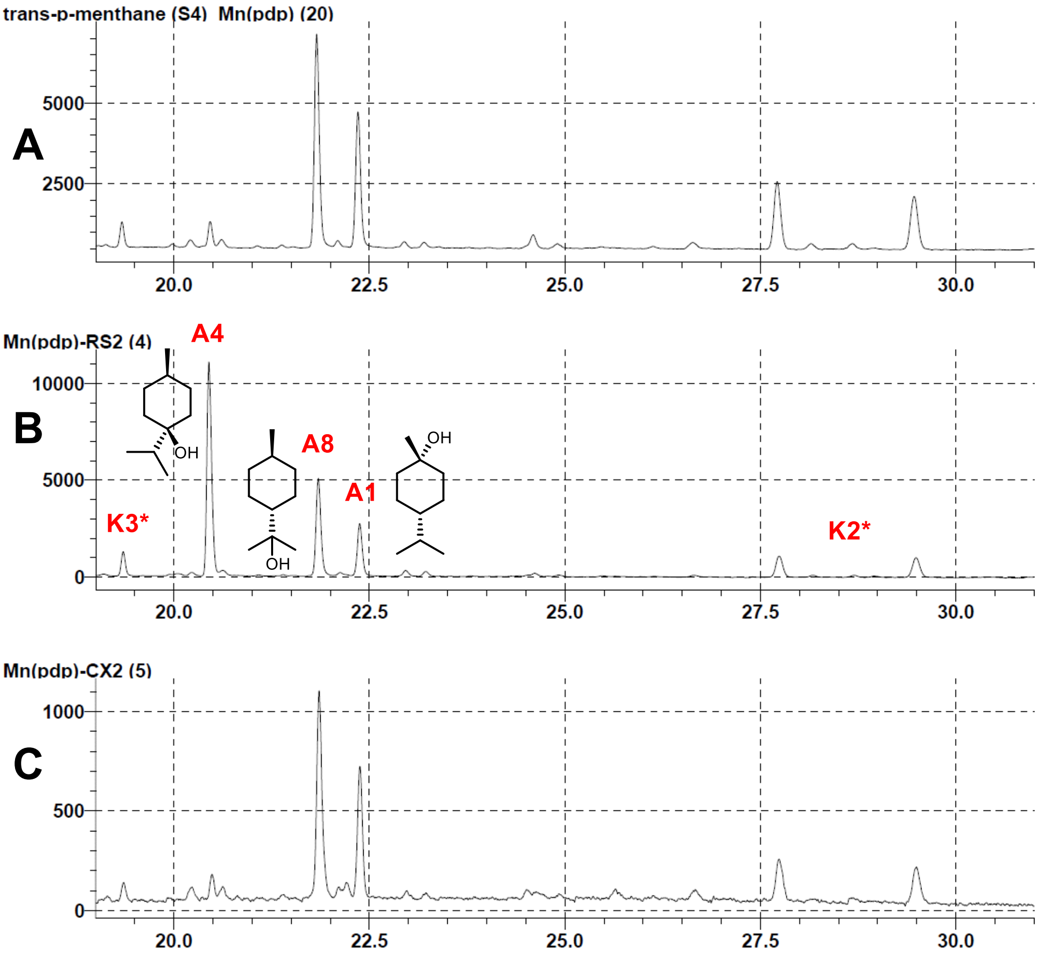

Figure S13. Comparison of pdp-catalysts. GC-chromatograms of reaction mixtures from the oxidation of *trans-p*-menthane (**S4**) on chiral column. Alcohol/ketone on carbon x is denominated as Ax/Kx. *Detailed analysis of K2 and K3 reported in our previous work.

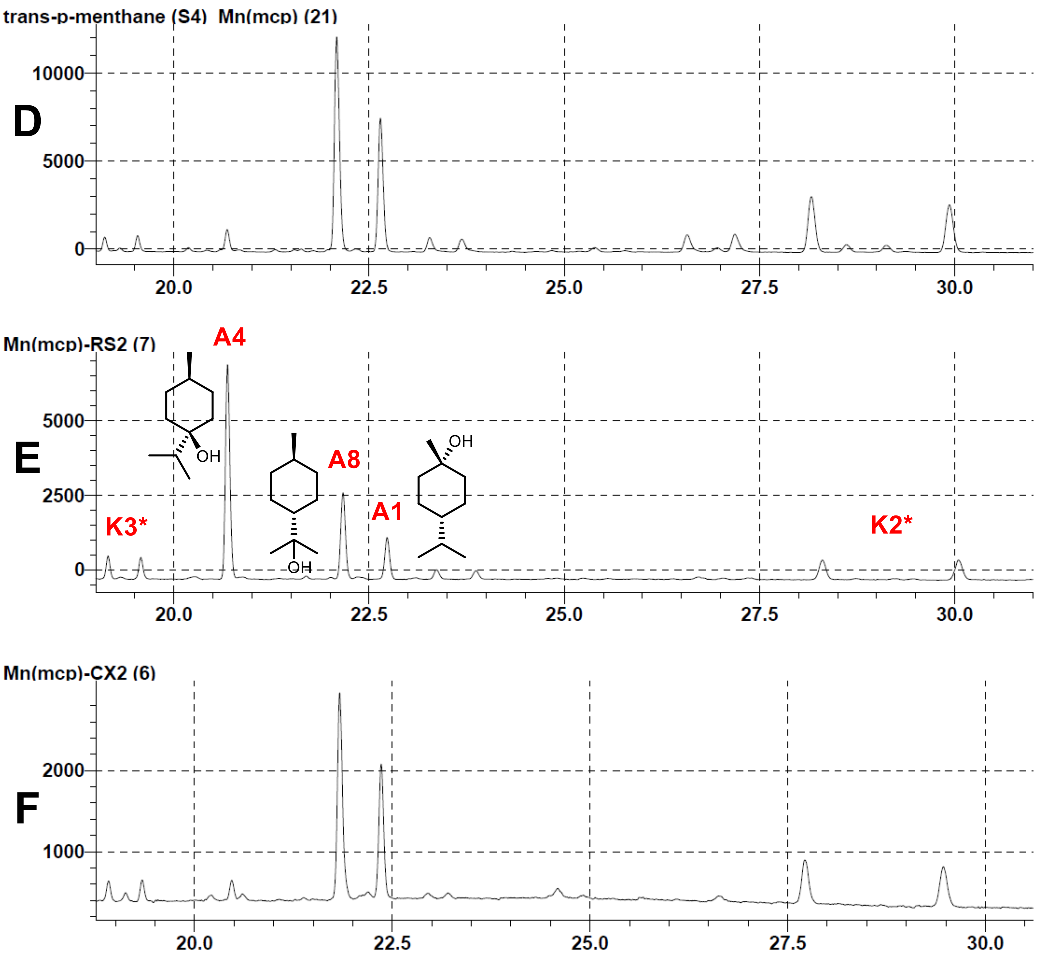

Figure S14. Comparison of mcp-catalysts. GC-chromatograms of reaction mixtures from the oxidation of *trans*-*p*-menthane (**S4**) on <u>chiral</u> column. Alcohol/ketone on carbon x is denominated as Ax/Kx. The difference in retention time in chromatogram F might arise from the slightly shortened GC-column. *Detailed analysis of K2 and K3 reported in our previous work.

4. Supplemental References

(1) Scanlon, J. T.; Willis, D. E. Calculation of Flame Ionization Detector Relative Response Factors Using the Effective Carbon Number Concept. *J. Chromatogr. Sci.* **1985**, *23* (8), 333–340. https://doi.org/10.1093/chromsci/23.8.333.

(2) Knezevic, M.; Heilmann, M.; Piccini, G. M.; Tiefenbacher, K. Overriding Intrinsic Reactivity in Aliphatic C−H Oxidation: Preferential C3/C4 Oxidation of Aliphatic Ammonium Substrates. *Angew. Chem. Int. Ed.* **2020**, *59* (30), 12387–12391. https://doi.org/10.1002/anie.202004242.

(3) Ottenbacher, R. V.; Bryliakov, K. P.; Talsi, E. P. Non-Heme Manganese Complexes Catalyzed Asymmetric Epoxidation of Olefins by Peracetic Acid and Hydrogen Peroxide. *Adv. Synth. Catal.* **2011**, *353* (6), 885–889. https://doi.org/10.1002/adsc.201100030.

(4) Lu, Y.; Knezevic, M.; Prescimone, A.; Goldfuss, B.; Tiefenbacher, K. Site-Selective C(Sp3)–H Oxidation of Alkyl Substrates Devoid of Functional Handles. *Chem* **2025**, *11* (3), 102442. https://doi.org/10.1016/j.chempr.2025.102442.

(5) De Schutter, J. W.; Zaretsky, S.; Welbourn, S.; Pause, A.; Tsantrizos, Y. S. Novel Bisphosphonate Inhibitors of the Human Farnesyl Pyrophosphate Synthase. *Bioorg. Med. Chem. Lett.* **2010**, *20* (19), 5781–5786. https://doi.org/10.1016/j.bmcl.2010.07.133.

(6) Liu, P.; Hu, Z.; DuBois, B. G.; Moyes, C. R.; Hunter, D. N.; Zhu, C.; Kar, N. F.; Zhu, Y.; Garfunkle, J.; Kang, L.; Chicchi, G.; Ehrhardt, A.; Woods, A.; Seo, T.; Woods, M.; Van Heek, M.; Dingley, K. H.; Pang, J.; Salituro, G. M.; Powell, J.; Terebetski, J. L.; Hornak, V.; Campeau, L.-C.; Lamberson, J.; Ujjainwalla, F.; Miller, M.; Stamford, A.; Wood, H. B.; Kowalski, T.; Nargund, R. P.; Edmondson, S. D. Design of Potent and Orally Active GPR119 Agonists for the Treatment of Type II Diabetes. *ACS Med. Chem. Lett.* **2015**, *6* (8), 936–941. https://doi.org/10.1021/acsmedchemlett.5b00207.

(7) Wu, C.; Wang, S.; Sun, D.; Chen, J.; Ji, W.; Wang, Y.; Nam, W.; Wang, B. Nonheme Manganese-Catalyzed Oxidative N-Dealkylation of Tertiary Amides: Manganese(IV)-Oxo Aminopyridine Cation Radical Species and Hydride Transfer Mechanism. *J. Am. Chem. Soc.* **2025**, *147* (13), 11432–11445. https://doi.org/10.1021/jacs.5c01391.

(8) Hergueta, A. R. Easy Removal of Triphenylphosphine Oxide from Reaction Mixtures by Precipitation with $CaBr_2$. *Org. Process Res. Dev.* **2022**, *26* (6), 1845–1853. https://doi.org/10.1021/acs.oprd.2c00104.

(9) Percec, V.; Bera, T. K.; De, B. B.; Sanai, Y.; Smith, J.; Holerca, M. N.; Barboiu, B.; Grubbs, R. B.; Fréchet, J. M. J. Synthesis of Functional Aromatic Multisulfonyl Chlorides and Their Masked Precursors. *J. Org. Chem.* **2001**, *66* (6), 2104–2117. https://doi.org/10.1021/jo001694x.

(10) Okunola, O. A.; Seganish, J. L.; Salimian, K. J.; Zavalij, P. Y.; Davis, J. T. Membrane-Active Calixarenes: Toward ‘Gating’ Transmembrane Anion Transport. *Tetrahedron* **2007**, *63* (44), 10743–10750. https://doi.org/10.1016/j.tet.2007.06.124.

(11) Mastalerz, M.; Dyker, G.; Flörke, U.; Henkel, G.; Oppel, I. M.; Merz, K. Oligophenylcalix[4]Arenes as Potential Precursors for Funnelenes and Calix[4]Triphenylenes: Syntheses and Preliminary Cyclodehydration Studies. *Eur. J. Org. Chem.* **2006**, *2006* (21), 4951–4962. https://doi.org/10.1002/ejoc.200600463.

(12) Dondoni, A.; Marra, A.; Scherrmann, M.-C.; Casnati, A.; Sansone, F.; Ungaro, R. Synthesis and Properties of O-Glycosyl Calix[4]Arenes (Calixsugars). *Chem. Eur. J.* **1997**, *3* (11), 1774–1782. https://doi.org/10.1002/chem.19970031108.

(13) Eastabrook, A. S.; Wang, C.; Davison, E. K.; Sperry, J. A Procedure for Transforming Indoles into Indolequinones. *J. Org. Chem.* **2015**, *80* (2), 1006–1017. https://doi.org/10.1021/jo502509s.

(14) Melot, R.; Craveiro, M. V.; Baudoin, O. Total Synthesis of (Nor)Illudalane Sesquiterpenes Based on a C(Sp3)–H Activation Strategy. *J. Org. Chem.* **2019**, *84* (20), 12933–12945. https://doi.org/10.1021/acs.joc.9b01669.

(15) Giovanardi, G.; Cattani, S.; Balestri, D.; Secchi, A.; Cera, G. Iridium-Catalyzed C–H Borylations: Regioselective Functionalizations of Calix[4]Arene Macrocycles. *J. Org. Chem.* **2024**, *89* (12), 8486–8499. https://doi.org/10.1021/acs.joc.4c00419.

(16) Knezevic, M.; Tiefenbacher, K. Tweezer-Based C−H Oxidation Catalysts Overriding the Intrinsic Reactivity of Aliphatic Ammonium Substrates. *Chem. Eur. J.* **2023**, *29* (13), e202203480. https://doi.org/10.1002/chem.202203480.

(17) Zall, A.; Bensinger, D.; Schmidt, B. Oxidative Homologation of Aldehydes to α-Ketoaldehydes by Using Iodoform, o-Iodoxybenzoic Acid, and Dimethyl Sulf-oxide. *Eur. J. Org. Chem.* **2012**, *2012* (7), 1439–1447. https://doi.org/10.1002/ejoc.201101835.

Supplemental Data

Data S1: NMR Spectra

^{1}H-NMR of **14** (500 MHz, 298 K, $CDCl_3$)

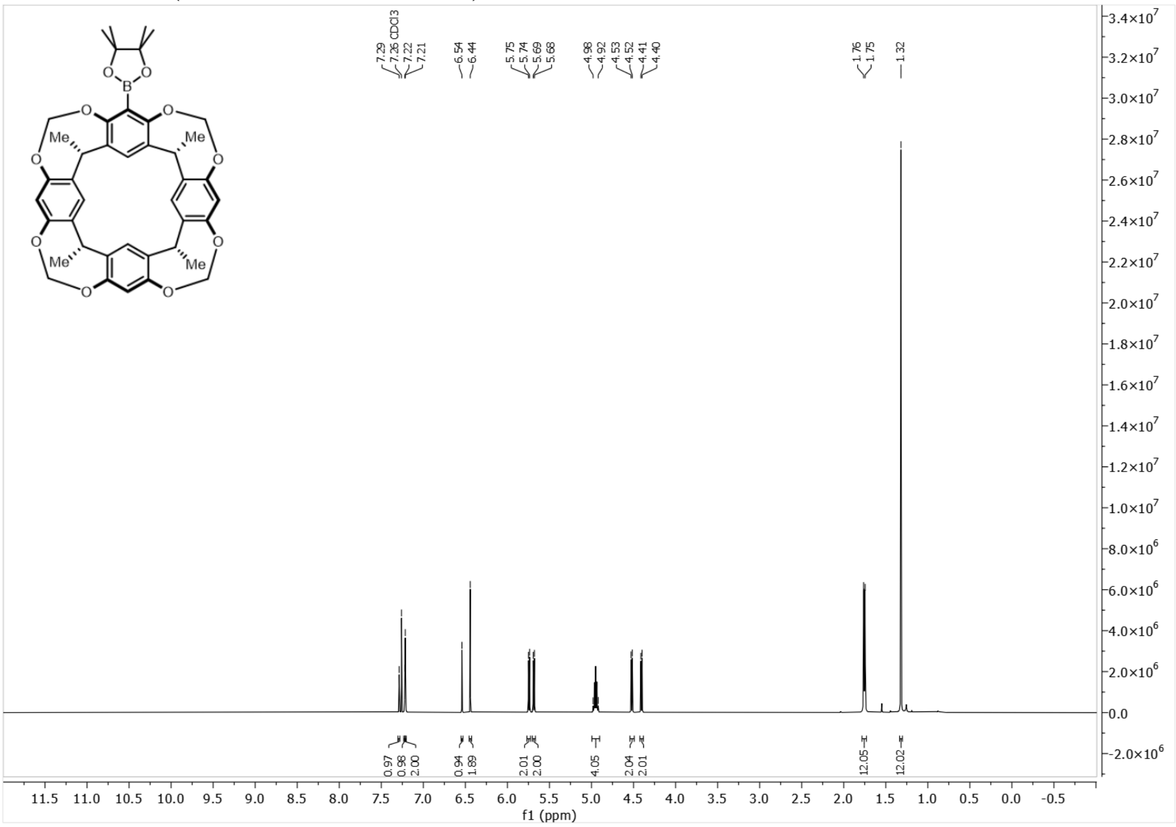

^{13}C{^{1}H}-NMR of **14** (126 MHz, 298 K, $CDCl_3$)

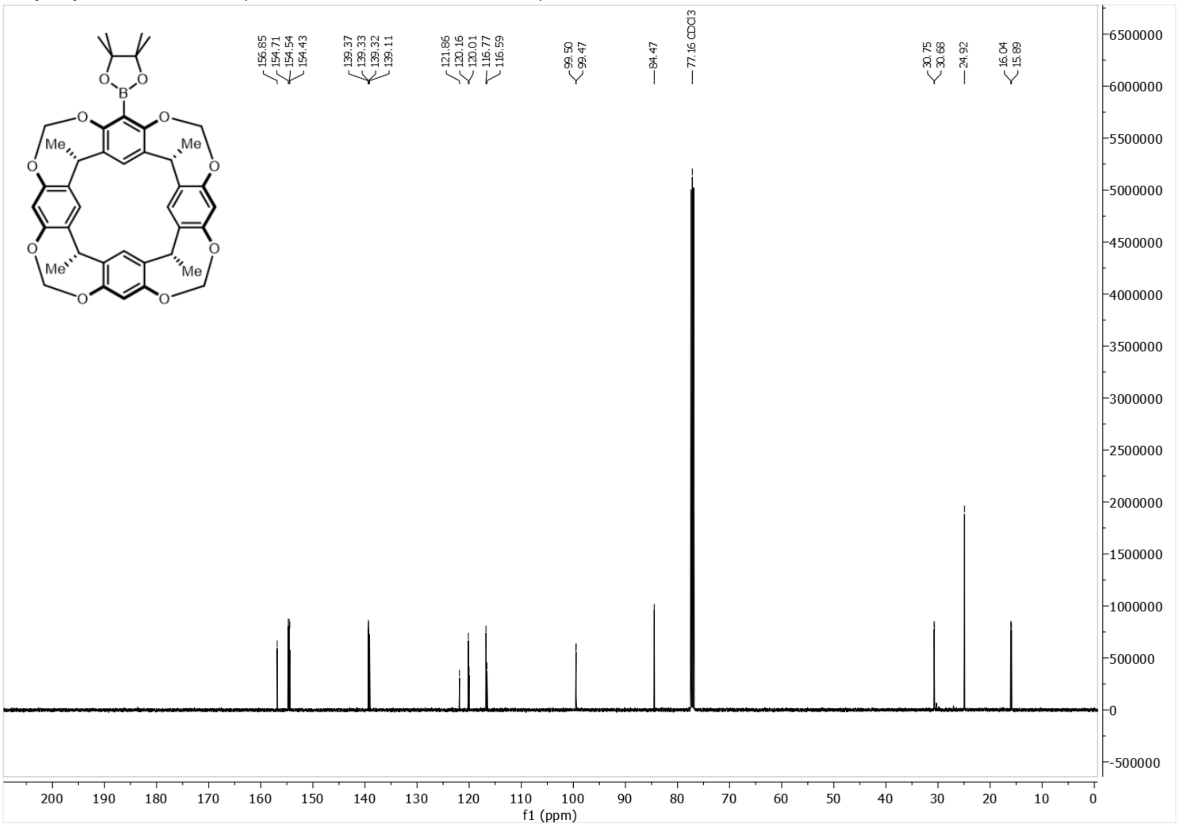

^{1}H-NMR of **11** (500 MHz, 298 K, $CDCl_3$)

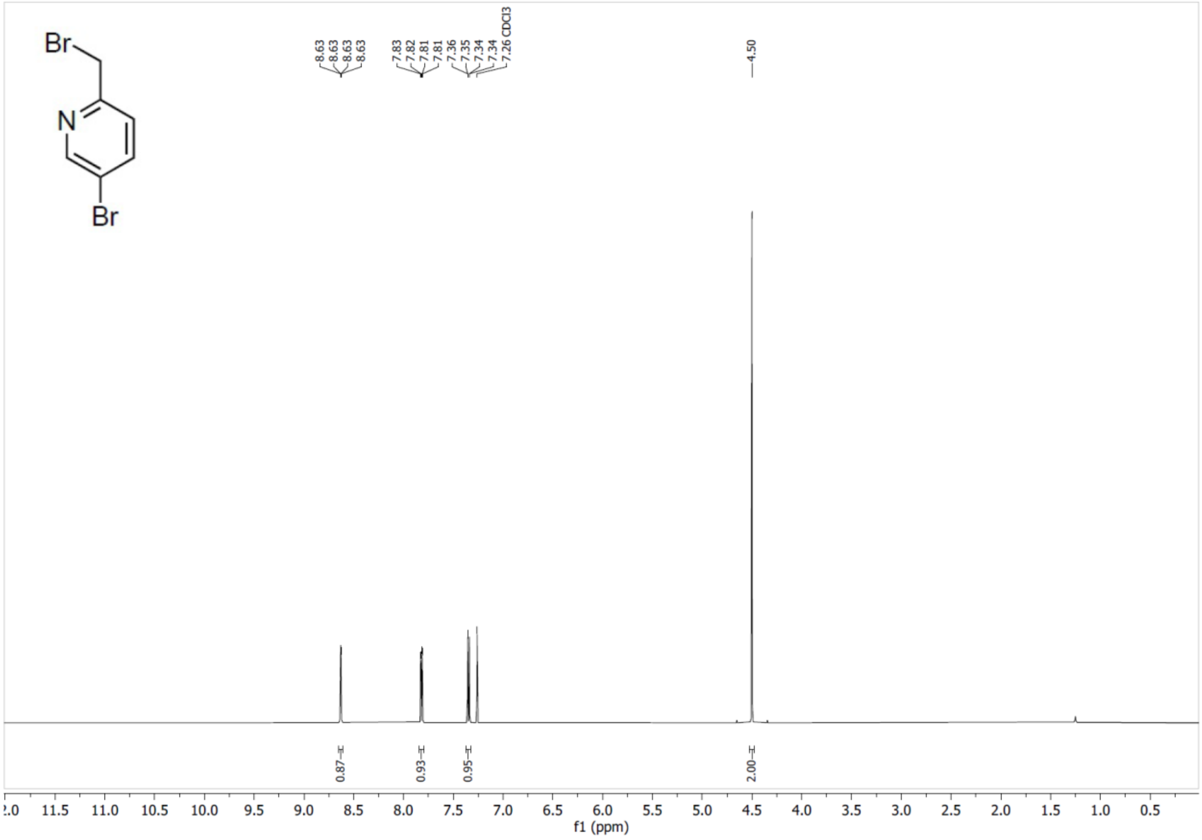

^{13}C{^{1}H}-NMR of **11** (126 MHz, 298 K, $CDCl_3$)

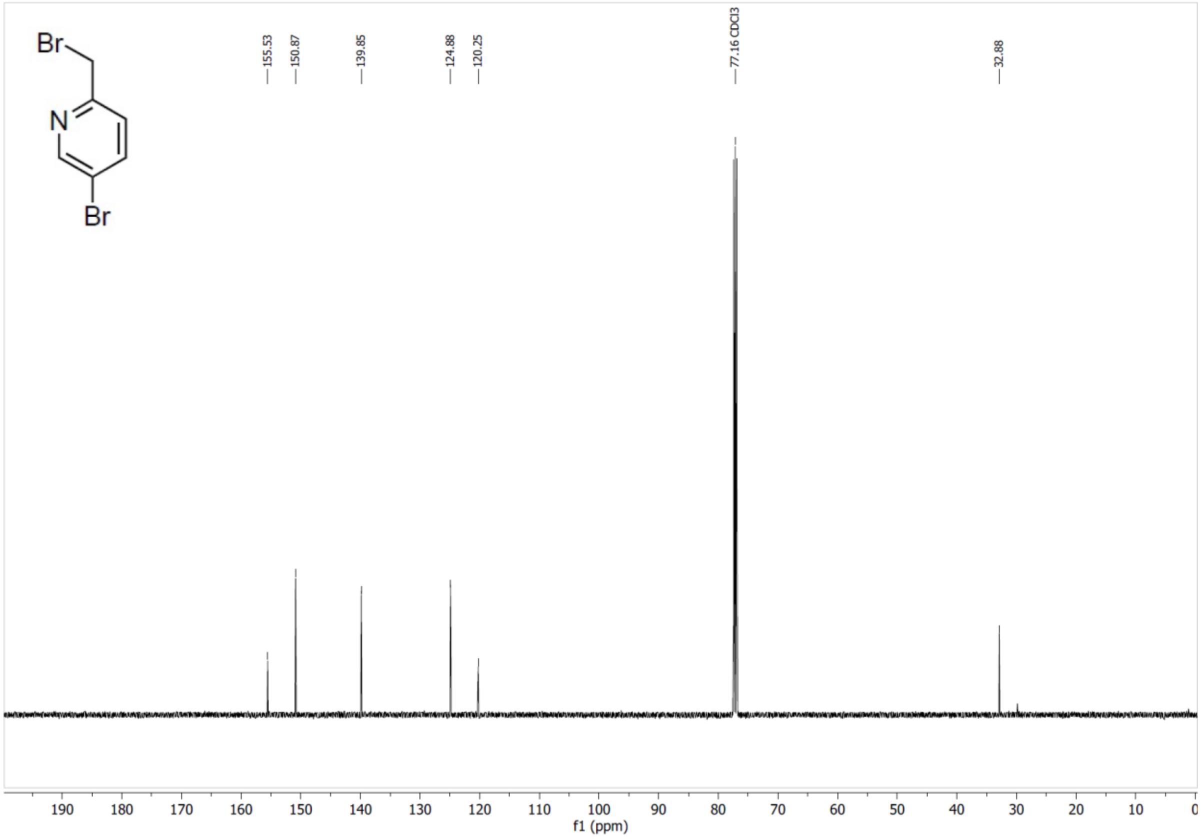

^{1}H-NMR of ligand **9** (500 MHz, 298 K, $CDCl_3$)

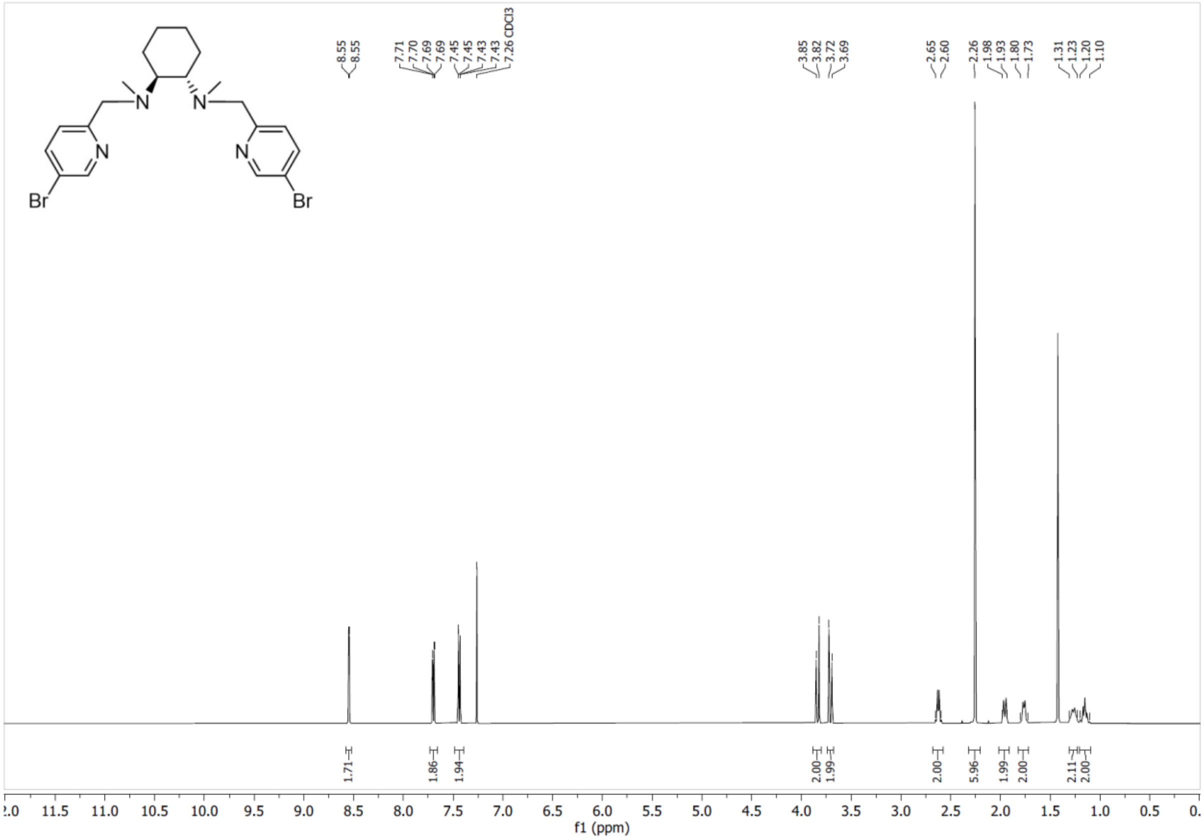

^{13}C{^{1}H}-NMR of ligand **9** (126 MHz, 298 K, $CDCl_3$)

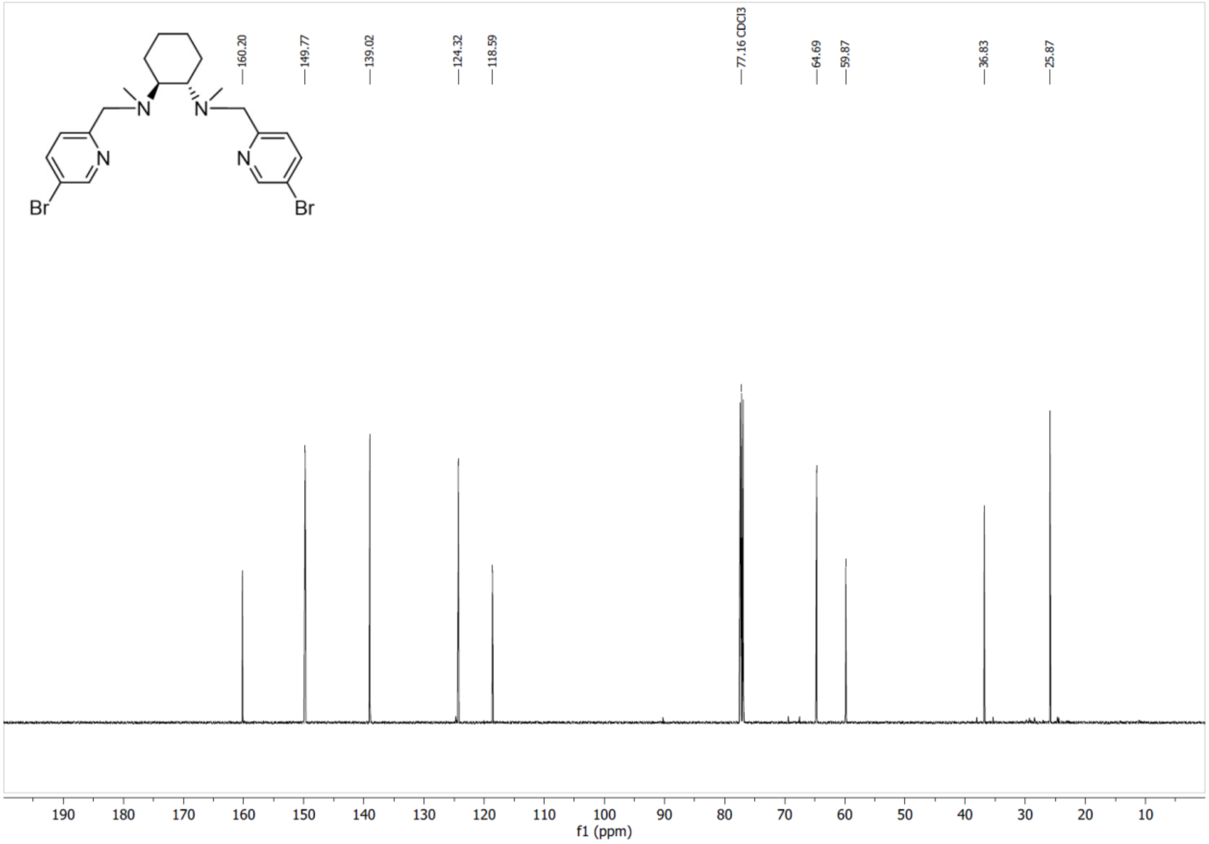

^{1}H-NMR of ligand **19** (500 MHz, 298 K, $CDCl_3$)

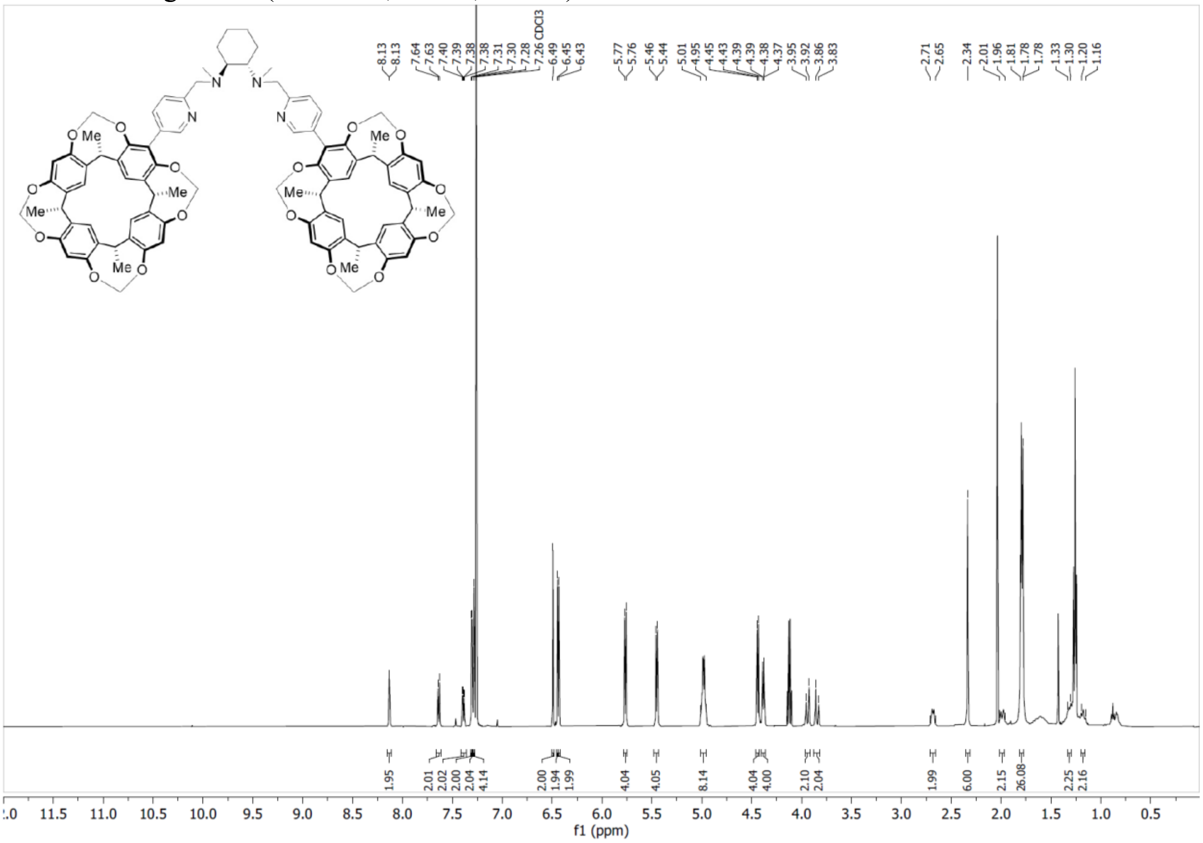

^{13}C{^{1}H}-NMR of ligand **19** (126 MHz, 298 K, $CDCl_3$)

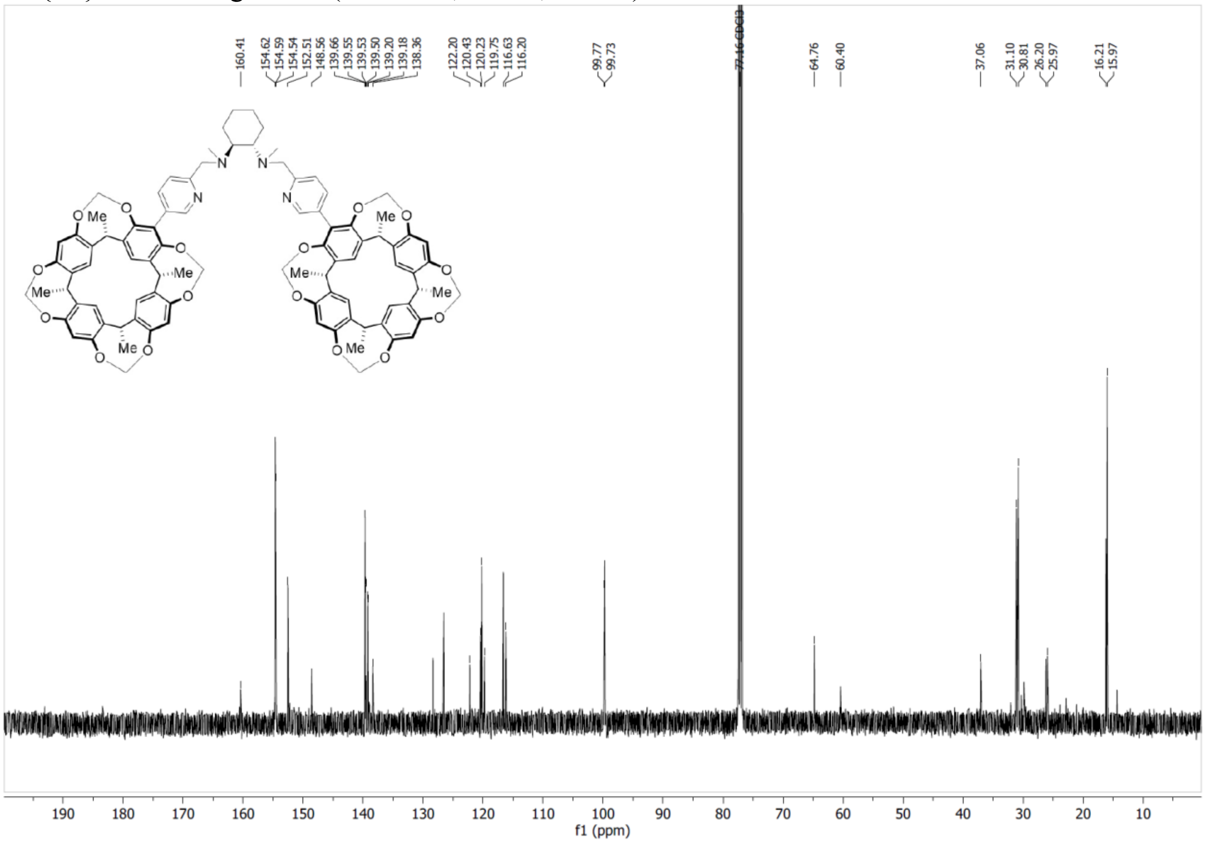

^{1}H-NMR of ligand **8** (500 MHz, 298 K, $CDCl_3$)

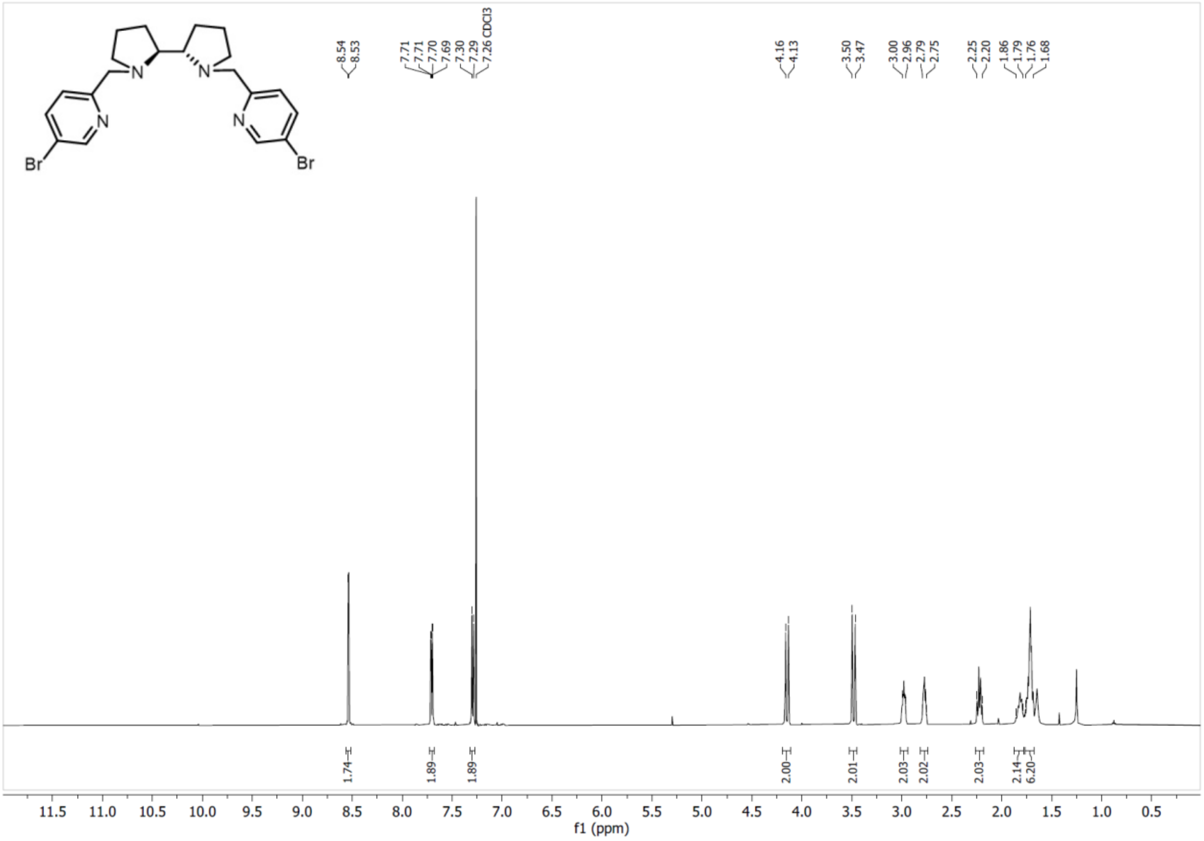

^{13}C{^{1}H}-NMR of ligand **8** (126 MHz, 298 K, $CDCl_3$)

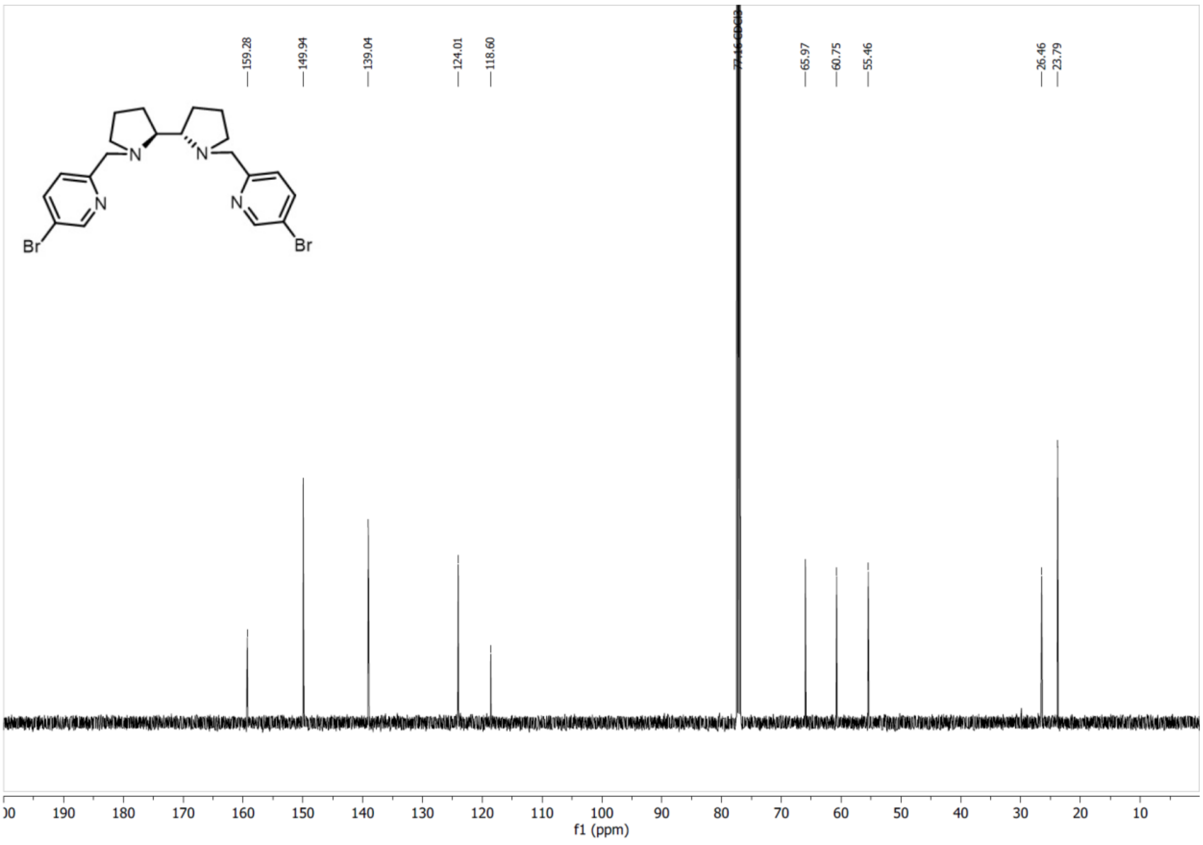

^{1}H-NMR of ligand **16** (500 MHz, 298 K, $CDCl_3$)

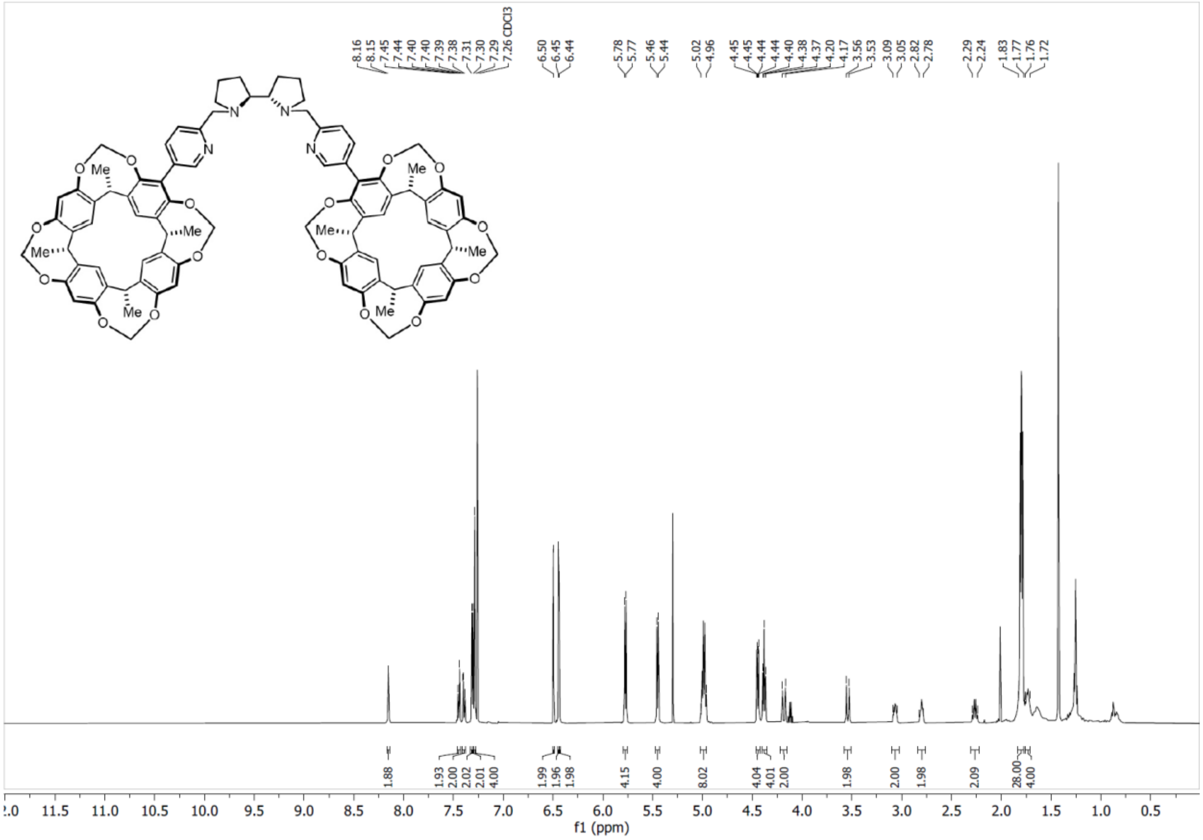

^{13}C{^{1}H}-NMR of ligand **16** (126 MHz, 298 K, $CDCl_3$)

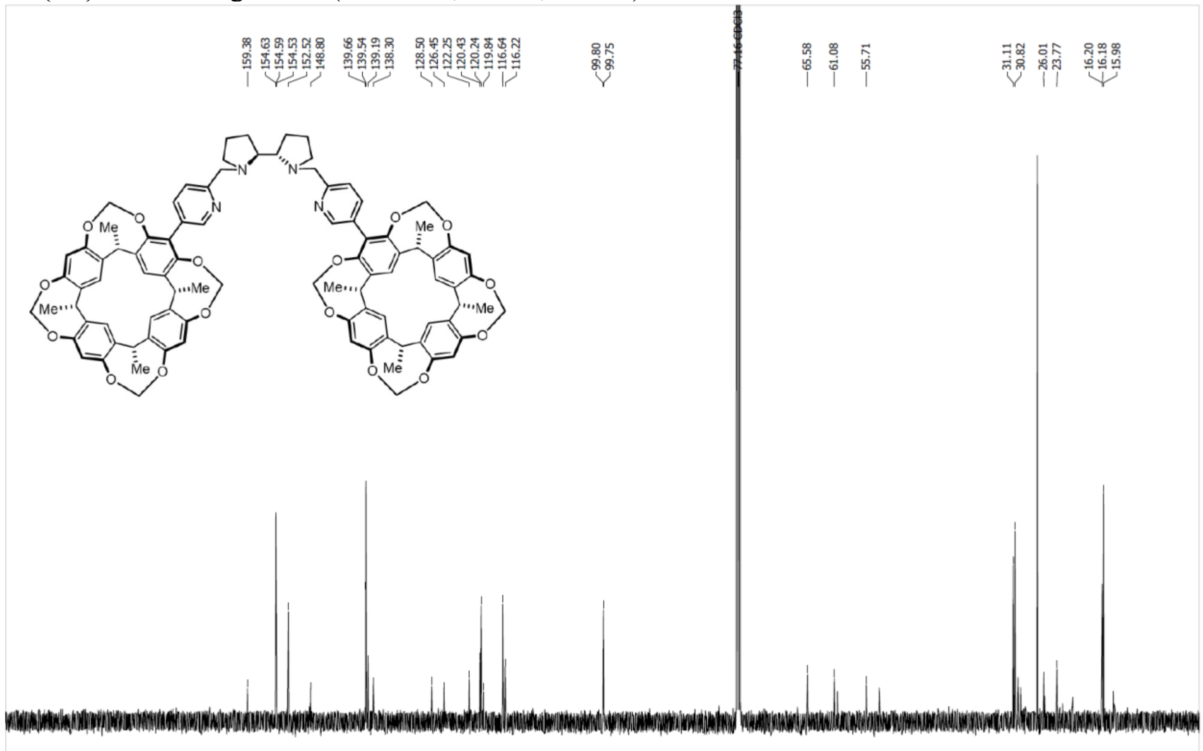

^{1}H-NMR of **SI-2** (500 MHz, 298 K, $CDCl_3$)

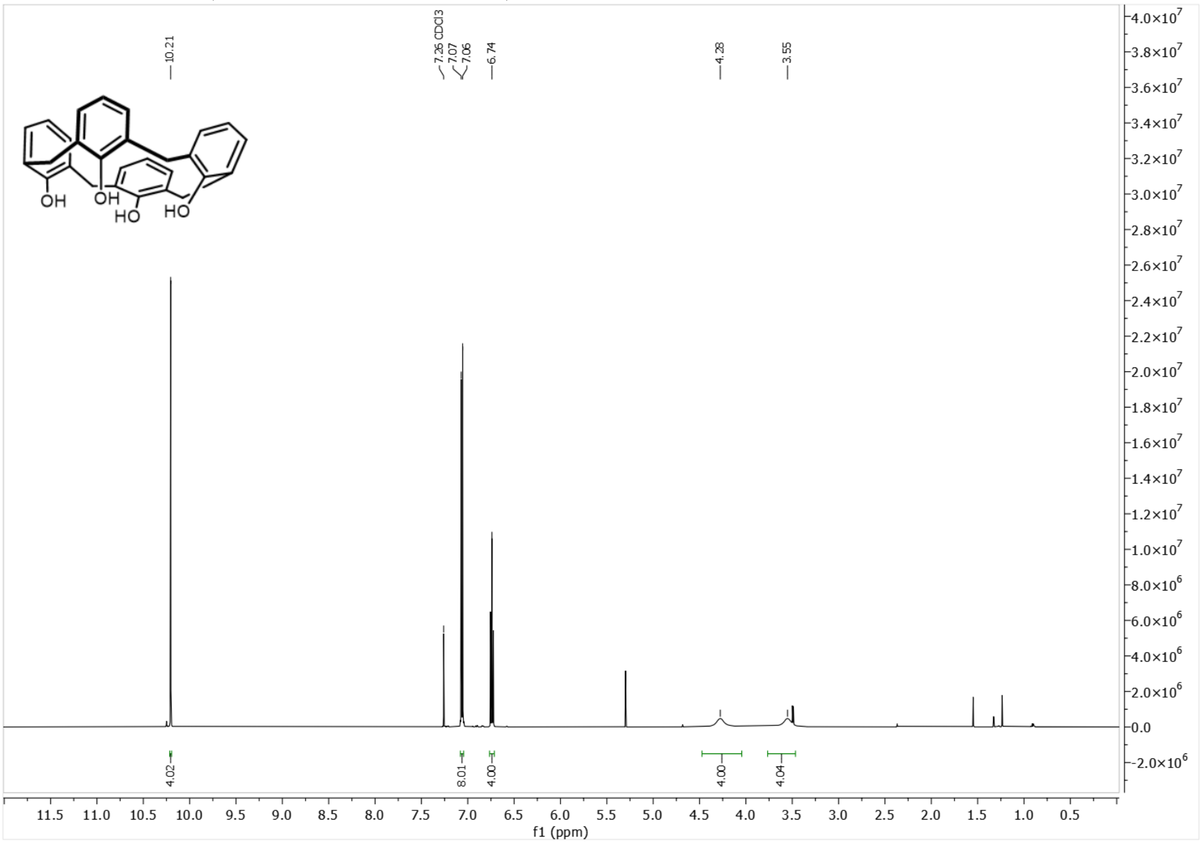

^{13}C{^{1}H}-NMR of **SI-2** (126 MHz, 298 K, $CDCl_3$)

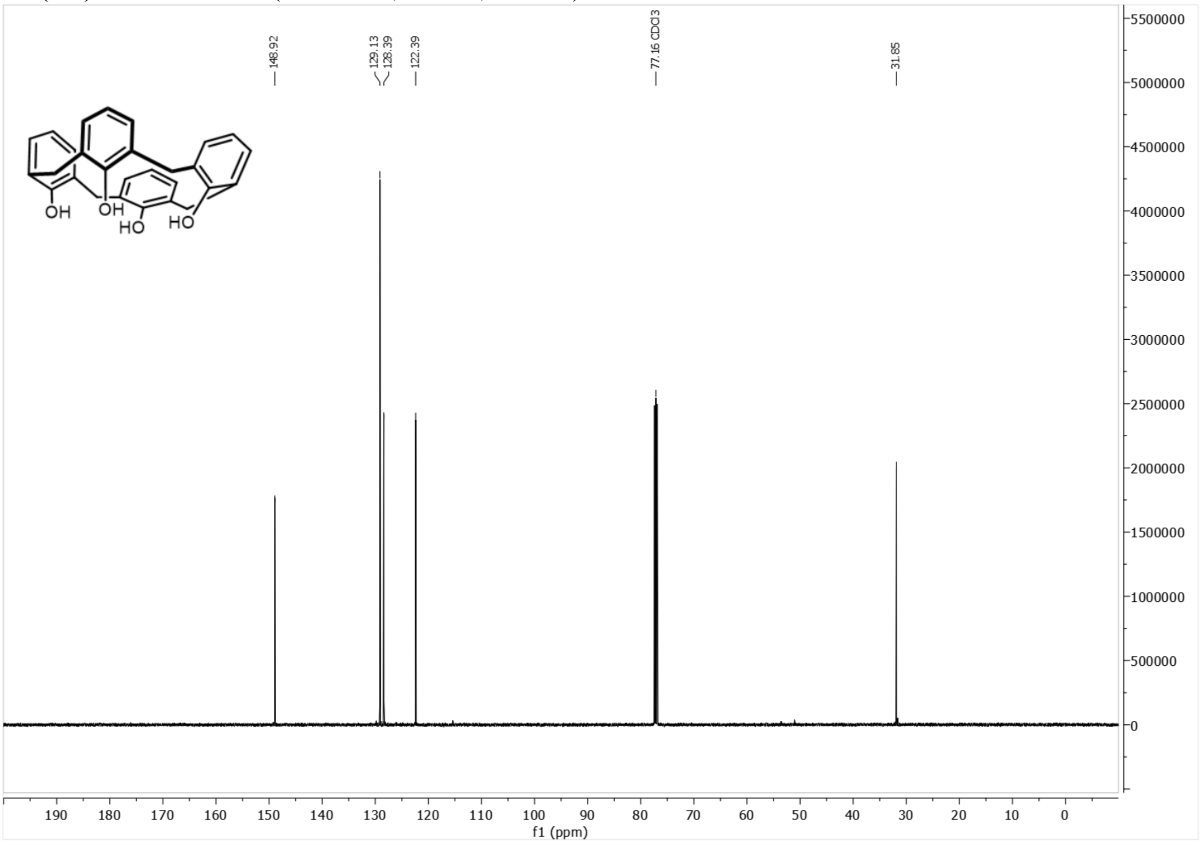

^{1}H-NMR of **SI-3** (500 MHz, 298 K, $CDCl_3$)

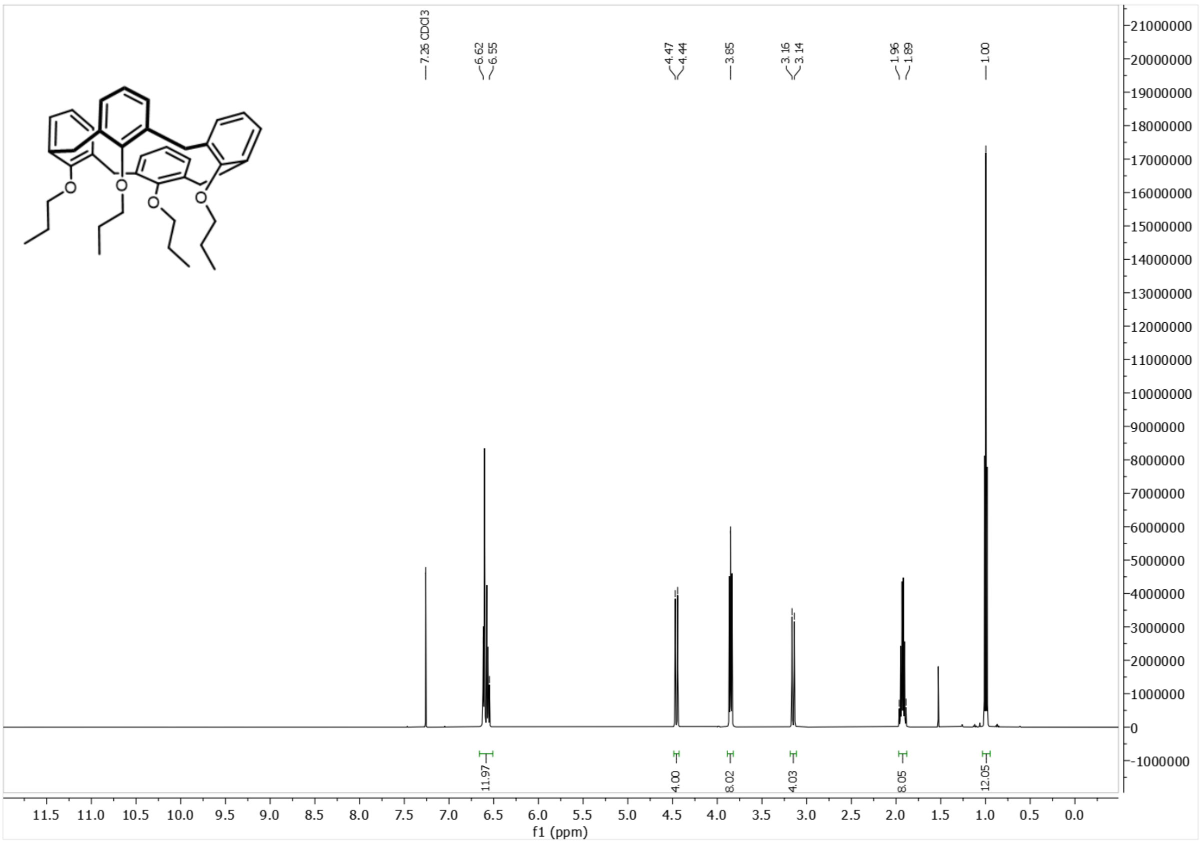

^{13}C{^{1}H}-NMR of **SI-3** (126 MHz, 298 K, $CDCl_3$)

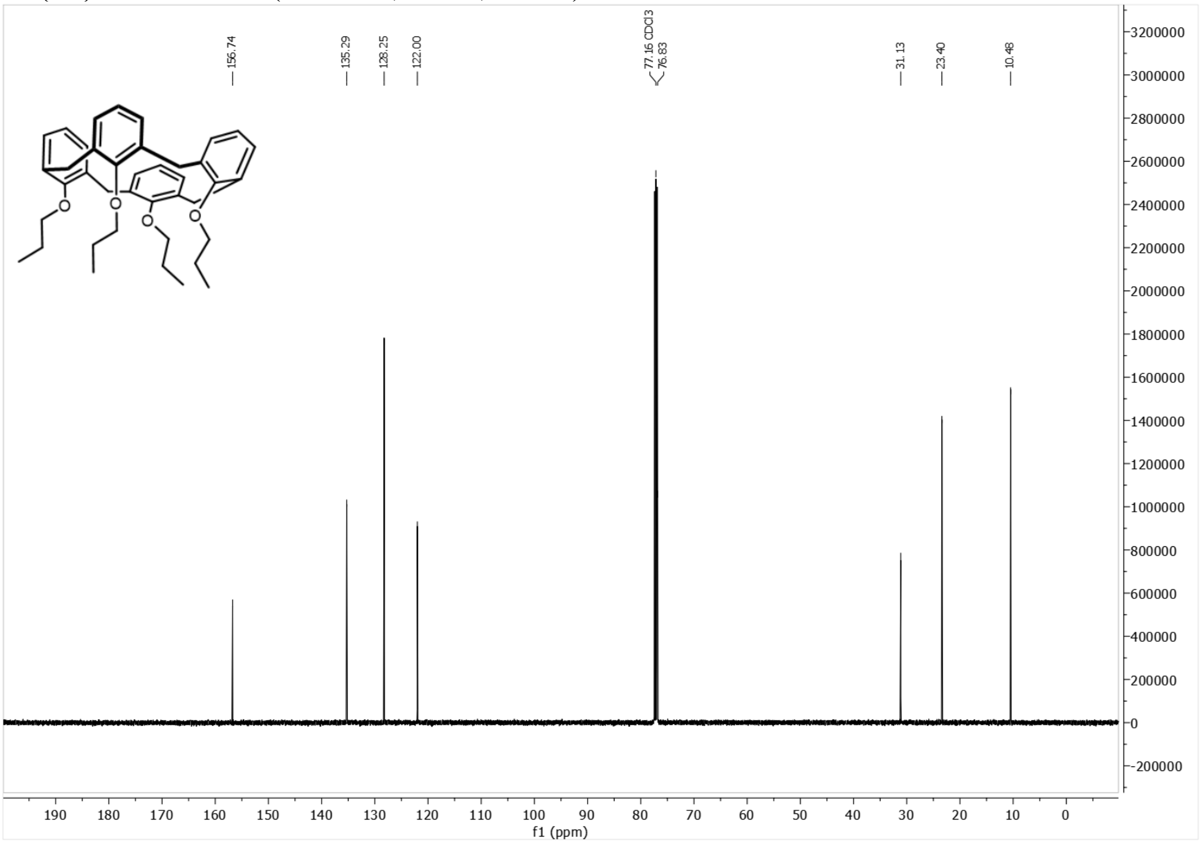

^{1}H-NMR of **15** (500 MHz, 298 K, $CDCl_3$)

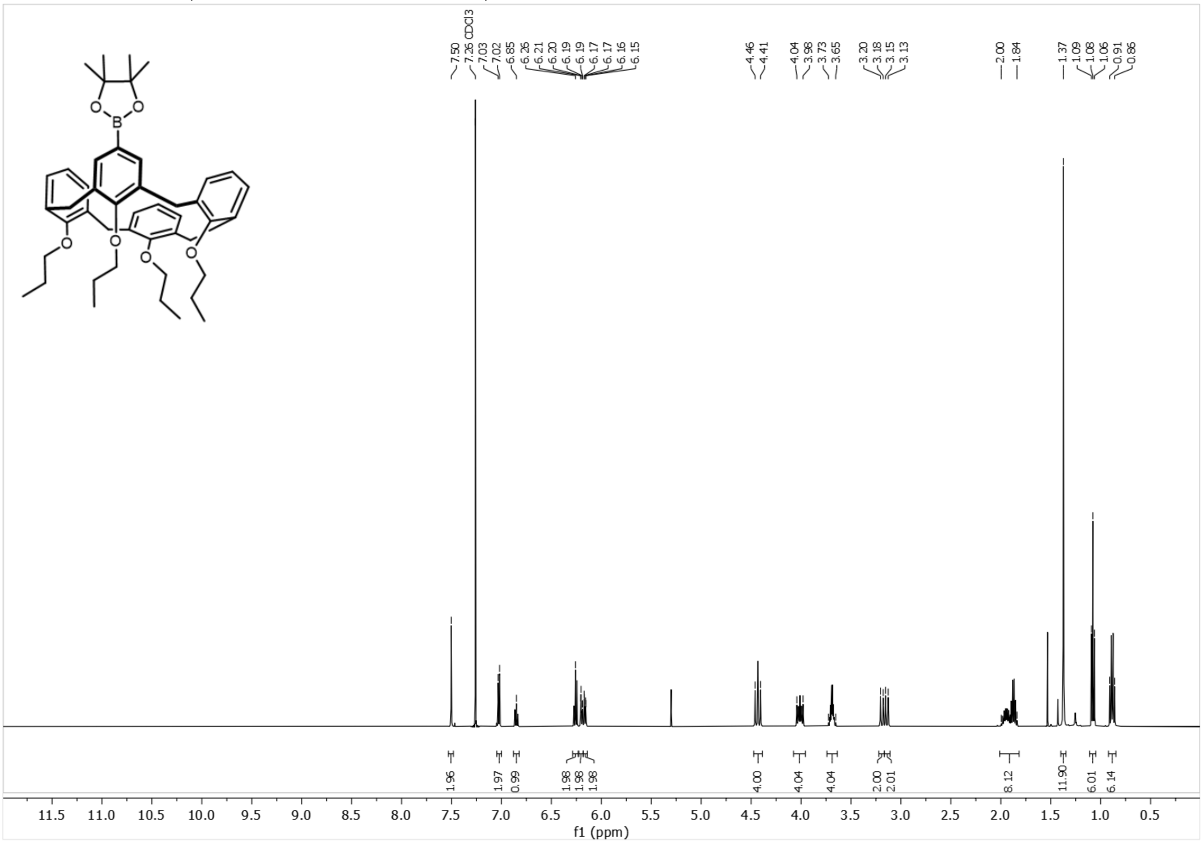

^{13}C{^{1}H}-NMR of **15** (126 MHz, 298 K, $CDCl_3$)

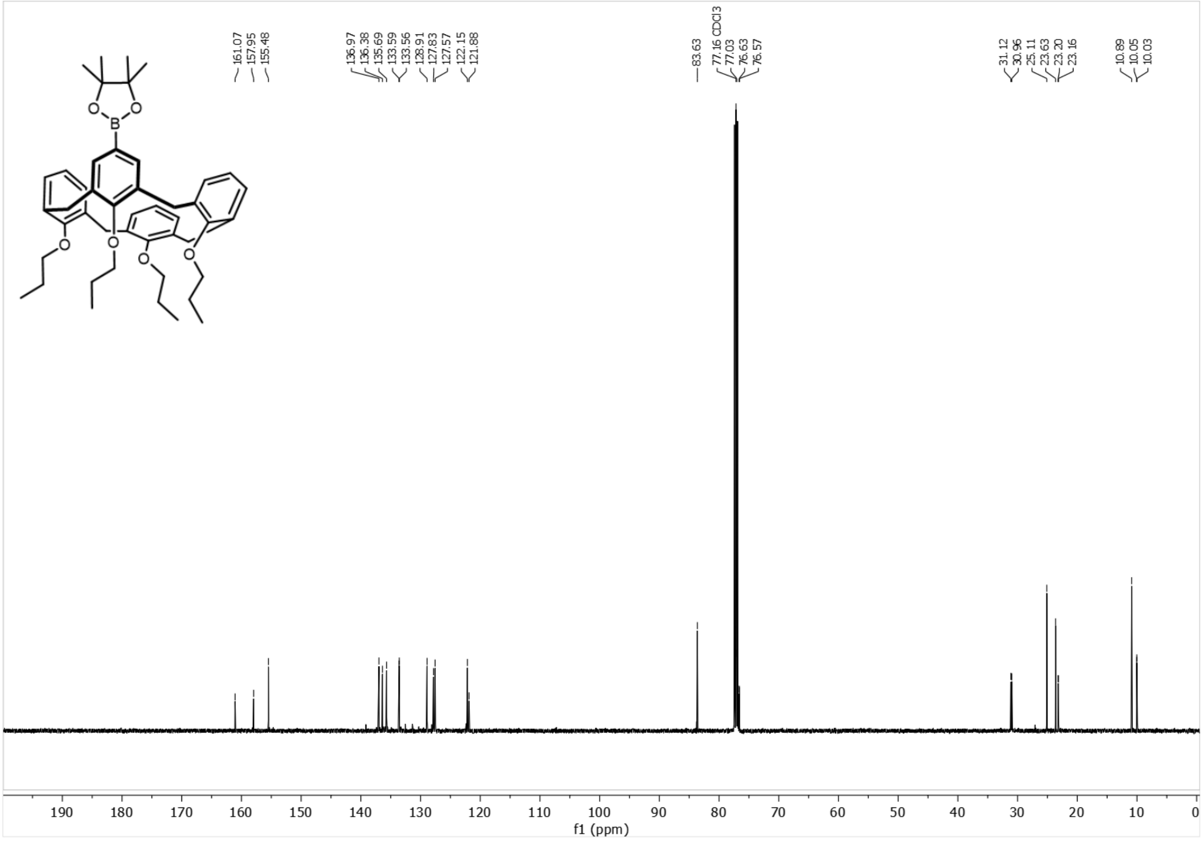

^{1}H-NMR of **17** (500 MHz, 298 K, $CDCl_3$)

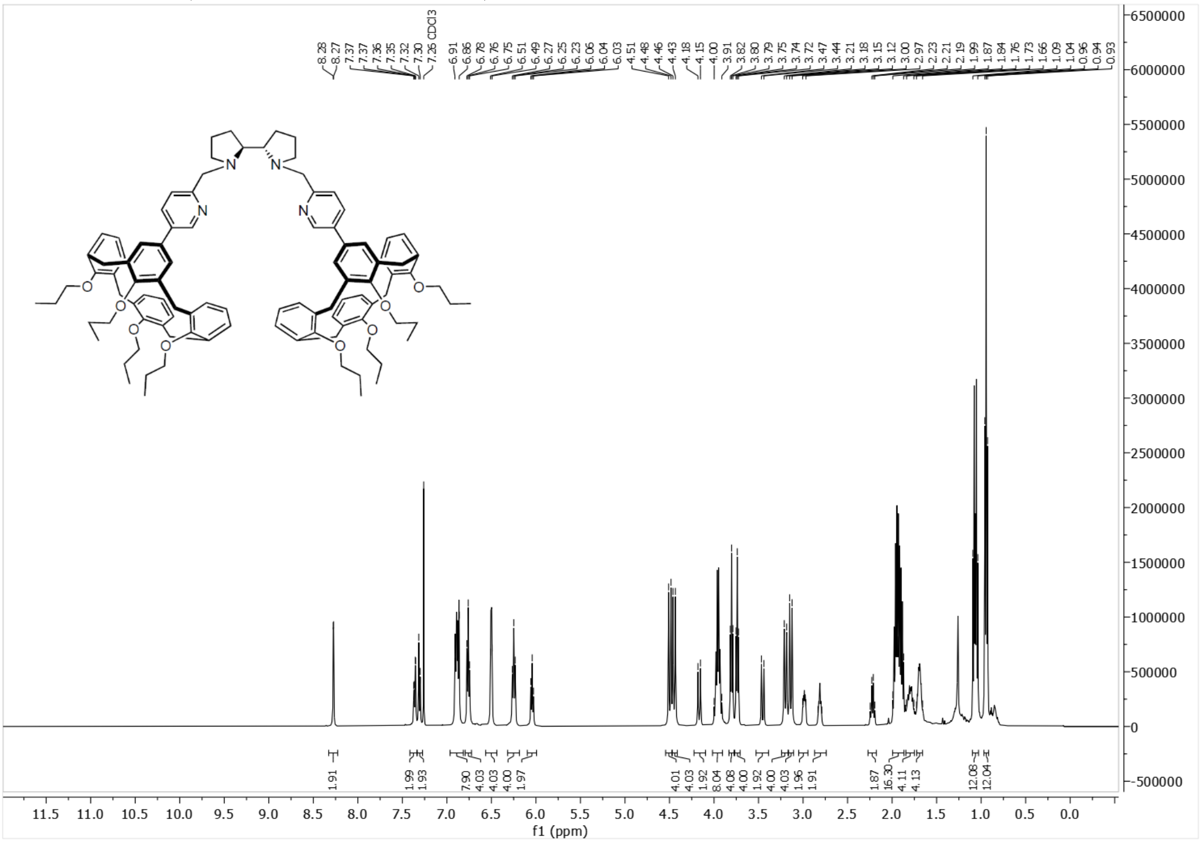

$^{13}C\{^{1}H\}$-NMR of **17** (126 MHz, 298 K, $CDCl_3$)

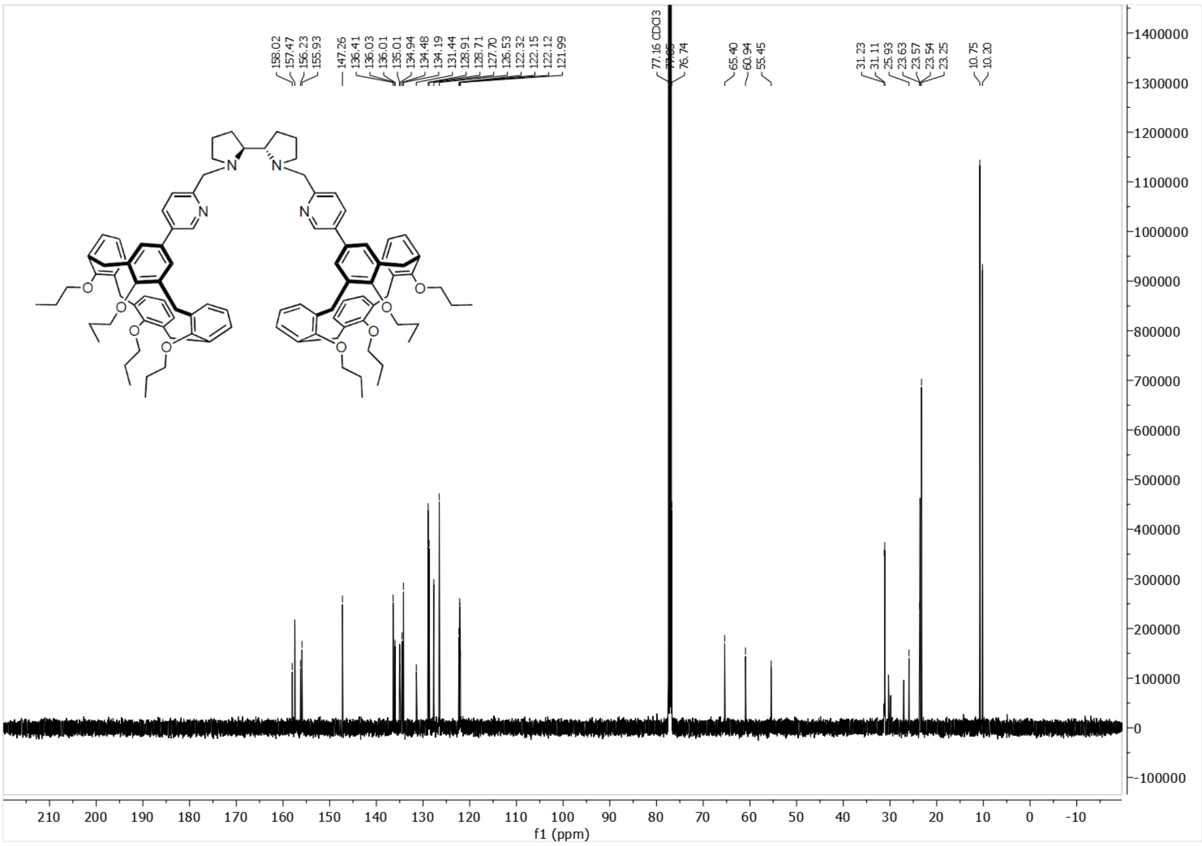

^{1}H-NMR of **18** (500 MHz, 298 K, $CDCl_3$)

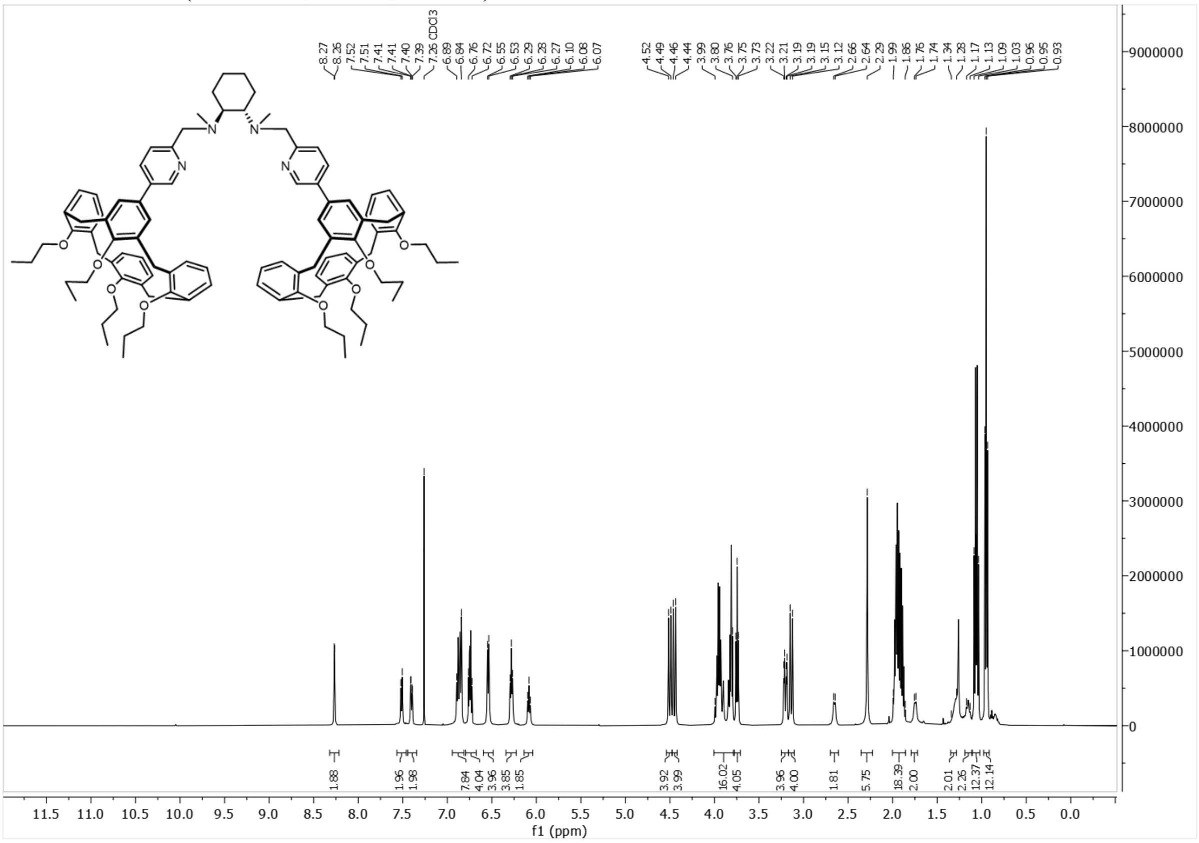

^{13}C{^{1}H}-NMR of **18** (126 MHz, 298 K, $CDCl_3$)

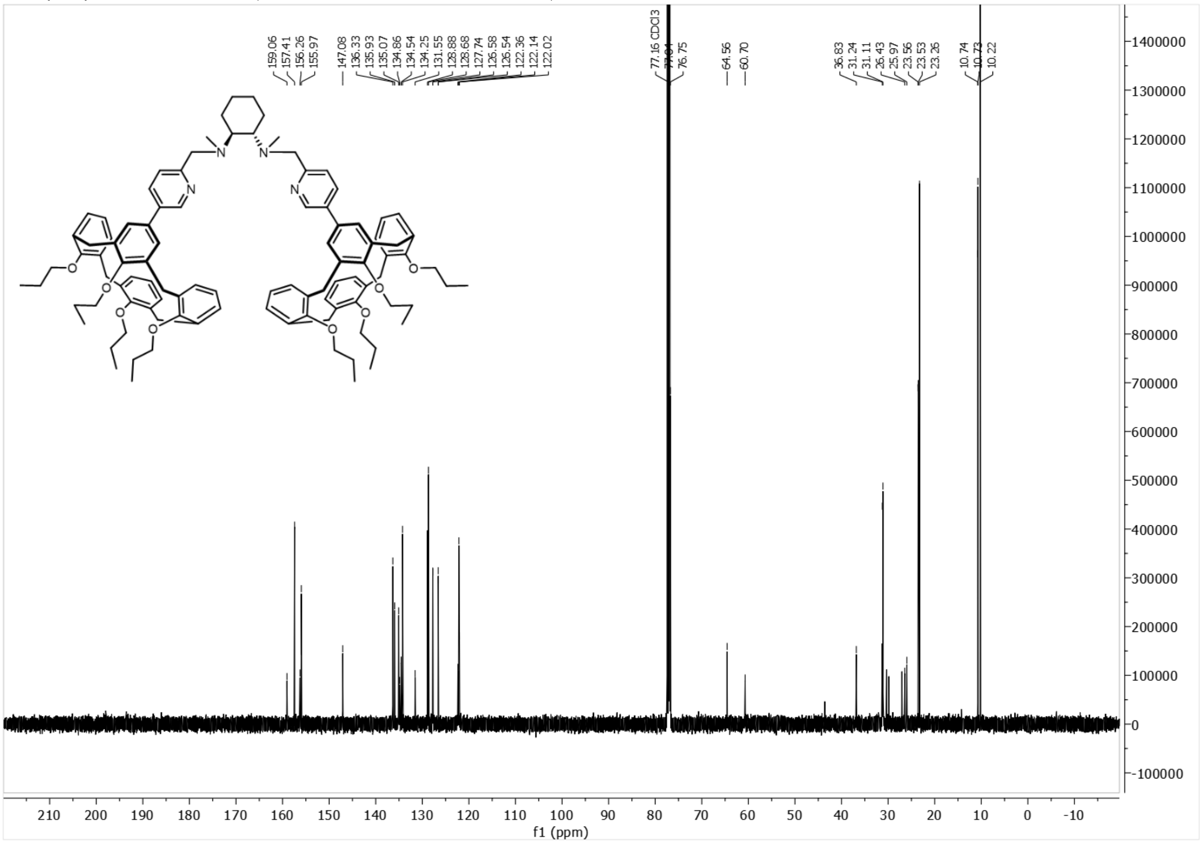

Supplement: Supplementary file 1 [file gg6c00006_si_001.pdf]
